# *Pulicaria dysenterica* (L.) Bernh.—Rightfully Earned Name? Identification and Biological Activity of New 3-Methoxycuminyl Esters from *P. dysenterica* Essential Oil

**DOI:** 10.3390/plants11233340

**Published:** 2022-12-01

**Authors:** Niko S. Radulović, Marko Z. Mladenović, Dušan R. Vukićević, Nikola M. Stojanović, Pavle J. Randjelović, Zorica Z. Stojanović-Radić, Fabio Boylan

**Affiliations:** 1 Department of Chemistry, Faculty of Sciences and Mathematics, University of Nis, Višegradska 33, 18000 Nis, Serbia; 2 Faculty of Medicine, University of Kragujevac, Svetozara Markovića 69, 34000 Kragujevac, Serbia; 3 Department of Physiology, Faculty of Medicine, University of Nis, Zorana Ðinđica 81, 18000 Nis, Serbia; 4 Department of Biology and Ecology, Faculty of Sciences and Mathematics, University of Nis, Visegradska 33, 18000 Nis, Serbia; 5 School of Pharmacy and Pharmaceutical Sciences, Panoz Institute, Trinity College, Westland Row, 2 Dublin, Ireland

**Keywords:** *Pulicaria dysenterica*, essential oil, 3-methoxycuminyl esters, antimicrobial activity, acetylcholinesterase inhibitory activity, antispasmodic activity

## Abstract

Motivated by the ethnopharmacological use of *Pulicaria dysenterica*, in the present study, the antimicrobial potential of the extracted essential oil was investigated against a panel of eighteen microorganism strains. Additionally, anti-acetylcholinesterase and antispasmodic (isolated rat distal colon) activities, general acute toxicity (*Artemia salina* model), and immunomodulatory properties (cytotoxicity on isolated mouse macrophages) were studied. Detailed analyses of the essential oil led to the identification of 3-methoxycuminyl 2-methylbutanoate (a new natural product) and 3-methoxycuminyl 3-methylbutanoate (a rare natural product). The obtained esters and intermediates in the synthesis of the starting alcohol (3-methoxycuminol) were subjected to a battery of 1D- and 2D-NMR experiments. The synthesized esters were additionally characterized by GC–MS, IR, and UV–Vis. The synthesized compounds (ten in total) were biologically tested in the same way as the extracted *P. dysenterica* essential oil. The obtained low acute toxicity and promising antimicrobial potential suggest that the *P. dysenterica* essential oil might partially explain the ethnopharmacological application of *P. dysenterica* plant material for the treatment of gastrointestinal infections.

## 1. Introduction

Medicinal plants as industrial crops represent a renewable source of pharmaceuticals, essential oils, biocides, etc. [1]. Besides protecting plant biodiversity, the cultivation of new, potentially interesting medicinal plants is a way to strengthen local agro-economics. The choice of new medicinal plant crops could be based on ethnopharmacological knowledge, such as in the successful example of *Artemisia annua* [2]. Most medicinal plant species enlisted in modern pharmacopoeias have found their way to cultivation fields; however, numerous ethnopharmacologically renowned taxa have their use ceased over time due to various reasons, such as erroneous attributions of beneficial effects, toxicity, or being simply forgotten. *Pulicaria dysenterica* (L.) Bernh. (syn. *Inula dysenterica*, eng. great fleabane; family Asteraceae (Compositae)), native to Europe and Western Asia [3], represents an excellent example of an underused and almost abandoned folk remedy.

The name of the species in Latin, *dysenterica*, refers to the supposed property of this plant taxon to cure dysentery, which was the motivation for Carl Linnaeus to include it in his *Flora Svecica* [4]. The bruised leaves emit a characteristic smell, and they were used in medieval times to repel fleas and other insects. Additionally, the leaves were burned and the smoke was used as a domestic pesticide, hence the common name fleabane [5]. The use of *P. dysenterica* was mentioned many times in the works of later authors, such as the famous English herbalist Nicholas Culpeper. Thus, *P. dysenterica* could simultaneously provide access to specialty materials such as insecticides or an application in treating (infectious) gastrointestinal disorders. A possible common link between the two diametrical applications could be the anti-acetylcholinesterase activity of the constituents of *P. dysenterica* [6].

Up to now, the volatile secondary metabolites of *P. dysenterica* have only been the subject of a few studies. The essential oil was only investigated on two previous occasions [3,7], the latest of which was published 10 years ago [3]. This prompted us to re-analyze the composition of the essential oil from the aerial parts with the aim of finding the compounds responsible for the mentioned activities. The analysis required the synthesis of certain major and minor constituents, which enabled the testing of pure compounds and the essential oil for biological/pharmacological properties relevant to ethnopharmacological use. These included antimicrobial, antispasmodic (isolated rat distal colon), and anti-acetylcholinesterase activities, in addition to assessing the general acute toxicity (*Artemia salina* model) and cytotoxicity on isolated rat macrophages. Hence, in this work, we put forward various chemical and biological/pharmacological data needed to assess *P. dysenterica* as a potential plant crop.

## 2. Results

### 2.1. Composition of P. dysenterica Essential Oils and Their Variability

The aerial parts of *P. dysenterica* yielded yellowish essential oils (0.12–0.13%, *w*/*w*) with a pleasantly sweet odor. GC–MS, UV, IR, and NMR analyses, chromatographic separation, and synthetic work allowed for the identification of 296 constituents of the essential oils from dry *P. dysenterica* aerial parts (Table 1). The identified constituents represented 94.7–96.6% of the total essential oils, with oxygenated mono- and sesquiterpenoids (55.2–68.0% and 12.7–19.5%, respectively) as the most abundant compound classes. Among them, neryl isobutyrate and 3-methoxycuminyl isobutyrate represented major essential-oil constituents (16.4–22.1% and 25.5–31.1%, respectively). Only quantitative differences were noted between the essential oils collected from different *P. dysenterica* populations. Contrary to this slight quantitative compositional dissimilarity between the analyzed samples, the herein presented composition (Table 1) was very different from those that Basta et al. [7] and Mumivand et al. [3] published.

### 2.2. Identification, Synthesis, and NMR Spectral Characterization of the (New) 3-Methoxycuminyl Esters from P. dysenterica Essential Oil

**Figure 1:**
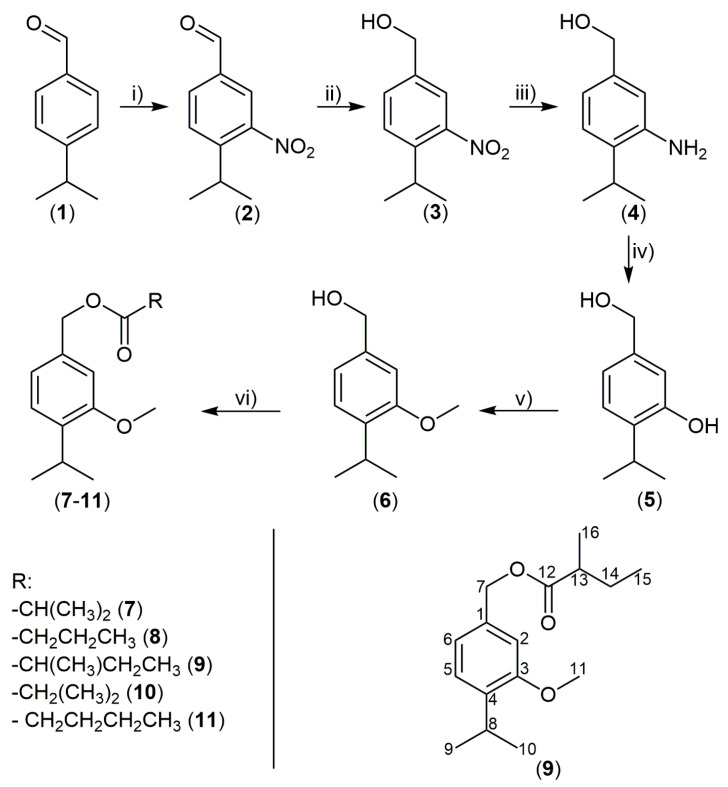
Synthesis of 3-methoxycuminyl esters: (i) HNO_3_ and H_2_SO_4_; (ii) NaBH_4_ and MeOH/THF; (iii) H_2_, Pd/C, and EtOAc; (iv) H_2_SO_4_, H_2_O, and NaNO_2_; (v) MeI, K_2_CO_3_, and CH_3_COCH_3_; (vi) RCOOH, DCC/DMAP, and CH_2_Cl_2_.

One of the major essential-oil constituents (3-methoxycuminyl isobutyrate) was tentatively easily identified based solely on the matching of the corresponding retention indices and mass spectra with literature data [10]. Additionally, partial ion current (PIC, *m*/*z* 137, 163, and 180 ions) chromatograms of the essential-oil samples indicated the presence of additional constituents related to 3-methoxycuminyl isobutyrate, i.e., most probably other esters of 3-methoxycuminol. After a detailed consideration of the mass spectra and the GC retention data of these essential-oil constituents, we could tentatively identify them as 3-methoxycuminyl esters of 2-methylbutanoic and 3-methylbutanoic (isovaleric) acids. The specific 3-methoxycuminol, needed to prepare the synthetic samples of esters for a direct comparison, was commercially unavailable. For that reason, we followed an approach that included two parts: the synthesis 3-methoxycuminol and the preparation of a small synthetic library of five esters (3-methoxycuminyl 2-methylpropanoate, butanoate, 2-methylbutanoate, 3-methylbutanoate, and pentanoate) starting from 3-methoxycuminol and the appropriate acids via the Steglich procedure (Figure 1).

Co-injection experiments confirmed the mentioned tentative identifications, i.e., the essential oil contained the following esters of 3-methoxycuminol: 2-methylpropanoate (isobutyrate), 2-methylbutanoate, and 3-methylbutanoate (isovalerate). One of the synthesized esters (2-methylbutanoate), according to a detailed literature search, is a new natural product previously undescribed or mentioned in the literature so far. In contrast, the identified 3-methylbutanoate is a rare natural product that was only identified as a constituent of the *Inula viscosa* essential oil [11]. Additionally, the synthesized 3-methoxycuminyl butanoate and pentanoate are new compounds. A literature search showed that 3-methoxycuminyl esters are rare secondary metabolites in the plant kingdom. According to a SciFinder search of the Chemical Abstracts Service (CAS) database, at the time of the investigation, only 16 reports have dealt with 3-methoxycuminyl esters (2 with 3-methoxycuminyl acetate, 13 with the isobutyrate, and only 1 with the isovalerate). The mentioned literature search showed that their occurrence in nature is restricted to Asteraceae and seems typical for the tribes of Inuleae (genera *Inula* and *Pulicaria*) and Senecioneae (genus *Doronicum*).

The obtained esters and intermediates in the synthesis of the starting alcohol (3-methoxycuminol) were subjected to a battery of 1D- (^1^H and ^13^C, including ^1^H spectra with homonuclear and ^13^C spectra without heteronuclear decoupling, as well as DEPT90 and DEPT135) and 2D- (gradient NOESY, HSQC, and HMBC) NMR experiments, as well as MS, IR, and UV–Vis measurements. The spectral data and assignments are summarized in Table 2, the experimental section, and (Supplementary Materials Figures S1–S33); a numbering scheme of C atoms is given in Figure 1. Additionally, in the case of 3-methoxycuminol (**6**), a pivotal point in the structural elucidation was the complete spin analyses, i.e., ^1^H NMR simulation which was conducted as recently published by Radulović et al. [12]. Combining data from these spectra allowed for the assignation of all ^1^H and ^13^C NMR signals. The assignment of signals is later discussed in detail for the new natural product—3-methoxycuminyl 2-methylbutanoate (**9**). In the case of all other compounds, the assignment was analogous.

The ^1^H and ^13^C NMR spectra of compound **9** (Supplementary Materials Figures S14 and S15) contained the expected number of signals. A doublet at 1.20 ppm (*J* = 6.9 Hz, 6 H) was assigned to the two methyl groups from the isopropyl fragment (C-9 and C-10 protons). These protons were coupled with a one-proton septuplet at 3.30 ppm. The HSQC spectrum (Supplementary Materials Figure S20) enabled the assignation of ^13^C NMR signals of the carbon atoms from the same structural fragment (C-8–26.6 ppm, and C-9 and C-10–22.6 ppm). The HMBC spectrum (Supplementary Materials Figure S21) showed a correlation between C-8 proton from the isopropyl moiety and four ^13^C NMR signals. According to DEPT90 and DEPT135 (Supplementary Materials Figures S17 and S18), these were: two non-protonated carbon atoms at 137.0 and 156.8 ppm, two methyl carbon atoms at 22.6 ppm, and one methine carbon atom at 126.1 ppm, which were assigned to C-4, C-3, C-9, C-10, and C-5, respectively. Additionally, besides signals for C-3, C-4, and C-8 carbon atoms, the C-5 proton at 7.19 ppm (d, *J* = 7.7 Hz, 1H) displayed long-range coupling to carbon atoms at 134.7, 110.1, 120.3, and 66.1 ppm that were assigned to C-1, C-2, C-6, and C-7, respectively. In the case of the methoxy group, the protons appeared as a singlet at 3.83 ppm that was directly connected (according to the HSQC spectrum) to the carbon atom at 55.3 ppm. As in our previous assignations of 2-methylbutyrates [13], a methyl group carbon atom signal at 11.6 ppm was linked to protons at 0.91 (t, *J* = 7.4 Hz), and the carbon atom from another methyl group at 16.6 ppm was linked to protons at 1.17 (d, *J* = 7.0 Hz). Based on the HMBC correlations of the protons of these two methyl groups (C-15 and C-16), as well as data from HSQC, DEPT90, and DEPT135, the resonance at 2.43 ppm was assigned to C-13 protons and the resonances at 1.71 and 1.49 ppm were assigned to the two diastereotopic C-14 protons.

### 2.3. Biological Activity

The primary goal of this study was to provide data on the possible biological activity (AChE (acetylcholinesterase) inhibitory, antimicrobial, antispasmodic, and cytotoxicity activities) of the *P. dysenterica* essential oil (**EO**) and the main and new **EO** constituents, as well as to assess the safety of the **EO** and selected synthesized compounds by screening for acute toxicity in the model of *Artemia salina*. Alongside the isomeric 3-methoxycuminyl butanoates and pentanoates from the library, 3-nitrocuminaldehyde (**2**), 3-nitrocuminol (**3**), 3-aminocuminol (**4**), 3-hydroxycuminol (**5**), and 3-methoxycuminol (**6**) were also assayed in the mentioned biological tests (we were motivated to include these compounds in the assays because the presence of the phenolic hydroxyl, amino, or nitro group might significantly alter the activity of the natural compounds). These compounds (**2–5**; Figure 1) were intermediary products of the reaction sequences in synthesizing the starting alcohol (**6**).

#### 2.3.1. AChE Inhibitory Activity

Recent studies showed that volatile natural products from various essential oils could be used as alternatives to synthetic insecticides against stored-product pests and insects in general [14]. The potential AChE inhibitory activity of the herein studied essential oil or some of the synthesized compounds (easily, rapidly, and cheaply available even on a large scale) could have enormous industrial value in the constant quest for safe insecticides. *P. dysenterica* essential oil, cuminal (**1**), and a spectrum of the multi-functionalized synthesized compounds (**2–11**) allowed for the systematic evaluation of their AChE inhibitory activity. The results of the AChE inhibition assays are summarized in Table 3. Due to solubility issues, the highest tested concentration providing reliable results was 125 mg/L for the **EO** or 500 µmol/L for compounds **1–11** (the final concentration in the wells).

As expected, among the tested compounds, the esters had the lowest inhibitory activity, which was lower than 5%. A low inhibitory activity was also noted for cuminal and the **EO** (12.8 and 14.9%, respectively). Interestingly, the presence of a nitro group was found to be necessary for this type of activity. The synthesized 3-nitrocuminaldehyde (**2**) and 3-nitrocuminol (**3**) displayed much greater AChE inhibitory activity compared with cuminal (**1**), whereas the reduction of the nitro group to the amino one drastically reduced the inhibitory effect (Table 3). Inhibitors of acetylcholinesterase are occasionally applied to treat some digestive problems [15]. As mentioned before, infusions of *P. dysenterica* are used for a similar purpose [4], but it appears that such activity does not come from the plant’s essential oil.

#### 2.3.2. Brine Shrimp Lethality

The acute toxicity of the **EO** and the selected synthesized compounds was tested with an *A. salina* acute toxicity assay, as described previously by Radulović and coworkers [16]. The following compounds were chosen to be tested: compounds **7**, **8**, and **10** (constituents of the **EO**); 3-hydroxycuminol (**5**); and 3-methoxycuminol (**6**). Compounds **5** and **6**, intermediary products of the reaction sequences depicted in Figure 1, could be potential essential-oil or plant constituents (e.g., compound **5** was already found as the constituent of the extracts of *Eupatorium fortune* [17]).

When applied at 3.9–125 mg/L, the tested samples showed a low to moderate toxicity compared with the positive control (the obtained LC_50_ values for SDS were comparable to literature values [18]). The synthesized alcohols (**5** and **6**) showed a low toxicity in the *A. salina* acute toxicity assay. Mortality for the highest tested concentrations of compound **5** after 24 h was only 20%, whereas the LC_50_ after 48 h was 125 mg/L (0.75 mM). In the case of compound **6**, LC_50_ values were 92.2 mg/L (0.51 mM) and 65.6 mg/L (0.36 mM) after 24 and 48 h, respectively. It seems that the oxygenation in position 3 of the aromatic ring (i.e., the presence of a hydroxy or methoxy group in compounds **5** and **6**, respectively) is important for toxicity. It is interesting to note that compound **6** showed a higher toxicity than **5**, i.e., the methylation of the phenol group raised toxicity against *A. salina*, probably due to the changes in the polarity of the mentioned compounds. In the case of the tested esters (**7**, **9**, and **10**), the mortality of the nauplii of compounds **7–11** was up to 40% after 24 h (for this reason, we could not calculate LC_50_ with an acceptable degree of confidence). After 48 h, it was possible to calculate LC_50_ values of 0.66, 0.28, and 0.35 mM for compounds **7**, **9**, and **10**, respectively. Interestingly, the **EO** turned out to be non-toxic to *Artemia salina* (the mortality for the highest tested concentrations of the **EO** was less than 5%, as in the case of the negative control [18]).

#### 2.3.3. Antimicrobial Activity

The antimicrobial testing of the synthesized compounds showed prominent activity against all tested groups of microorganisms; the active concentrations ranged from 0.01 to 4.00 mg/mL (0.06-15.15 µmol/mL; see Table 4). The only exceptions where activity was not observed in the tested concentration range were compounds **10** (against *S. aureus*) and **11** (against *S. epidermidis*). It is notable that together with the **EO**, intermediate compounds **2–6** showed significantly higher antimicrobial potential than esters **7–11**, which constituted the **EO** (**7**, **9,** and **10**), and their homologs (**8** and **11**). Considering the overall activity, the highest potency was observed for compounds **3** and **6**, with average MIC values of 425 and 343 mg/L, respectively. In addition, interesting findings were observed regarding selectivity, where the **EO**, the intermediate 3-nitrocuminaldehyde (**2**), and alcohols (**4** and **5**) exhibited significantly higher potency against Gram-positive strains, which was not the case with compounds **3** and **6**, where higher activity was observed against fungal strains. The same higher antifungal potency was noted in the case of all esters (**7–11**). This pattern of activity was prominent in the case of compound **10**; a four times lower concentration inhibited fungal growth in comparison with those needed for bacterial growth inhibition. Among the bacterial strains, *K. rhizophila* and *A. baumanii* were the most sensitive ones, while *P. aeruginosa* and *E. coli* showed the highest resistance. As expected, the yeast showed the higher sensitivity to the two tested fungal strains.

*Salmonella* isolates were sensitive to the tested samples at concentrations in the range of 0.12–4.00 g/L (0.72–15.15 mmol/L; see Table 5), which was similar to that obtained for the reference strain of the same bacterial species (0.50–4.00 g/L). Notably, some of the isolates showed a slightly higher sensitivity than the ATCC strain, but regarding the testing of the activity of the tested compounds showed a very similar level of antimicrobial potency as against ATCC strains. Once again, a higher antimicrobial power was exhibited by the **EO** and **2–6**, among which compound **4** showed the least antimicrobial effect, which is the same pattern as the one noted for the tested bacterial and fungal (ATCC) species. The most active compound in general, **5**, also showed the highest activity against *Salmonella* isolates. In the case of compounds **7**–**11**, which once again exhibited a significantly lower antimicrobial activity, compound **8** showed the highest anti-salmonella effect. According to these results, the application of the *P. dysenterica* **EO** might contribute to the curing of gastrointestinal infectious diseases owing to its antimicrobial action. However, it should be used with caution due to relatively high active concentrations and the observed activity against all tested microorganisms, which might influence the existence and/or recovery of commensal intestinal microbial flora.

Previous studies on *P. dysenterica* antimicrobial activity are scarce and only investigated aerial part extracts [19,20]. These studies showed the antimicrobial effect of an aqueous extract against *Bacillus cereus* and *Vibrio cholerae* and a methanol extract against *S. aureus*, *V. cholerae,* and *B. cereus*, and a chloroformic extract was found to be active against *S. aureus* and *V. cholerae*. However, the mentioned extracts were not chemically characterized in these two studies, so the herein tested essential oil activity cannot be compared to these results, especially considering the additional differences in the methods for the determination of antimicrobial activity (disc diffusion vs. microdilution). In another study, a high inhibitory potential of a fraction rich in 3-methoxycuminyl isobutyrate (40%) was observed, as a microbicidal effect at 0.025 mL/L against *Helicobacter pylory* [21] was demonstrated. Herein, the same compound in its pure state showed a weaker antimicrobial potential against the tested Gram-negative strains. These observed differences in the activities are probably related to the variability in the sensitivity of the bacterial species, as well as to the combined effect of 3-methoxycuminyl isobutyrate with other compounds present in the fraction tested in the mentioned study. Notably, the **EO** in the present study was found to possess a higher antimicrobial potential than the activities observed for the pure major compounds. This confirms that some other compounds, present at a lower percentages, significantly contributed to the observed effect of the **EO**. Some of them, such as nerol, (*E*)-caryophyllene, neryl isobutyrate, neryl isovalerate, and caryophyllene oxide, presented in a relatively high percentage (1.4–22.1%) in the herein studied **EO**, and others are antimicrobial agents, as confirmed by many studies [22,23,24,25,26,27].

#### 2.3.4. Antispasmodic Activity

Different concentrations of the pooled **EO** sample, alongside papaverine as the positive control, were assayed for their effect on spontaneous contractions of the isolated rat distal colon. The negative control (diluted DMSO, 0.5%, *v*/*v*) did not affect spontaneous distal colon contractions. In contrast, the positive control, papaverine, exhibited gastrointestinal smooth muscle relaxation, with an EC_50_ value of 3.7 µM; it did not affect the frequency of contractions in the tested concentration range. The tested concentrations of the **EO** ranged from 0.025 mg/L to 0.25 g/L (the final concentration in the 20 mL tissue bath containing Tyrode’s solution). Higher concentrations of the **EO** were not tested due to the low solubility of the **EO** in Tyrode’s solution. Unexpectedly, monitoring distal colon contraction showed that the **EO** did not affect them. Even in the highest tested concentration, 0.25 g/L, the amplitude of distal colon contractions or the number of contractions per minute remained similar to those from the negative control. The antispasmodic potential of the **EO** would be an important aspect of this essential oil since the ethnopharmacologically suggested application involves alleviating symptoms from the hyperfunction of the colon, i.e., diarrhea [28]. The obtained results indicate that the **EO** did not exert any significant action on the isolated rat distal colon contractions, which is why we did not pursue the potential action of the synthetized compounds. It is worth mentioning that this is the first study to evaluate the antispasmodic action of the essential oil arriving from plants belonging to the *Pulicaria* genus. Different *Pulicaria* species, e.g., *P*. *glutinosa*, have been traditionally used by the United Arab Emirates population for treating different gastrointestinal disorders, including colitis and helminthiasis [29]. Leaf water extracts of *P*. *glutinosa* were found to modulate the spontaneous contractions of isolated rabbit jejunum, where an initial stimulation of contractions was seen in lower doses and higher doses caused an inhibition of contractions, reaching an IC_50_ of 2.3 mg/mL [29].

#### 2.3.5. Cytotoxicity of EO and Pure Compounds

The essential oil of *P*. *vulgaris* was evaluated for its cytotoxicity toward breast and liver cancer cells, and it was shown to exert IC_50_ values ranging from 5 to 7 mg/L [30]. In contrast, for the oils of *P*. *crispa*, *P*. *undulata,* and *P*. *incisa*, a slightly less cytotoxic potential towards the same cancer cell lines was previously demonstrated [30]. The **EO** used in our experiments showed much lower cytotoxic potential, and a concentration of 100 mg/L reduced the viability of peritoneal macrophages by more than 50% (Table 6). In the following dilution (10 μg/mL), the toxicity was significantly reduced and the viability of the cells was comparable to that of RPMI-treated cells. This activity could have potentially arisen from a different composition of the **EO** sample at hand, as well as the higher resistance of normal cells isolated from healthy animals or the selectivity of this oil towards cancerous cells. The mentioned activity of the *P*. *vulgaris* essential oil was suggested to be arriving from carvotanacetone, thymol, and thymyl isobutyrate, which the oil possesses in abundance [31]; in comparison, the herein tested sample of **EO** possesses neryl isobutyrate and 3-methoxycuminyl isobutyrate as its major essential-oil constituents. On previous occasions, a plant extract of *P*. *undulata* and pure flavonoids isolated from it showed promising cytotoxic potential toward breast and liver cancer cells [32]. Similar results were found for *P*. *orientalis* ethanolic extracts, which showed significant cytotoxic potential in a culture of human amniotic epithelial cells with an IC_50_ value of 18 mg/L [33]. Some specific mechanisms of action of axillarin, isolated from *P*. *crispa* extract, suggest that it may serve as a potential agent in fighting cancers [34].

Besides the **EO**, the highest cytotoxic activity towards rat peritoneal macrophages in this study was exerted by compounds **2** and **5** in their highest concentrations (Table 6), while **6**, **9,** and **10** exerted moderate cytotoxic potential at the same concentrations. All other tested concentrations of the **EO** and the mentioned compounds did not show any cytotoxic potential, nor did **3**, **4,** and **7** in any of the applied concentrations (Table 6). Interestingly, the mutual presence of nitro and aldehyde groups in compound **2** was important for this activity. The synthesized 3-nitrocuminaldehyde (**2**) displayed a much greater activity than 3-nitrocuminol (**3**), and the change of the nitro group to the phenol group drastically magnified cytotoxicity (Table 6). It seems that the presence of a hydroxy group or a methoxy group in position 3 in compounds **5** and **6**, respectively, is of importance for cytotoxicity, whereas the esterification of the phenol group ultimately reduces the mentioned activity (Table 6). The difference in the cytotoxic activity of the **EO** and synthesized natural products (**7**, **9**, and **10**) suggested that other identified essential-oil constituents, or potential synergistic effects of present plant metabolites, were responsible for the obtained cytotoxic activity towards rat peritoneal macrophages.

The observed relationship between the cytotoxic potential of the **EO** and synthesized compounds, as well as their correspondent MICs, can be rationalized/systematized in several possible ways. Firstly, the **EO** is an at least 10-fold more potent antimicrobial agent (Table 4 and Table 5) than it is a cytotoxic agent (Table 6), indicating that it might be adequate for application in the treatment of infectious diseases since there is a possible pharmacological window that does not overlap with its toxicity profile. This is especially true for the **EO** concentrations that exerted no notable toxicity towards macrophages at near-MIC values (Table 6). Secondly, compounds exerting the highest cytotoxic potential (**2** and **5**) at concentrations of 10^−4^ M (Table 6) exhibited antimicrobial potential in a close concentration range (MIC 0.01–3 μM) towards the majority of the tested microorganisms, with only a few outliers where the MIC was 100x higher (*P. aeruginosa* and *A. brasiliensis*; Table 4). These results indicate that, when applied, these compounds might act not only as antimicrobials but also as cytotoxic agents against immune system cells. Compounds with a moderate toxicity could include compounds **6**, **9**, and **10** that, at the highest tested concentration, decreased cell viability from around 20 to 30% (Table 6). These compounds also exerted modest antimicrobial activity, with MIC values of between 3 and 20 μM (Table 4 and Table 5). Finally, compounds with no notable cytotoxic potential towards macrophages at the highest tested concentration (compounds **3**, **4,** and **7**) exhibited a weak cytotoxic potential, except for compound **3** (Table 4 and Table 5). These data suggest that the antimicrobial activity of the **EO** might not be directly associated with the activity of a single compound, but rather a synergistic action of compounds within. This issue remains to be clarified in future studies.

## 3. Materials and Methods

### 3.1. General

All used solvents (HPLC grade) and chemicals were purchased from Sigma-Aldrich (St. Louis, MO, USA), Merck (Darmstadt, Germany), or Carl Roth (Karlsruhe, Germany). All chemicals used in the bioassays were of the highest available grade (Sigma-Aldrich, Merck, TCI Co, Tokyo, Japan; Acros Organics, Morris Plains, NJ, USA; AppliChem, Darmstadt, Germany; Santa Cruz Biotechnology, Dallas, TX, USA; and Teva, Belgrade, Serbia). Silica gel 60, particle size distribution of 40–63 mm (Acros Organics, Geel, Belgium), was used for dry-flash chromatography, whereas precoated Al silica gel plates (Kieselgel 60 F_254_, 0.2 mm, Merck, Darmstadt, Germany) were used for analytical TLC analyses. The spots on TLC were initially visualized with UV light (254 nm), followed by spraying with 50% (*v*/*v*) aq. H_2_SO_4_ followed by heating. ATR-IR measurements (attenuated total reflectance) were carried out using a Thermo Nicolet model 6700 FTIR instrument (Waltham, MA, USA). UV spectra (in acetonitrile) were measured using a UV-1800 PC Shimadzu spectrophotometer (Tokyo, Japan). ^1^H, ^13^C NMR, and two-dimensional spectra were recorded on a Bruker Avance III 400 MHz NMR spectrometer (^1^H at 400 MHz and ^13^C at 100.6 MHz) using the built-in Bruker pulse sequences (Fällanden, Switzerland). All NMR spectra were measured at 25 °C in deuterated chloroform with tetramethylsilane as the internal standard. Chemical shifts are reported in ppm (δ) and referenced to tetramethylsilane (δ_H_ 0) in ^1^H NMR spectra or residual CHCl_3_ (δ_H_ 7.26) and ^13^CDCl_3_ (δ_C_ 77.16) in heteronuclear 2D spectra. The following abbreviations were used to designate multiplicities: *br*, broad signal; *s*, singlet; *d*, doublet; *t*, triplet; *q*, quartet; *sext*, sextet; *sept*, septet; *dd*, doublet of doublets; *dquint*, doublet of quintets; *dtd*, doublet of triplets of doublets; *ddtd*, doublet of doublets of triplets of doublets; *dqd*, doublet of quartets of doublets; *septddd*, septet of doublets of doublets of doublets; and *tsept*, triplet of septets. In the case of complex signals (overlapped or higher order), δ_H_ and *J* values were manually adjusted to fit the experimentally available values and further optimized using MestreNova software (tools/spin simulation) [12]. Elemental analysis (microanalysis of carbon, hydrogen, and oxygen) was carried out with a Carlo Erba Elemental Analyzer model 1106 (Carlo Erba Strumentazione, Milan, Italy). The GC–MS analyses (three repetitions) were carried out using a Hewlett-Packard 6890N gas chromatograph equipped with a fused silica capillary column DB-5MS (5% diphenylpolysiloxane, 95% dimethylpolysiloxane, 30 m × 0.25 mm, film thickness of 0.25 µm, Agilent Technologies, Lexington, USA) and coupled with a 5975B mass selective detector from the same company. The injector and interface were operated at 250 °C and 320 °C, respectively. The oven temperature was raised from 70 to 300 °C at a heating rate of 5 °C/min; the heating program ended with an isothermal period of 10 min. As a carrier gas, helium at 1.0 mL/min was used. The samples were injected in a split mode (injection volume was 1 µL; split ratio was 40:1). MS conditions were as follows: ionization voltage of 70 eV, acquisition mass range of 35–650, and scan time of 0.32 s. Essential-oil constituents were identified by comparisons of their GC retention indices (relative to C_7–_C_31_ *n*-alkanes on the DB-5MS column [35]) with literature values [8] and their mass spectra with those of authentic standards and values from Wiley 11, NIST17 [9], MassFinder 2.3, and a homemade MS library with the spectra corresponding to pure substances. Wherever possible, constituents were also identified by co-injection with an authentic sample. The GC–FID analyses (three repetitions of each sample) were carried out using an Agilent 7890A GC system equipped with a single injector, one flame ionization detector (FID), and a fused silica capillary column HP-5MS (5% phenylmethylsiloxane, 30 m × 0.32 mm, film thickness of 0.25 μm, Agilent Technologies, Palo Alto, CA, USA). The oven temperature was programmed from 70 °C to 300 °C at 15 °C/min and then isothermally held at 300 °C for 5 min; the carrier gas was nitrogen at 3.0 mL/min; the injector temperature was held at 250 °C. The samples, comprising 1.0 μL of corresponding solutions, were injected in a splitless mode. The parameters of the FID detector were as follows: heater temperature of 300 °C, H2 flow of 30 mL/min, air flow of 400 mL/min, makeup flow of 23.5 mL/min, and data collection with an Agilent GC Chemstation with a digitization rate of 20 Hz. The GC–FID quantification of 3-methoxycuminyl isobutyrate, 2-methylbutanoate, and isovalerate was carried out by constructing calibration curves, compound concentration versus peak area (C = f (A)), for twelve dilutions (12.8, 6.4, 3.2, 1.6, 0.8, 0.4, 0.2, 0.1, 0.05, 0.025, 0.0125, and 0.00625 mg/mL) of the standards dissolved in ethyl acetate. Each sample was analyzed for three consecutive runs. The quantification of other identified essential-oil components was carried out using peak-area normalization with response factors from the literature [36,37,38,39]. Experimentally obtained values of response factors for representatives of all groups of essential-oil constituents were in good agreement with those reported in previous reports [36,37,38,39]. Nonane was used as the internal standard for these analyses.

### 3.2. Plant Material

Flowering aerial parts of *Pulicaria dysenterica* were collected from two wild-growing populations: one from the village Skrapež (near Leskovac, Serbia, 450 m above sea level, 42°99′34″ N and 22°09′67″ E; sample (**A**) and another from urban settings of the city of Niš (43°32′06” N and 21°94′28” E, at an altitude of 195 m; sample (**B**) in August 2012 and 2010, respectively. Voucher specimens were deposited in the Herbarium of the Faculty of Sciences and Mathematics, University of Niš, Serbia, under the acquisition numbers MM0902 and MM0893, respectively. The identity of the plant material was confirmed by a trained botanist, the custodian of the mentioned herbarium.

### 3.3. Hydrodistillation

The dry aerial parts (two times three batches, ca. 200 g each) were submitted to hydrodistillation with 2.0 L of distilled water for 2.5 h, and a Clevenger-type apparatus was used to produce yellowish essential oils. The obtained essential oils were separated by extraction with diethyl ether and dried with anhydrous magnesium sulphate; the solvent was evaporated under a gentle stream of nitrogen at room temperature, and the essential oils were then immediately analyzed by GC–MS.

### 3.4. Synthesis of 3-Methoxycuminol

#### 3.4.1. Nitration of Cuminaldehyde

Nitration was accomplished following a method by Atkinson and Simpson [40]. A mixture of concentrated nitric (46 mL) and sulfuric acids (52 mL) was cooled to 0 °C and stirred. Then, cuminaldehyde (**1**; 10 g, 67.57 mmol) was dropwise added to this solution (temperature control in the interval of 0–5 °C). The mixture was stirred for 30 min. Then the cooling bath was removed and the mixture was stirred for another 30 min. The reaction mixture was quenched with excess ice-water, and the product was taken up by diethyl ether (4 × 150 mL). The organic layers were combined, dried with anhydrous MgSO_4_, and concentrated under reduced pressure. Crude 4-isopropyl-3-nitrobenzaldehyde (**2**; 3-nitrocuminaldehyde) was purified by dry-flash column chromatography on silica gel using *n*-hexane/Et_2_O mixtures of increasing polarity as the eluents. The purity of 3-nitrocuminaldehyde (**2**) was checked by TLC, GC–MS, and NMR. The yield of 3-nitrocuminaldehyde (**2**; 12.26 g (63.52 mmol)) was 94%. The spectral data of **2** are given below:

*4-Isopropyl-3-nitrobenzaldehyde (**2**; 3-nitrocuminaldehyde)*: retention index (RI) = 1533 (DB-5MS column); UV (CH_3_CN) λ_max_(log ε) 241 (4.16), 199 (4.13) nm; FTIR (neat; cm^−1^) 2971, 2873, 1700, 1613, 1568, 1527, 1461, 1388, 1354, 1297, 1214, 1192, 1131, 1053, 1008, 948, 924, 903, 838, 819, 767, 739, 704, 671, 624; MS (EI), *m*/*z* (%) 193(1) [M^+^], 192(2), 179(3), 178(23), 177(5), 176(48), 162(4), 160(8), 158(13), 151(10), 150(4), 149(25), 148(68), 147(15), 146(9), 145(8), 136(4), 135(39), 134(14), 133(39), 132(32), 131(15), 130(24), 121(8), 120(14), 119(11), 118(12), 117(19), 116(19), 115(77), 108(4), 107(36), 106(19), 105(21), 104(17), 103(45), 102(19), 101(4), 95(7), 94(11), 93(10), 92(14), 91(100), 90(8), 89(15), 87(4), 79(13), 78(21), 77(92), 76(14), 75(18), 74(14), 65(26), 64(5), 63(22), 62(8), 59(7), 53(6), 52(7), 51(31), 50(14), 43(48), 41(16), 39(20); analyzed C 62.20, H 5.73, N 7.23, O 24.84%, calculated for C_10_H_11_NO_3_, C 62.17, H 5.74, N 7.25, O 24.84%; ^1^H NMR (CDCl_3_) δ 1.35 (*d*, *J* = 6.8 Hz, 6H, CH_3_-9 and CH_3_-10), 3.47 (*sept*, *J* = 6.8 Hz, 1H, CH-8), 7.70 (*d*, *J* = 8.1 Hz, 1H, CH-5), 8.07 (*dd*, *J* = 8.1, 1.5 Hz, 1H, CH-6), 8.20 (*d*, *J* = 1.5 Hz, 1H, CH-2), 10.04 (*s*, 1H, CH-7); ^13^C NMR (CDCl_3_) δ 23.4 (C-9, and C-10), 29.1 (C-8), 125.1 (C-2), 128.7 (C-5), 132.4 (C-6), 134.9 (C-1), 148.9 (C-4), 150.1 (C-3), 189.6 (C-7).

#### 3.4.2. Synthesis of 3-Nitrocuminol

A mixture of 3-nitrocuminaldehyde (**2**; 12 g, 62.18 mmol) and sodium borohydride (4.73 g, 125 mmol) in an anhydrous methanol/tetrahydrofuran mixture (75 mL; 1:9, *v*/*v*) was stirred at 0 °C for 30 min and additional 2 h at room temperature. A saturated solution of NaHCO_3_ (100 mL) was added, and the mixture was stirred for 10 min. The reaction mixture was extracted with diethyl ether (4 × 100 mL), followed by a usual work-up (drying with MgSO_4_ and solvent evaporation), and it yielded 11.88 g (60.93 mmol) of the pure 3-nitrocuminol (**3**; the purity of the product was checked by TLC, GC–MS, and NMR). The yield of 3-nitrocuminol (**3**) was 98%. The spectral data of **3** are given below:

*(4-Isopropyl-3-nitrophenyl)methanol (**3**; 3-nitrocuminol)*: retention index (RI) = 1685 (DB-5MS column); UV (CH_3_CN) λ_max_(log ε) 292 (3.53), 242 (3.99), 206 (4.47) nm; FTIR (neat; cm^−1^) 3338, 2968, 2872, 1622, 1567, 1523, 1463, 1386, 1352, 1202, 1135, 1104, 1054, 887, 833, 808, 765, 675; MS (EI), *m*/*z* (%) 195(2) [M^+^], 194(1), 180(10), 179(5), 178(65), 160(21), 153(3), 152(5), 150(26), 149(8), 148(43), 144(4), 137(17), 136(9), 135(13), 134(28), 133(15), 132(7), 130(22), 128(9), 121(8), 120(12), 118(12), 117(35), 116(15), 115(56), 109(4), 108(6), 107(43), 106(27), 105(27), 104(4), 103(28), 102(10), 94(11), 93(14), 92(14), 91(98), 90(11), 89(25), 87(4), 79(56), 78(25), 77(100), 76(6), 75(4), 74(5), 65(20), 64(5), 63(19), 62(6), 59(4), 57(7), 55(9), 53(13), 52(9), 51(28), 50(9), 44(5), 43(52), 41(22), 39(24); analyzed C 61.55, H 6.70, N 7.20, O 24.55%, calculated for C_10_H_13_NO_3_, C 61.53, H 6.71, N 7.18, O 24.58%; ^1^H NMR (CDCl_3_) δ 1.29 (*d*, *J* = 6.8 Hz, 6H, CH_3_-9 and CH_3_-10), 3.39 (*sept*, *J* = 6.8 Hz, 1H, CH-8), 4.33 (*br s*, 1H, OH), 4.73 (*br s*, 2H, CH_2_-7), 7.46 (*d*, *J* = 8.1 Hz, 1H, CH-5), 7.53 (*dd*, *J* = 8.1, 1.5 Hz, 1H, CH-6), 7.70 (*d*, *J* = 1.5 Hz, 1H, CH-2); ^13^C NMR (CDCl_3_) δ 23.6 (C-9, and C-10), 28.4 (C-8), 63.8 (C-7), 121.9 (C-2), 127.8 (C-5), 130.7 (C-6), 139.8 (C-1), 141.6 (C-4), 149.7 (C-3).

#### 3.4.3. Reduction of 3-Nitrocuminol

A solution of 3-nitrocuminol (**3**; 11.5 g, 58.97 mmol) and 5% Pd/C (1 g) in anhydrous ethyl acetate (50 mL) was stirred under hydrogen (atmospheric pressure) at room temperature for a duration of 6 h. After the completion of the reaction (monitored by TLC and GC–MS), the mixture was filtered and concentrated under reduced pressure. Crude 3-aminocuminol (**4**) was purified by dry-flash chromatography on silica gel using *n*-hexane/Et_2_O mixtures of increasing polarity as the eluents. The yield of 3-aminocuminol (**4**; 9.44 g (57.21 mmol)) was 97%. The spectral data of **4** are given below:

*(3-Amino-4-isopropylphenyl)methanol (**4**; 3-aminocuminol)*: retention index (RI) = 1587 (DB-5MS column); UV (CH_3_CN) λ_max_(log ε) 292 (3.46), 241 (3.91), 207 (4.51) nm; FTIR (neat; cm^−1^) 3378, 3185, 2959, 2928, 2867, 2838, 1621, 1577, 1508, 1447, 1425, 1381, 1367, 1311, 1284, 1253, 1227, 1160, 1060, 1045, 988, 955, 921, 889, 853, 798, 730; MS (EI), *m*/*z* (%) 166(4), 165(36) [M^+^], 151(10), 150(100), 134(3), 133(3), 132(8), 130(3), 122(3), 121(3), 120(14), 118(5), 117(5), 115(5), 106(9), 105(8), 104(3), 103(6), 94(9), 93(6), 91(8), 79(5), 78(3), 77(13), 65(5), 59(4), 51(3), 41(3), 39(4); analyzed C 72.71, H 9.12, N 8.49, O 9.68%, calculated for C_10_H_15_NO, C 72.69, H 9.15, N 8.48, O 9.68%; ^1^H NMR (CDCl_3_) δ 1.24 (*d*, *J* = 6.8 Hz, 6H, CH_3_-9 and CH_3_-10), 2.87 (*sept*, *J* = 6.8 Hz, 1H, CH-8), 3.14 (*br s*, 3H, OH and NH_2_), 4.54 (*br s*, 2H, CH_2_-7), 6.66 (*d*, *J* = 1.6 Hz, 1H, CH-2), 6.75 (*dd*, *J* = 7.8, 1.6 Hz, 1H, CH-6), 7.11 (*d*, *J* = 7.8 Hz, 1H, CH-5); ^13^C NMR (CDCl_3_) δ 22.3 (C-9, and C-10), 27.5 (C-8), 65.1 (C-7), 114.5 (C-2), 117.7 (C-6), 125.6 (C-5), 132.2 (C-4), 139.4 (C-1), 143.4 (C-3).

#### 3.4.4. Synthesis of 3-Hydroxycuminol

Nine grams (54.55 mmol) of 3-aminocuminol (**4**) were dissolved in a solution of concentrated sulfuric acid (12 mL) in 30 mL of water at 0 °C with efficient stirring. After 15 min, an aqueous solution of sodium nitrite (3.76 g (54.55 mmol) of NaNO_2_ dissolved in 10 mL of water) was dropwise added to this mixture (temperature was controlled in an interval of 0 to 5 °C). The solution was stirred for 2 h at room temperature and extracted three times with Et_2_O. The organic layers were combined, dried over anhydrous MgSO_4_, and concentrated under reduced pressure. Crude 3-hydroxycuminalcohol (**5**) was purified by dry-flash chromatography on silica gel using an *n*-hexane/Et_2_O mixture. The yield of 3-hydroxycuminol (**5**; 6.07 g (36.57 mmol)) was 67%. The spectral data of **5** are given below:

*5-(Hydroxymethyl)-2-isopropylphenol (**5**; 3-hydroxycuminol)*: retention index (RI) = 1563 (DB-5MS column); UV (CH_3_CN) λ_max_(log ε) 282 (3.50), 276 (3.49), 218 (3.94), 202 (4.22) nm; FTIR (neat; cm^−1^) 3271, 2960, 2870, 1616, 1585, 1504, 1424, 1382, 1362, 1288, 1236, 1193, 1152, 1112, 1087, 1060, 1002, 939, 863, 817, 755, 739, 710; MS (EI), *m*/*z* (%) 167(4), 166(37) [M^+^], 152(10), 151(100), 135(4), 133(10), 123(4), 121(22), 115(6), 107(7), 105(10), 103(10), 95(11), 93(4), 91(12), 79(8), 78(3), 77(17), 65(5), 53(3), 51(4), 41(3), 39(4); analyzed C 72.31, H 8.45, O 19.24%, calculated for C_10_H_14_O_2_, C 72.26, H 8.49, O 19.25%; ^1^H NMR (CDCl_3_) δ 1.21 (*d*, *J* = 6.9 Hz, 6H, CH_3_-9 and CH_3_-10), 3.22 (*sept*, *J* = 6.9 Hz, 1H, CH-8), 4.27 (*br s*, 2H, C_10_-OH and C_3_-OH), 4.54 (*br s*, 2H, CH_2_-7), 6.78–6.81 (overlapping peaks, 2H, CH-2, CH-6), 7.14 (*d*, *J* = 8.2 Hz, 1H, CH-5); ^13^C NMR (CDCl_3_) δ 22.6 (C-9, and C-10), 26.8 (C-8), 65.0 (C-7), 114.2 (C-6), 119.3 (C-2), 126.5 (C-5), 134.5 (C-1), 138.9 (C-4), 153.3 (C-3).

#### 3.4.5. Synthesis of 3-Methoxycuminol

3-Hydroxycuminol (**5**; 5 g, 30.12 mmol) was added to a suspension of anhydrous potassium carbonate (16.58 g, 120 mmol) in acetone (50 mL). Then, methyl iodide (8.5 g, 60 mmol) was added, and the solution was heated for 4 h under reflux. After that, another portion of methyl iodide (4.3 g, 30.30 mmol) was added, and the solution was stirred at room temperature for another 24 h and concentrated in vacuo. The residue was dissolved in water (50 mL) and extracted three times with Et_2_O. The combined organic extracts were dried with anhydrous MgSO_4_ and concentrated under reduced pressure. Crude 3-methoxycuminol (**6**) was purified by dry-flash chromatography on silica gel using *n*-hexane/Et_2_O mixtures of increasing polarity as the eluents. The yield of 3-methoxycuminol (**6**; 5.2 g (28.89 mmol)) was 96%. The spectral data of **6** are given below:

*(4-Isopropyl-3-methoxyphenyl)methanol (**6**; 3-methoxycuminol)*: retention index (RI) = 1492 (DB-5MS column); UV (CH_3_CN) λ_max_(log ε) 318 (3.12), 281 (3.63), 275 (3.62), 223 (4.13), 203 (4.46) nm; FTIR (neat; cm^−1^) 3310, 2958, 2869, 1612, 1579, 1505, 1462, 1416, 1382, 1360, 1287, 1254, 1191, 1160, 1093, 1061, 1040, 921, 854, 818, 733; MS (EI), *m*/*z* (%) 181(3), 180(28) [M^+^], 166(11), 165(100), 149(4), 147(3), 135(9), 121(5), 117(7), 115(7), 109(4), 107(4), 105(21), 103(7), 91(17), 79(11), 78(4), 77(15), 65(5), 53(3), 51(4), 41(4), 39(4); analyzed C 73.28, H 8.96, O 17.76%, calculated for C_11_H_16_O_2_, C 73.30, H 8.95, O 17.75%; ^1^H NMR (CDCl_3_) δ 1.20 (*d*, *J* = 6.9 Hz, 6H, CH_3_-9 and CH_3_-10), 2.00 (*br s*, 1H, OH), 3.30 * (*septddd*, *J* = 6.9, 0.4, 0.3, 0.25 Hz, 1H, CH-8), 3.83 (*s*, 3H, CH_3_-11), 4.63 * (*dd*, *J* = 0.6, 0.5 Hz, 2H, CH_2_-7), 6.8745 * (*dtd*, *J* = 1.6, 0.5, 0.25 Hz, 1H, CH-2), 6.8877 * (*ddtd*, *J* = 7.34, 1.6, 0.6, 0.3 Hz, 1H, CH-6), 7.1787 * (*dd*, *J* = 7.34, 0.4 Hz, 1H, CH-5); ^13^C NMR (CDCl_3_) δ 22.7 (C-9, and C-10), 26.5 (C-8), 55.4 (C-11), 65.4 (C-7), 109.1 (C-2), 119.0 (C-6), 126.1 (C-5), 136.5 (C-1), 139.4 (C-4), 156.9 (C-3). * The values of chemical shift and coupling constants were determined by a simulation of the ^1^H NMR spectrum (manual iterative full spin analysis (Radulović et al., 2019).

### 3.5. Synthesis of 3-Methoxycuminyl Esters

Esters of 3-methoxycuminol (**6**) with isobutanoic (**7**), butanoic (**8**), 2-methylbutanoic (**9**), 3-methylbutanoic (**10**), and pentanoic (**11**) acids were prepared according to the general Steglich approach (*N,N*’-dicyclohexylcarbodiimide (DCC)/4-(dimethylamino)pyridine (DMAP)). A solution of 3-methoxycuminol (400 mg, 2.2 mmol), the appropriate carboxylic acid (2.3 mmol), DMAP (80 mg, 0.7 mmol), and DCC (470 mg, 2.3 mmol) in 30 mL of dry CH_2_Cl_2_ was stirred overnight at room temperature. Then, the precipitated urea was filtered off and the filtrate was concentrated in vacuo. The resulting residue was purified by dry-flash chromatography on silica gel using an *n*-hexane/Et_2_O mixture (19:1, *v*/*v*) as the eluent. The spectral data (except NMR spectral data for **7**, **9**, and **10** that are given in Table 2) of the synthesized esters **7**–**11** are given below and in the Supplementary Materials:

*4-Isopropyl-3-methoxybenzyl isobutanoate (**7**; 3-methoxycuminyl isobutanoate)*: colorless liquid; retention index (RI) = 1725 (DB-5MS column); UV (CH_3_CN) λ_max_(log ε) 281 (3.71), 275 (3.72), 224 (4.25), 204 (4.57) nm; FTIR (neat; cm^−1^) 2961, 2872, 1732, 1613, 1581, 1507, 1463, 1418, 1386, 1362, 1342, 1289, 1256, 1188, 1148, 1110, 1094, 1062, 1041, 965, 926, 852, 817, 758, 735; MS (EI), *m*/*z* (%) 251(8), 250(54) [M^+^], 236(14), 235(100), 181(8), 180(76), 179(6), 165(5), 164(5), 163(37), 162(3), 149(3), 148(13), 147(16), 137(25), 135(9), 133(7), 132(3), 131(7), 121(15), 119(5), 118(3), 117(16), 116(5), 115(16), 109(12), 105(9), 103(6), 91(15), 79(4), 78(3), 77(8), 71(11), 65(3), 55(3), 43(21), 41(8), 39(3); analyzed C 71.95, H 8.85, O 19.20%, calculated for C_15_H_22_O_3_, C 71.97, H 8.86, O 19.17%.

*4-Isopropyl-3-methoxybenzyl butanoate (**8**; 3-methoxycuminyl butanoate)*: colorless liquid; retention index (RI) = 1776 (DB-5MS column); UV (CH_3_CN) λ_max_(log ε) 281 (3.29), 275 (3.30), 224 (3.83), 201 (4.52) nm; FTIR (neat; cm^−1^) 2961, 2873, 1733, 1613, 1581, 1507, 1461, 1418, 1382, 1349, 1288, 1256, 1165, 1094, 1062, 1040, 972, 921, 851, 817, 734; MS (EI), *m*/*z* (%) 251(7), 250(49) [M^+^], 236(14), 235(100), 181(8), 180(77), 179(3), 165(6), 164(3), 163(19), 162(3), 149(3), 148(9), 147(15), 137(23), 135(5), 133(6), 132(3), 131(5), 121(10), 119(5), 118(3), 117(13), 116(4), 115(13), 109(8), 105(7), 103(5), 91(12), 79(3), 78(3), 77(7), 71(12), 65(3), 43(13), 41(6), 39(3); analyzed C 71.96, H 8.84, O 19.20%, calculated for C_15_H_22_O_3_, C 71.97, H 8.86, O 19.17%; ^1^H NMR (CDCl_3_) δ 0.95 (*t*, *J* = 7.4 Hz, 3H, CH_3_-15), 1.20 (*d*, *J* = 6.9 Hz, 6H, CH_3_-9 and CH_3_-10), 1.68 (*sext*, *J* = 7.4 Hz, 1H, CH_2_-14), 2.34 (*t*, *J* = 7.4 Hz, 3H, CH_2_-13), 3.30 (*sept*, *J* = 6.9 Hz, 1H, CH-8), 3.83 (*s*, 3H, CH_3_-11), 5.08 (*s*, 2H, CH_2_-7), 6.83 (*d*, *J* = 1.5 Hz, 1H, CH-2), 6.92 (*dd*, *J* = 7.7, 1.5 Hz, 1H, CH-6), 7.19 (*d*, *J* = 7.7 Hz, 1H, CH-5); ^13^C NMR (CDCl_3_) δ 13.7 (C-15), 18.5 (C-14), 22.6 (C-9, and C-10), 26.6 (C-8), 36.3 (C-13), 55.4 (C-11), 66.2 (C-7), 110.3 (C-2), 120.5 (C-6), 126.1 (C-5), 134.5 (C-1), 137.1 (C-4), 156.9 (C-3), 173.6 (C-12).

*4-Isopropyl-3-methoxybenzyl 2-methylbutanoate (**9**; 3-methoxycuminyl 2-methylbutanoate)*: colorless liquid; retention index (RI) = 1808 (DB-5MS column); UV (CH_3_CN) λ_max_(log ε) 281 (3.40), 275 (3.42), 224 (3.95), 200 (4.72) nm; FTIR (neat; cm^−1^) 2962, 2936, 2874, 1731, 1613, 1581, 1507, 1461, 1418, 1382, 1350, 1289, 1257, 1178, 1144, 1118, 1094, 1062, 1041, 1013, 957, 850, 817, 757, 735; MS (EI), *m*/*z* (%) 265(8), 264(47) [M^+^], 249(77), 181(11), 180(100), 179(6), 178(4), 165(5), 164(6), 163(42), 162(3), 149(3), 148(11), 147(13), 137(24), 135(7), 133(6), 131(6), 121(15), 119(4), 118(3), 117(14), 116(5), 115(14), 109(11), 105(8), 103(5), 91(13), 85(6), 79(4), 78(3), 77(7), 57(23), 55(4), 41(8), 39(3); analyzed C 71.93, H 8.88, O 19.19%, calculated for C_15_H_22_O_3_, C 71.97, H 8.86, O 19.17%.

*4-Isopropyl-3-methoxybenzyl 3-methylbutanoate (**10**; 3-methoxycuminyl 3-methylbutanoate)*: colorless liquid; retention index (RI) = 1817 (DB-5MS column); UV (CH_3_CN) λ_max_(log ε) 281 (3.00), 275 (3.02), 224 (3.54), 200 (4.32) nm; FTIR (neat; cm^−1^) 2958, 2871, 1732, 1613, 1581, 1507, 1463, 1418, 1371, 1350, 1291, 1255, 1182, 1164, 1117, 1093, 1062, 1041, 986, 926, 851, 816, 736; MS (EI), *m*/*z* (%) 265(7), 264(42) [M^+^], 250(12), 249(79), 181(11), 180(100), 179(3), 165(6), 164(5), 163(29), 162(3), 149(3), 148(9), 147(13), 137(26), 135(5), 133(6), 131(5), 121(11), 119(4), 118(3), 117(12), 116(4), 115(12), 109(8), 105(7), 103(5), 91(11), 85(9), 79(3), 78(3), 77(6), 57(12), 55(3), 43(5), 41(7), 39(3); analyzed C 71.97, H 8.82, O 19.21%, calculated for C_15_H_22_O_3_, C 71.97, H 8.86, O 19.17%.

*4-Isopropyl-3-methoxybenzyl pentanoate (**11**; 3-methoxycuminyl pentanoate)*: colorless liquid; retention index (RI) = 1863 (DB-5MS column); UV (CH_3_CN) λ_max_(log ε) 281 (3.25), 275 (3.26), 224 (3.79), 200 (4.56) nm; FTIR (neat; cm^−1^) 2958, 2872, 1736, 1613, 1581, 1507, 1463, 1418, 1380, 1349, 1288, 1259, 1166, 1094, 1062, 1020, 851, 804; MS (EI), *m*/*z* (%) 265(7), 264(40) [M^+^], 250(13), 249(80), 181(12), 180(100), 179(3), 165(6), 164(5), 163(26), 162(4), 149(4), 148(11), 147(14), 137(28), 135(6), 133(7), 131(6), 121(13), 119(5), 118(3), 117(16), 116(5), 115(16), 109(9), 105(9), 103(6), 91(15), 85(13), 79(4), 78(3), 77(8), 57(16), 55(6), 43(3), 41(9), 39(3); analyzed C 71.95, H 8.86, O 19.19%, calculated for C_15_H_22_O_3_, C 71.97, H 8.86, O 19.17%; ^1^H NMR (CDCl_3_) δ 0.91 (*t*, *J* = 7.4 Hz, 3H, CH_3_-16), 1.20 (*d*, *J* = 6.9 Hz, 6H, CH_3_-9 and CH_3_-10), 1.35 (*sext*, *J* = 7.4 Hz, 1H, CH_2_-15), 1.63 (*qui*, *J* = 7.4 Hz, 1H, CH_2_-14), 2.36 (t, *J* = 7.4 Hz, 3H, CH_2_-13), 3.30 (*sept*, *J* = 6.9 Hz, 1H, CH-8), 3.83 (*s*, 3H, CH_3_-11), 5.08 (*s*, 2H, CH_2_-7), 6.83 (*d*, *J* = 1.5 Hz, 1H, CH-2), 6.92 (*dd*, *J* = 7.7, 1.5 Hz, 1H, CH-6), 7.19 (*d*, *J* = 7.7 Hz, 1H, CH-5); ^13^C NMR (CDCl_3_) δ 13.7 (C-16), 22.3 (C-15), 22.6 (C-9, and C-10), 26.6 (C-8), 27.1 (C-14), 34.1 (C-13), 55.4 (C-11), 66.3 (C-7), 110.3 (C-2), 120.5 (C-6), 126.1 (C-5), 134.5 (C-1), 137.1 (C-4), 156.9 (C-3), 173.8 (C-12).

### 3.6. Biological Activity

#### 3.6.1. Animals and Housing

Disease-free male Wistar rats (300–350 g) were obtained from the Vivarium of the Scientific Research Center for Biomedicine, Faculty of Medicine, University of Niš, Serbia. The animals were maintained under standard husbandry conditions with a temperature of 23 ± 2 °C, relative humidity of 55 ± 10%, and 12/12 h light/dark cycle. All animals were fed with commercially available standard laboratory food pellets, and water was provided ad libitum. The experiments were performed following the declaration of Helsinki and European Community guidelines for the ethical handling of laboratory animals (EU Directive of 2010; 2010/63/EU), and the experimental protocols were commenced after being approved by the institutional animal ethics committee (No. 323-07-06862/2016-05/2).

#### 3.6.2. Preparation of Distal Colon Strips

After the animals were sacrificed, their abdomens were opened and the distal colon, a few centimeters from the anus, was dissected and placed in a Petri dish filled with Tyrode’s solution of the following composition: 136.75 mM NaCl, 2.68 mM KCl, 1.05 mM MgCl_2_, 1.80 mM CaCl_2_, 0.42 mM NaH_2_PO_4_, 11.90 mM NaHCO_3_, and 5.55 mM glucose, pH 7.4. The luminal contents were flushed out using the same solution, and the distal colon strips (approximately 1.0–1.5 cm in length) were longitudinally mounted in a 20 mL tissue bath containing Tyrode’s solution bubbled with a mixture containing 5% CO_2_ (*v*/*v*) in oxygen and maintained at 37 °C. One edge of the distal colon was anchored with a silk suture to the bottom of the organ bath, and the other edge was connected using a cotton thread to the isometric force transducer (Elunit, Belgrade, Serbia). The data were recorded and analyzed with PC Biodata-F software (Elunit, Belgrade, Serbia).

#### 3.6.3. Exposition of the Distal Colon to *P. dysenterica* Essential-Oil Sample

After a stabilization period of 45 min, the distal colon tissue was exposed to increasing concentrations of the essential-oil sample (**EO**) from 0.025 µg/mL to 0.25 mg/mL. The two samples of essential oil were of very similar composition, so they were pooled and used in the biological assays. Due to the poor solubility of the essential oil in Tyrode’s solution, higher concentrations (0.25 mg/mL) were not tested. The distal colon strip was exposed to each **EO** concentration for 5 min, after which the tissue segments were washed with fresh Tyrode’s solution and left to stabilize for 10 min before being exposed to the corresponding **EO** concentration. Different **EO** concentrations were tested in parallel using two segments of the distal colon, and the experiments were repeated four times on distal colon segments obtained from different animals.

#### 3.6.4. Measurement of Changes in the Contraction Pattern

For each tested concentration of the essential-oil sample (**EO**), the maximal and minimal amplitudes were measured during 5 min of exposure to the **EO** sample. The change in the amplitude of distal colon contractions, relative to the one measured in the period before the addition of the test compounds, was expressed as a percentage and used to calculate EC values. The number of contractions was counted before the addition of the **EO** samples or papaverine (positive control). For each of the tested concentrations of the **EO**, the number of contractions was counted during each minute of a 5 min exposure period, and the obtained data were used to calculate the percentage of the increase or decrease in the number of distal colon contractions.

#### 3.6.5. AChE (Acetylcholinesterase) Inhibitory Activity

The AChE inhibitory activities of the **EO** sample, commercially available cuminal (**1**), and synthesized compounds **2–11** were measured by a quantitative colorimetric assay based on Ellman’s method [41]. Briefly, mixtures of 25 µL of AChE (0.22 U/mL in buffer A), 50 µL of buffer A (50 mM Tris–HCl, pH 7.9, containing 0.1% bovine serum albumin), and 25 µL of the test solutions (3.9–1250 µg of **EO** per mL or 0.0095–5 mmol/L of compounds **1–11** in absolute methanol; ten different concentrations) were incubated for 20 min at 37 °C. After that, Ellman’s reagent (125 µL of 3 mM 5,5′-dithiobis(2-nitrobenzoic acid) in buffer B (50 mM Tris–HCl, pH 7.9, containing 0.1 M NaCl and 0.02 M MgCl_2_ × 6H_2_O)) and 25 µL of 15 mM acetylthiocholine iodide were added, and the absorbance at 405 nm was recorded every 15 s over 15 min. Absolute methanol was used as the negative control (10%, *v*/*v*, in the plate well). For validation, different concentrations of rivastigmine served as a positive control. Each experiment was carried out in triplicate and repeated three times.

#### 3.6.6. Test Microorganisms

The essential oil of *P. dysenterica* and the synthesized compounds were tested against a panel of microbial strains belonging to the American Type Culture Collection reference strains; Gram-positive bacteria (*Staphylococcus aureus* (ATCC 6538), *S. epidermidis* (ATCC 12228), *Bacillus cereus* (ATCC 11778), and *Kocuria rhizophila* (formerly *Sarcina lutea* under the same ATCC number of ATCC 9341)), Gram-negative bacteria (*Pseudomonas aeruginosa* (ATCC 9027), *Escherichia coli* (ATCC 8739), *Salmonella enterica* subsp. *enterica* serovar Enteritidis (ATCC 13076) and *Acinetobacter baumanii* (ATCC 19606)), yeast *Candida albicans* (ATCC 10231) and mold *Aspergillus brasiliensis* (ATCC 16404). The testing was also performed against eight isolates of *Salmonella* spp. obtained from human stool samples. Bacterial strains were maintained on Nutrient Agar (NA) at 37 °C and fungal strains were maintained on Sabouraud Dextrose Agar (SDA) at 30 °C at the Microbiology Laboratory (Department of Biology and Ecology, Faculty of Sciences and Mathematics, University of Niš).

#### 3.6.7. Screening of Antimicrobial Activity (Microdilution Method)

Antimicrobial activity was evaluated using a broth microdilution method in microtiter plates, as described earlier [42]. Briefly, cell suspensions standardized to McFarland standard No. 0.5 (DEN-1, Biosan) were made using the test microorganisms’ overnight cultures (18 h). Stock solutions of the synthesized compounds were made in pure DMSO and further diluted with an appropriate sterile broth (Sabouraud Dextrose or Mueller Hinton broth); the lowest dilution of the solvent (10%, *v*/*v*) did not affect bacterial or fungal growth. These solutions were further serially diluted (the diluting factor 2) in a concentration range of 0.01–4.00 g/L. After making the dilutions of the test substances, the inoculum was added to all wells and the plates were incubated at 37 °C for 24 h in the case of bacteria or at 30 °C for 48 h in the case of fungi. Streptomycin, chloramphenicol, and nystatin served as positive controls, and one non-inoculated well, free of any antimicrobial agent, was also included to ensure medium sterility. The bacterial growth was determined by adding 20 μL of a 0.5% triphenyltetrazolium chloride (TTC) aqueous solution. MIC was defined as the lowest concentration of the test compound that inhibited visible growth (red-colored pellet on the bottom of the wells after the addition of TTC). All experiments were performed in triplicate.

#### 3.6.8. Evaluation of Acute Toxicity in the Model of *Artemia salina*

The method for *Artemia salina* (brine shrimp) cyst hatching used here was previously described by Radulović et al. [42]. The final concentrations of the tested samples (**EO** and synthesized compounds **5**, **6**, **7**, **9**, and **10**) were as follows: 3.9, 7.8, 15.6, 31.3, 62.5, and 125 µg/mL. The final concentration of DMSO was much less than 1% (*v*/*v*). The tested samples were not aerated, and the test dishes were left at room temperature under constant illumination; brine shrimps were not fed during the test. Dead nauplii were counted after 24 and 48 h. Statistical analysis determined a concentration lethal to 50% of nauplii (LC_50_). Sodium dodecyl sulphate (SDS) was used as a positive control. DMSO was inactive under the stated conditions, as demonstrated by a negative control. All the tests were performed in triplicate and repeated twice.

#### 3.6.9. Preparation and Culture of Rat Macrophages

Animals were sacrificed and opened under sterile conditions. To obtain a single-cell suspension, the peritoneal cavity was washed with PBS. Suspensions of the rat peritoneal macrophages obtained after centrifugation at 1200 rpm for 10 min (at 4 °C) were re-suspended in an RPMI medium, cell density was adjusted to 2.5 × 10^6^ cells/mL, and their viability was confirmed using trypan blue staining (>95% of viable cells). These cells were further cultured in 96-well cell culture plates (Greiner Bio-One, Frickenhausen, Germany); each well contained 100 µL of the suspension containing the RPMI medium. Control cells were cultured with 100 µL of RPMI per well. Dexamethasone (a steroid drug with anti-inflammatory and immunosuppressant effects) was used as the positive control at a final concentration of 1 × 10^−4^ M in the wells. The **EO** sample was assayed in five different concentrations from 100 to 0.001 µg/mL. The compounds were tested in doses from 10^−4^ to 10^−8^ mol/L. The plates were incubated at 37 °C for 24 h under an atmosphere of 95% air and 5% CO_2_ (*v*/*v*). All experiments were performed in quadruplicate and repeated three times.

#### 3.6.10. Determination of Cell Viability by MTT Assay

The mitochondrial-dependent reduction of MTT to formazan crystals was used to determine cell viability in cultures. The assay was performed 24 h after the incubation of macrophages with different concentrations of the oil or appropriate control. After the removal of the cell medium, 100 µL of a fresh RPMI medium and an MTT solution (5 mg/mL) were added, and the plates were incubated for an additional 4 h. Acidified isopropanol was added to all wells, and the plates were shaken to dissolve the dark blue crystals of the formazan. A few minutes after the dissolution of crystals, the absorbance was read at 550 nm [16] using an automated microplate reader (Multiscan Ascent, Labsystems, Helsinki, Finland).

#### 3.6.11. Statistical Treatment of the Results of In Vitro Animal Assays

The results are expressed as the mean ± SD. Statistically significant differences between the treatments in in vitro assays conducted on isolated rat distal colon tissue and peritoneal macrophages were determined by a One-Way Analysis of Variance (ANOVA) followed by Tukey’s post hoc test for multiple comparisons (GraphPad Prism version 5.03, San Diego, CA, USA). Probability values (*p*) ≤ 0.05 were considered to be statistically significant.

## 4. Conclusions

A sum of the organic synthesis and GC–MS, UV–Vis, FTIR, and 1D and 2D NMR analyses provide unequivocal proof that *Pulicaria dysenterica* produces 3-methoxycuminyl esters: isobutanoate (major essential oil constituent), 2-methylbutanoate (a new natural product), and 3-methylbutanoate (a rare natural product that was only identified as a constituent of *Inula viscosa* essential oil [11]). The herein presented results regarding the acute toxicity, antimicrobial activity, AChE inhibitory activity, antispasmodic activity, and cytotoxic properties of the essential oil and 3-methoxycuminyl esters further corroborate the fact that the *P. dysenterica* essential oil could be responsible for the ethnopharmacological use of this taxon for the treatment of some digestive problems. Surprisingly, although the essential oil moderately inhibited acetylcholinesterase (at the concentration of 0.125 μg/mL, it caused a 14.9% reduction in acetylcholinesterase activity), it did not affect spontaneous distal colon contractions. Additionally, the oil and its constituents only exerted a high cytotoxic potential when cells were exposed to the highest tested concentrations; in the subsequently tested dilutions, the toxicity almost wholly disappeared.

Based on the present results, the essential oil of *P. dysenterica* can be considered a natural agent that can be further explored as a crop for treating digestive problems caused by some microorganisms. However, although we have provided new data regarding the phytochemistry and bioactivity of *P. dysenterica*’*s* essential oil and oil constituents, this is just a tiny piece of the whole picture. We focused our attention on the essential oil and several volatile metabolites. To confirm *P. dysenterica* as medicinal taxa and potential industrial crops, we need to provide answers about the non-volatile metabolites and their bioactivity/toxicity.

## Figures and Tables

**Table 1:** Chemical composition of the essential oil of *Pulicaria dysenterica* (L.) Bernh. from Serbia.

RI ^a^		Constituents ^b^	C ^c^	Samples ^d^				ID ^e^
Exp	Lit			A		B		
				%	c	%	c	
765	765	(*Z*)-2-Penten-1-ol	FAD	tr	0.07	-	-	f, g
801	801	Hexanal	FAD	tr	0.11	-	-	f, g, h
830	828	Furfural	FAD	tr	0.11	-	-	f, g, h
845	841	(*Z*)-2-Hexenal	FAD	tr	0.11	-	-	f, g
852	846	(*E*)-2-Hexenal	FAD	0.3	1.14	-	-	f, g
865	863	1-Hexanol	FAD	0.1	0.35	tr	0.10	f, g, h
901	901	Heptanal	FAD	tr	0.11	-	-	f, g, h
910	907	(2*E*,4*E*)-2,4-Hexadienal	FAD	tr	0.11	tr	0.11	f, g
935	932	α-Pinene	MH	tr	0.08	-	-	f, g, h
949	955	4-Methyl-1-hexanol	FAD	-	-	tr	0.10	f, g
950	947	(*E*)-2-Heptenal	FAD	tr	0.11	tr	0.11	f, g
954	952	Benzaldehyde	SM	tr	0.11	tr	0.11	f, g, h
973	969	Sabinene	MH	tr	0.08	-	-	f, g
976	974	β-Pinene	MH	-	-	tr	0.08	f, g, h
977	974	1-Octen-3-ol	FAD	tr	0.11	tr	0.10	f, g, h
984	980	2,3-Octanedione	FAD	tr	0.11	tr	0.10	f, g
985	981	6-Methyl-5-hepten-2-one	FAD	tr	0.11	tr	0.10	f, g
992	988	Myrcene	MH	tr	0.08	-	-	f, g
993	984	2-Pentylfuran	FAD	-	-	tr	0.12	f, g
993	993	Butyl butanoate	FAD	tr	0.13	-	-	f, g
994	997	(2*E*,4*Z*)-2,4-Heptadienal	FAD	tr	0.11	-	-	f, g
996	998	3-Methoxypyridine	FAD	-	-	tr	0.12	f, g
999	999	Yomogi alcohol	MO	-	-	tr	0.10	f, g, h
1000	1000	Decane	FAD	-	-	tr	0.07	f, g, h
1002	1001	(*E*)-2-(2-Pentenyl)furan	FAD	1.0	4.30	tr	0.12	f, g
1002	998	Octanal	FAD	0.1	0.38	tr	0.11	f, g
1012	1005	(2*E*,4*E*)-2,4-Heptadienal	FAD	0.1	0.38	tr	0.11	f, g
1014	1013	α-Terpinene	MH	tr	0.08	-	-	f, g
1028	1021	*p*-Cymene	MH	tr	0.08	-	-	f, g
1033	1024	Limonene	MH	tr	0.08	tr	0.08	f, g, h
1034	1026	1,8-Cineole	MO	tr	0.13	-	-	f, g, h
1036	1026	Benzyl alcohol	SM	-	-	tr	0.10	f, g, h
1036	1025	(*Z*)-β-Ocimene	MH	tr	0.08	-	-	f, g
1049	1036	Phenylacetaldehyde	SM	tr	0.11	tr	0.11	f, g
1051	1044	(*E*)-β-Ocimene	MH	tr	0.08	-	-	f, g
1058	1049	(*E*)-2-Octenal	FAD	tr	0.11	-	-	f, g
1063	1054	γ-Terpinene	MH	-	-	tr	0.08	f, g
1064	1056	Artemisia ketone	MO	-	-	tr	0.10	f, g
1069	1060	(*E*)-2-Octen-1-ol	FAD	-	-	tr	0.10	f, g
1072	1063	1-Octanol	FAD	tr	0.11	tr	0.10	f, g, h
1073	1064	*m*-Tolualdehyde	SM	tr	0.11	tr	0.11	f, g
1073	1071	(3*E*,5*E*)-3,5-Octadien-2-one	FAD	-	-	tr	0.10	f, g
1075	1067	*cis*-Linalool oxide (furanoid)	MO	tr	0.13	-	-	f, g
1080	1080	Artemisia alcohol	MO	-	-	tr	0.10	f, g, h
1082	1077	4-Methylbenzaldehyde	SM	-	-	tr	0.11	f, g
1093	1086	Terpinolene	MH	tr	0.08	tr	0.08	f, g
1100	1100	Undecane	FAD	-	-	tr	0.00	f, g, h
1100	1095	Linalool	MO	0.1	0.35	0.1	0.32	f, g, h
1105	1100	Nonanal	FAD	0.1	0.38	0.2	0.70	f, g, h
1107	1107	(3*E*)-6-Methyl-3,5-heptadien-2-one	FAD	-	-	tr	0.10	f, g
1116	1118	*cis*-*p*-Menth-2-en-1-ol	MO	tr	0.11	tr	0.10	f, g
1117	1119	*trans*-*p*-Mentha-2,8-dien-1-ol	MO	-	-	tr	0.10	f, g
1128	1134	*cis*-*p*-Mentha-2,8-dien-1-ol	MO	-	-	tr	0.10	f, g
1133	1136	*trans*-*p*-Menth-2-en-1-ol	MO	-	-	tr	0.10	f, g
1134	1137	(1*R* *,3*S* *,5*R* *)-Sabinol (syn. *trans*-sabinol)	MO	-	-	tr	0.10	f, g, h
1139	1140	*trans*-Verbenol	MO	-	-	tr	0.10	f, g
1141	1141	Camphor	MO	-	-	0.1	0.33	f, g, h
1143	1142	(*Z*)-3-Hexenyl isobutanoate	FAD	tr	0.13	-	-	f, g
1144	1150	(2*E*,6*Z*)-2,6-Nonadienal	FAD	-	-	tr	0.11	f, g
1156	1154	Nerol oxide	MO	0.1	0.43	0.1	0.40	f, g
1160	1157	(*E*)-2-Nonenal	FAD	tr	0.11	tr	0.11	f, g
1161	1154	Albene	O	-	-	tr	0.08	f, g
1162	1165	3,4-Dimethylphenol	SM	-	-	tr	0.10	f, g
1162	1160	Pinocarvone	MO	-	-	0.1	0.33	f, g
1168	1165	Lavandulol	MO	0.2	0.70	-	-	f, g, h
1170	1165	Borneol	MO	tr	0.11	tr	0.10	f, g, h
1170	1166	*p*-Mentha-1,5-dien-8-ol	MO	-	-	tr	0.10	f, g
1177	1172	*cis*-Pinocamphone	MO	-	-	tr	0.10	f, g
1180	1174	Terpinen-4-ol	MO	-	-	tr	0.10	f, g
1186	1179	*p*-Cymen-8-ol	MO	tr	0.11	tr	0.10	f, g
1192	1186	Butyl hexanoate	FAD	tr	0.13	-	-	f, g
1194	1191	Hexyl butanoate	FAD	0.1	0.42	-	-	f, g
1195	1186	α-Terpineol	MO	0.1	0.35	0.2	0.65	f, g
1199	1190	Methyl salicylate	SM	tr	0.13	-	-	f, g
1199	1196	Safranal	C	tr	0.11	tr	0.11	f, g
1200	1200	Dodecane	FAD	-	-	tr	0.07	f, g, h
1201	1190	Myrtenal	MO	-	-	tr	0.11	f, g
1206	1201	Decanal	FAD	tr	0.11	tr	0.11	f, g, h
1210	1207	*trans*-Piperitol	MO	-	-	tr	0.10	f, g
1219	1221	8,9-Dehydrothymol	MO	0.1	0.35	0.1	0.32	f, g
1226	1217	β-Cyclocitral	MO	tr	0.11	tr	0.11	f, g
1230	1227	Nerol	MO	2.0	7.04	1.9	6.17	f, g, h
1237	1232	Methyl thymyl ether	MO	0.1	0.43	tr	0.12	f, g
1240	1235	*trans*-Chrysanthenyl acetate	MO	-	-	tr	0.12	f, g
1244	1235	Neral	MO	tr	0.11	tr	0.11	f, g, h
1246	1240	Carvacryl methyl ether	MO	-	-	tr	0.12	f, g
1251	1249	Geraniol	MO	tr	0.11	-	-	f, g, h
1257	1250	*trans*-Piperitone epoxide	MO	-	-	tr	0.12	f, g
1262	1260	(*E*)-2-Decenal	FAD	tr	0.11	tr	0.11	f, g
1265	1267	Nonanoic acid	FAD	tr	0.13	tr	0.12	f, g, h
1272	1264	Geranial	MO	tr	0.11	-	-	f, g, h
1272	1266	1-Decanol	FAD	-	-	0.1	0.32	f, g, h
1290	1287	Bornyl acetate	MO	-	-	tr	0.12	f, g
1290	1288	Lavandulyl acetate	MO	0.1	0.42	-	-	f, g
1292	1289	Thymol	MO	tr	0.11	tr	0.10	f, g, h
1293	1298	*trans*-Pinocarvyl acetate	MO	-	-	tr	0.12	f, g
1294	1992	Dihydroedulan IA	C	-	-	tr	0.07	f, g
1295	1292	(2*E*,4*Z*)-2,4-Decadienal	FAD	tr	0.11	tr	0.11	f, g
1296	1290	Indole	SM	-	-	tr	0.10	f, g
1300	1300	Tridecane	FAD	-	-	0.1	0.25	f, g, h
1301	1298	Theaspirane A	C	-	-	tr	0.08	f, g
1308	1305	Undecanal	FAD	tr	0.11	tr	0.11	f, g, h
1317	1315	Theaspirane B	C	-	-	-	-	f, g
1318	1315	(2*E*,4*E*)-2,4-Decadienal	FAD	0.1	0.38	tr	0.11	f, g
1322	1319	(*Z*)-3-Hexenyl tiglate	FAD	tr	0.13	-	-	f, g
1327	1324	Myrtenyl acetate	MO	tr	0.13	-	-	f, g
1331	1329	7*H*-α-Silphiperfol-5-ene	SH	tr	0.08	tr	0.08	f, g
1338	1334	Presilphiperfol-7-ene	SH	-	-	0.1	0.25	f, g
1339	1344	*exo*-2-Hydroxycineole acetate	MO	-	-	tr	0.12	f, g
1350	1352	7*H*-β-Silphiperfol-5-ene	SH	0.1	0.27	0.3	0.76	f, g
1353	1350	α-Longipinene	SH	-	-	tr	0.08	f, g
1359	1356	Eugenol	SM	tr	0.11	tr	0.10	f, g, h
1364	1364	Decanoic acid	FAD	-	-	tr	0.12	f, g, h
1365	1359	Neryl acetate	MO	tr	0.13	tr	0.12	f, g
1374	1373	Linalyl isobutyrate	MO	tr	0.13	tr	0.12	f, g
1377	1374	α-Copaene	SH	-	-	tr	0.08	f, g
1381	1377	Silphiperfol-6-ene	SH	-	-	0.5	1.27	f, g
1381	1383	(*E*)-β-Damascenone	C	-	-	tr	0.10	f, g
1387	1382	Modheph-2-ene	SH	tr	0.11	0.1	0.25	f, g
1390	1391	Octyl butanoate	FAD	0.2	0.84	-	-	f, g
1390	1387	β-Bourbonene	SH	-	-	tr	0.08	f, g
1394	1390	7-*epi*-Sesquithujene	SH	tr	0.08	0.2	0.51	f, g
1395	1387	α-Isocomene	SH	-	-	tr	-	f, g
1396	1389	β-Elemene	SH	tr	0.08	0.3	0.76	f, g
1395	1392	(*Z*)-Jasmone	C	tr	0.11	-	-	f, g
1400	1400	Tetradecane	FAD	-	-	tr	0.07	f, g, h
1403	1398	Petasitene	SH	-	-	tr	0.08	f, g
1406	1403	Methyl eugenol	SM	0.1	0.43	-	-	f, g
1411	1405	Italicene	SH	0.2	0.55	0.3	0.76	f, g
1412	1407	β-Isocomene	SH	-	-	0.1	0.25	f, g
1412	1408	(*Z*)-Caryophyllene	SH	-	-	tr	0.08	f, g
1415	1411	*cis*-α-Bergamotene	SH	-	-	tr	0.08	f, g
1420	1422	Bornyl isobutyrate	MO	0.1	0.42	0.6	2.34	f, g
1421	1424	7,8-Dihydro-3,4-dehydro-β-ionone	C	-	-	tr	0.10	f, g
1426	1424	2,5-Dimethoxy-*p*-cymene	MO	-	-	tr	0.12	f, g
1428	1417	(*E*)-Caryophyllene	SH	5.6	15.35	8.2	20.75	f, g, h
1436	1430	β-Copaene	SH	-	-	tr	0.08	f, g
1436	1430	Neryl acetone	C	tr	0.11	tr	0.10	f, g
1443	1432	*trans*-α-Bergamotene	SH	tr	0.08	tr	0.08	f, g
1447	1446	Sesquisabinene B	SH	-	-	tr	0.08	f, g
1447	1440	(*Z*)-β-Farnesene	SH	0.4	1.10	1.0	2.53	f, g
1454	1453	Geranyl acetone	C	0.1	0.36	0.1	0.33	f, g
1461	1452	α-Humulene	SH	0.3	0.82	0.5	1.27	f, g
1463	1467	2-Methyltetradecane	FAD	-	-	tr	0.07	f, g
1465	1458	*allo*-Aromadendrene	SH	-	-	tr	0.08	f, g
1470	1464	α-Acoradiene	SH	tr	0.08	tr	0.08	f, g
1473	1474	10-*epi*-β-Acoradiene	SH	tr	0.08	tr	0.08	f, g
1474	1469	1-Dodecanol	FAD	-	-	0.2	0.65	f, g
1475	1471	4,5-di-*epi*-Aristolochene	SH	tr	0.08	-	-	f, g
1483	1481	γ-Curcumene	SH	0.8	2.19	1.2	3.04	f, g
1486	1479	*ar*-Curcumene	SH	0.5	1.37	1.0	2.53	f, g
1486	1484	Germacrene D	SH	tr	0.08	-	-	f, g, h
1488	1487	(*E*)-β-Ionone	C	-	-	tr	0.10	f, g
1487	1480	Thymyl isobutyrate	MO	tr	0.13	tr	0.12	f, g
1493	1490	Neryl isobutyrate	MO	22.1	93.25	16.4	63.87	f, g
1494	1493	*trans*-Muurola-4(14),5-diene	SH	tr	0.08	-	-	f, g
1496	1498	Eremophilene	SH	tr	0.08	-	-	f, g
1496	1496	Viridiflorene	SH	tr	0.08	-	-	f, g
1499	/	6-Methoxythymyl acetate *	MO	-	-	tr	0.12	f
1504	1498	α-Selinene	SH	tr	0.08	-	-	f, g
1506	1500	α-Muurolene	SH	-	-	tr	0.08	f, g
1513	1515	β-Bisabolene	SH	0.6	1.64	2.1	5.31	f, g
1514	1507	7-*epi*-Eremophila-1(10),8,11-triene	SH	-	-	-	-	f, g
1516	1514	β-Curcumene	SH	0.4	1.10	0.2	0.51	f, g
1519	1510	Cameroonan-7α-ol	SO	-	-	tr	0.10	f, g
1520	1513	γ-Cadinene	SH	-	-	tr	0.08	f, g
1523	1514	Cubebol	SO	-	-	tr	0.10	f, g
1523	1515	10-*epi*-Italicene ether	SO	-	-	tr	0.12	f, g
1523	1511	3,4-Dimethyl-5-pentyl-2(5*H*)-furanone	FAD	-	-	tr	0.10	f, g
1524	1515	Sesquicineole	SO	tr	0.11	-	-	f, g
1528	1519	Silphiperfolan-7β-ol	SO	-	-	0.1	0.32	f, g
1528	1521	Bornyl isovalerate	MO	-	-	tr	0.12	f, g
1529	1522	δ-Cadinene	SH	0.2	0.55	0.3	0.76	f, g
1530	1523	*cis*-Bovolide	FAD	-	-	tr	0.08	f, g
1532	1531	(*Z*)-Nerolidol	SO	-	-	tr	0.10	f, g
1533	1528	*cis*-Calamenene	SH	tr	0.08	-	-	f, g
1535	1529	Kessane	SO	0.1	0.27	0.3	0.76	f, g
1541	1536	Italicene ether	SO	0.1	0.43	tr	0.12	f, g
1541	1537	α-Cadinene	SH	tr	0.08	-	-	f, g
1538	1534	Liguloxide	SO	tr	0.13	-	-	f, g
1548	1542	*cis*-Sesquisabinene hydrate	SO	0.1	0.35	0.2	0.65	f, g
1550	1544	α-Calacorene	SH	tr	0.08	-	-	f, g
1552	1547	Italicene epoxide	SO	tr	0.13	-	-	f, g
1553		Unidentified constituent^j^		0.4	-	0.1	-	
1559	1551	7-*epi*-*trans*-Sesquisabinene hydrate	SO	-	-	tr	0.10	f, g
1560	1555	Elemicin	SM	0.1	0.35	-	-	f, g
1561	1564	Isocaryophyllene oxide	SO	0.2	0.86	0.4	1.59	f, g
1566	1561	(*E*)-Nerolidol	SO	tr	0.11	-	-	f, g
1569	1567	Longipinanol	SO	0.7	2.46	1.1	3.57	f, g
1574	1565	(*Z*)-3-Hexenyl benzoate	FAD	tr	0.13	-	-	f, g
1579	1582	Neryl 2-methylbutanoate	MO	5.5	23.21	4.5	17.53	f, g
1580	1577	Spathulenol	SO	0.6	2.11	-	-	f, g
1581	1577	*trans*-Sesquisabinene hydrate	SO	-	-	1.7	5.52	f, g
1586	1582	Neryl isovalerate	MO	1.4	5.91	0.7	2.73	f, g
1592	1582	Caryophyllene oxide	SO	3.7	15.91	3.7	14.69	f, g
1593	1585	Presilphiperfolan-8-ol	SO	-	-	tr	0.10	f, g
1596	1590	Globulol	SO	tr	0.11	-	-	f, g
1600	1596	Fokienol	SO	0.2	0.70	0.3	0.97	f, g
1602	1599	4(14)-Salvialene-1-one	SO	tr	0.11	-	-	f, g
1606	1592	Viridiflorol	SO	tr	0.11	-	-	f, g
1615	1608	Humulene epoxide II	SO	0.6	2.58	0.3	1.19	f, g
1618	1611	Tetradecanal	FAD	tr	0.11	-	-	f, g
1618	1620	Humulene epoxide III	SO	0.3	0.82	-	-	f, g
1626	1613	*epi*-Marsupellol	SO	0.4	1.41	0.6	1.95	f, g
1633	1627	1-*epi*-Cubenol	SO	tr	0.11	-	-	f, g
1644	1639	Caryophylla-3(15),7(14)-dien-6α-ol	SO	0.3	1.06	0.4	1.30	f, g
1646	1632	Eudesm-3,11-dien-5-ol	SO	tr	0.11	-	-	f, g
1648	1638	*epi*-α-Cadinol	SO	-	-	tr	0.10	f, g
1649	1639	Caryophylla-3(15),7(14)-dien-6β-ol	SO	1.0	3.52	1.3	4.22	f, g
1655	1643	13-Tetradecanolide	FAD	-	-	tr	0.08	f, g
1658	1668	Bicyclohumulenone	SO	0.1	0.36	-	-	f, g
1662	1652	α-Cadinol	SO	0.3	1.06	0.4	1.30	f, g
1664	1656	(*Z*)-Caryophylla-3(15),6-dien-14-ol (syn. 14-hydroxy-(*Z*)-caryophyllene)	SO	0.4	1.41	1.2	3.90	f, g
1667	1658	*neo*-Intermedeol	SO	tr	0.11	-	-	f, g
1667	1658	11-Selinen-4α-ol	SO	tr	0.11	tr	0.10	f, g
1672	1665	Intermedeol	SO	-	-	tr	0.10	f, g
1674	1675	(*E*)-*trans*-α-Bergamota-2,10-dien-12-al	SO	tr	0.11	tr	0.11	f, g
1678	1670	*epi*-β-Bisabolol	SO	-	-	tr	0.10	f, g
1679	1674	β-Bisabolol	SO	-	-	tr	0.10	f, g
1679	1668	(*E*)-2-*epi*-Caryophylla-3(15),6-dien-14-ol (syn. 14-hydroxy-9-*epi*-(*E*)-caryophyllene)	SO	0.8	2.82	1.4	4.55	f, g
1680	1693	β-Sinensal	SO	0.2	0.76	-	-	f, g
1685	1685	Germacra-4(15),5,10(14)-trien-1α-ol	SO	0.2	0.70	-	-	f, g
1689	1658	6-Methoxythymyl isobutyrate	MO	0.2	0.84	0.3	1.17	f, g
1690	1690	(*Z*)-α-*trans*-Bergamotol	SO	0.6	2.11	0.6	1.95	f, g
1697	1688	Shyobunol	SO	-	-	tr	0.10	f, g
1698	1704	Bicyclogermacren-14-al	SO	1.0	3.82	-	-	f, g
1700	1700	Heptadecane	FAD	tr	0.08	-	-	f, g, h
1701	1708	Italicen-13-al	SO	-	-	0.3	1.06	f, g
1709	/	6-(Isobutyryloxy)thymyl methyl ether *	MO	0.3	1.29	0.3	1.19	f
1709	1700	Amorpha-4,9-dien-2-ol	SO	-	-	tr	0.10	f, g
1712	1715	Pentadecanal	FAD	-	-	0.3	1.06	f, g
1713	1712	*ar*-Curcumen-15-al	SO	-	-	0.1	0.35	f, g
1725	1723	3-Methoxycuminyl isobutyrate	MO	31.1	131.22	25.5	99.32	f, g, h
1732	1730	(*E*,*E*)-Farnesal	SO	0.2	0.76	0.5	1.76	f, g
1734	1733	(*E*)-γ-Curcumen-12-ol	SO	-	-	tr	0.10	f, g
1734	1724	(*Z*)-Nuciferol	SO	tr	0.11	tr	0.10	f, g
1741	1732	Zerumbone	SO	0.2	0.71	0.3	0.99	f, g
1750	1740	Mint sulfide	O	tr	0.08	-	-	f, g
1751	1754	(*Z*)-β-Curcumen-12-ol	SO	0.1	0.35	0.2	0.65	f, g
1762	1762	β-Acoradienol	SO	0.1	0.35	0.3	0.97	f, g
1764	1762	Tetradecanoic acid	FAD	0.1	0.42	tr	0.12	f, g, h
1766	1760	(*Z*)-Lanceol	SO	0.1	0.35	0.1	0.32	f, g
1767	1759	Benzyl benzoate	FAD	tr	0.13	tr	0.12	f, g
1775	1768	β-Bisabolenal	SO	0.2	0.76	0.5	1.76	f, g
1776	1765	10-*epi*-Italicen-12-yl acetate	SO	tr	0.13	0.2	0.78	f, g
1786	1784	Phenanthrene	O	-	-	tr	0.08	f, g
1791	1789	β-Bisabolenol	SO	-	-	0.5	1.62	f, g
1794	1796	Eudesma-3,11-dien-2-one	SO	0.1	0.36	0.4	1.32	f, g
1798	1780	Italicen-12-yl acetate	SO	-	-	tr	0.12	f, g
1800	1800	Octadecane	FAD	tr	0.08	-	-	f, g, h
1808	-	3-Methoxycuminyl 2-methylbutyrate	MO	1.3	5.49	1.7	6.62	f, g, h
1817	-	3-Methoxycuminyl 3-methylbutyrate	MO	0.1	0.42	0.1	0.39	f, g, h
1820	1818	Hexadecanal	FAD	tr	0.11	-	-	f, g
1833	1820	(*Z*)-γ-Curcumen-12-yl acetate	SO	-	-	0.7	2.73	f, g
1836	1830	(*Z*)-Nuciferyl acetate	SO	0.1	0.42	0.5	1.95	f, g
1834	1832	15-Pentadecanolide	FAD	tr	0.08	-	-	f, g
1840	1845	(2*E*,6*E*)-Farnesyl acetate	SO	-	-	tr	0.12	f, g
1847	1845	Hexahydrofarnesyl acetone	C	0.4	1.43	0.4	1.32	f, g
1859	1848	15-Hexadecanolide	FAD	0.2	0.55	0.1	0.25	f, g
1862	1854	(*Z*)-Lanceyl acetate	SO	-	-	tr	0.12	f, g
1876	1864	Benzyl salicylate	SM	-	-	tr	0.12	f, g
1887	1889	(5*Z*,9*E*)-Farnesyl acetone	C	-	-	tr	0.10	f, g
1897	1896	(8*Z*,11*Z*,14*Z*)-8,11,14-Heptadecatrienal	FAD	-	-	tr	0.11	f, g
1900	1900	Nonadecane	FAD	-	-	tr	0.07	f, g, h
1913	1913	(5*E*,9*E*)-Farnesyl acetone	C	tr	0.11	-	-	f, g
1915	1924	3-(Isobutyryloxy)-4-isopropylbenzyl isobutyrate	MO	0.3	1.27	0.4	1.56	f, g
1924	1920	Heptadecanal	FAD	tr	0.11	-	-	f, g
1930	1921	Methyl hexadecanoate	FAD	-	-	tr	0.12	f, g
1951	1934	(*Z*)-γ-Curcumen-12-yl isobutyrate	SO	0.1	0.42	0.1	0.39	f, g
1955	1945	(*Z*)-Nuciferyl isobutyrate	SO	0.4	1.69	0.4	1.56	f, g
1959	1959	Hexadecanoic acid	FAD	0.3	1.27	0.5	1.95	f, g, h
2000	2000	Icosane	FAD	-	-	tr	0.07	f, g, h
2015	2009	13-*epi*-Manool oxide	DO	0.7	3.01	0.4	1.59	f, g
2028	2036	10-Isobutyryloxy-8,9-epoxythymyl isobutyrate	MO	2.2	9.28	1.5	5.84	f, g, i
2039	2025	(*Z*)-γ-Curcumen-12-yl isovalerate	SO	tr	0.13	0.1	0.39	f, g
2042	2025	(*Z*)-Nuciferyl isovalerate	SO	-	-	0.1	0.39	f, g
2089	2083	1-Octadecanol	FAD	-	-	tr	0.10	f, g, h
2091	2090	1-Heneicosene	FAD	-	-	tr	0.08	f, g
2100	2100	Heneicosane	FAD	tr	0.08	tr	0.07	f, g, h
2106	2106	5-Dodecyldihydro-2(3*H*)-furanone	FAD	-	-	tr	0.10	f, g
2116	2122	*cis*-Phytol	DO	0.1	0.35	0.5	1.62	f, g
2117	-	10-(2-Methylbutyryloxy)-8,9-epoxythymyl isobutyrate *	MO	0.5	2.11	0.5	1.95	f
2120	2122	10-Isovaleryloxy-8,9-epoxythymyl isobutyrate	MO	0.1	0.42	0.1	0.39	f, g, i
2146	2143	(9*Z*,12*Z*,15*Z*)-9,12,15-Octadecatrienoic acid	FAD	-	-	tr	0.12	f, g
2200	2200	Docosane	FAD	-	-	tr	0.07	f, g, h
2227	2218	*cis*-Phytyl acetate	DO	-	-	tr	0.12	f, g
2232	2224	Eicosanal	FAD	-	-	tr	0.11	f, g, h
2299	2296	1-Eicosanol	FAD	-	-	tr	0.10	f, g
2300	2300	Tricosane	FAD	0.1	0.27	tr	0.07	f, g, h
2400	2400	Tetracosane	FAD	tr	0.08	tr	0.07	f, g, h
2500	2500	Pentacosane	FAD	1.1	2.96	tr	0.07	f, g, h
2600	2600	Hexacosane	FAD	0.1	0.27	tr	0.07	f, g, h
2700	2700	Heptacosane	FAD	0.3	0.81	0.9	2.23	f, g, h
2800	2800	Octacosane	FAD	tr	0.08	tr	0.07	f, g, h
2900	2900	Nonacosane	FAD	0.1	0.27	tr	0.07	f, g, h
3000	3000	Triacontane	FAD	tr	0.08	tr	0.07	f, g, h
3100	3100	Hentriacontane	FAD	tr	0.08	tr	0.07	f, g, h
		Total identified [%]		96.6		94.7		
		Carotenoid derivatives	C	0.5		0.5		
		Diterpenoids	DO	0.8		0.9		
		Fatty acid and fatty acid-related compounds	FAD	4.4		2.4		
		Monoterpene hydrocarbons	MH	tr		tr		
		Oxygenated monoterpenes	MO	68.0		55.2		
		Oxygenated sesquiterpenes	SO	13.5		19.8		
		Others	O	tr		tr		
		Sesquiterpene hydrocarbons	SH	9.1		16.4		
		Shikimate metabolites	SM	0.2		tr		

^a^ RI = retention indices; Exp = determined relative to a homologous series of n-alkanes (C_7_–C_31_) on a DB-5MS column; Lit = literature retention index values taken from Adams [8] and NIST 17 [9];/There is no available literature RI data for the identified essential-oil constituent. ^b^ *syn*. = synonym.^c^ C = Class; for compound class abbreviations, cf. last rows of this Table. ^d^ The essential oil of *P. dysenterica* aerial parts collected in the village Skrapež (2012; sample **A**) and urban settings of the city of Niš (2010; sample **B**); %: tr = trace amounts (<0.05%); - = not detected; *c*: mean value of concentration in mg per 100 g of dry plant material. ^e^ ID = identification method; f = constituent identified by mass-spectra comparison with those listed in Wiley 11, NIST17 [9], MassFinder 2.3, and a homemade mass spectral library; g = constituent identified by retention index matching with literature data; h = constituent identity confirmed by GC co-injection of an authentic sample; i = structure of the identified essential-oil constituent was confirmed by 1D and 2D NMR analysis. ^j^ Unidentified constituent: MS (EI), *m*/*z* (%) 220(17) [M^+^], 205(5), 177(2), 150(11), 133(100), 131(4), 118(15), 117(32), 115(13), 107(16), 105(21), 103(5), 91(19), 79(5), 77(7), 71(11), 65(3), 51(2), 43(20), 41(10). * Tentatively identified essential-oil constituent by analysis of mass fragmentation and the prediction of retention index.

**Table 2:** ^1^H and ^13^C NMR spectroscopic data (CDCl_3_; 400 and 100.6 MHz, respectively) for 3-methoxycuminyl isobutanoate (**7**), 3-methoxycuminyl 2-methylbutanoate (**9**), and 3-methoxycuminyl isovalerate (**10**).

Position	Compound					
	7		9		10	
	^1^H	^13^C	^1^H	^13^C	^1^H	^13^C
1	-	134.6	-	134.7	-	134.5
2	6.82 (d, *J* = 1.5 Hz, 1H)	110.0	6.82 (d, *J* = 1.5 Hz, 1H)	110.1	6.83 (d, *J* = 1.5 Hz, 1H)	110.3
3	-	156.8	-	156.8	-	156.8
4	-	137.0	-	137.0	-	137.1
5	7.19 (d, *J* = 7.7 Hz, 1H)	126.1	7.19 (d, *J* = 7.7 Hz, 1H)	126.1	7.19 (d, *J* = 7.7 Hz, 1H)	126.1
6	6.91 (dd, *J* = 7.7, 1.5 Hz, 1H)	120.2	6.91 (dd, *J* = 7.7, 1.5 Hz, 1H)	120.3	6.92 (dd, *J* = 7.7, 1.5 Hz, 1H)	120.5
7	5.08 (s, 2H)	66.2	5.09 (s, 2H)	66.1	5.08 (s, 2H)	66.2
8	3.30 (sept, *J* = 6.9 Hz, 1H)	26.6	3.30 (sept, *J* = 6.9 Hz, 1H)	26.6	3.30 (sept, *J* = 6.9 Hz, 1H)	26.6
9 and 10	1.20 (d, *J* = 6.9 Hz, 6H)	22.6	1.20 (d, *J* = 6.9 Hz, 6H)	22.6	1.20 (d, *J* = 6.9 Hz, 6H)	22.6
11	3.83 (s, 3H)	55.3	3.83 (s, 3H)	55.3	3.83 (s, 3H)	55.3
12	-	177.1	-	176.6	-	173.0
13	2.60 (sept, *J* = 7.0 Hz, 1H)	34.0	2.43 ^a^ (pseudo sext, *J* = 7.17, 7.0, 6.65 Hz, 1H)	41.1	2.24 (d, *J* = 6.9 Hz, 2H)	43.5
14	1.20 (d, *J* = 7.0 Hz, 6H)	19.0	1.7119 ^a^ (dqd, *J* = −13.65, 7.40, 7.17 Hz, 1H); 1.4950 ^a^ (dqd, −13.65, 7.41, 6.65 Hz, 1H)	26.8	2.14 (tsept, *J* = 6.9, 6.6 Hz, 1H)	25.8
15			0.91 ^a^ (pseudo t, *J* = 7.41, 7.40 Hz, 3H)	11.6	0.96 (d, *J* = 6.6 Hz, 6H)	22.4
16			1.17 (d, *J* = 7.0 Hz, 3H)	16.6		

^a^ The presented values of chemical shifts and coupling constants, including their sign (Supplementary Materials Figure S34), were determined from manual iterative total spin ^1^H NMR simulation [12].

**Table 3:** Acetylcholinesterase inhibitory activity of *P. dysenterica* essential oil, cuminal (**1**), and synthesized compounds (**2–11**).

Compound	Code	% of AChE Inhibition ^a,b^
Cuminaldehyde	**1**	12.8
3-Nitrocuminaldehyde	**2**	47.5
3-Nitrocuminol	**3**	40.4
3-Aminocuminol	**4**	11.7
3-Hydroxycuminol	**5**	26.2
3-Methoxycuminol	**6**	32.1
3-Methoxycuminyl isobutanoate	**7**	<5
3-Methoxycuminyl butanoate	**8**	<5
3-Methoxycuminyl 2-methylbutanoate	**9**	<5
3-Methoxycuminyl 3-methylbutanoate	**10**	<5
3-Methoxycuminyl pentanoate	**11**	<5
*Pulicaria dysenterica* essential oil	**EO**	14.9

^a^ When applied in the highest tested concentration (0.5 mmol/L (**1–11**) or 125 mg/L in the case of the **EO** sample). ^b^ IC_50_ (µmol/L) was not determined as higher concentrations of the **EO** or the synthesized compounds were not accessible due to their low solubility in a 10% aqueous methanol solution.

**Table 4:** Antimicrobial activity of *P. dysenterica* essential oil and pure synthesized compounds against ATTC strains of bacteria and fungi.

Sample	Strains									
	Gram-Positive				Gram-Negative				Fungi	
	*S. aureus*	*B. cereus*	*K. rhizophila*	*S. epidermidis*	*P. aeruginosa*	*E. coli*	*A. baumanii*	*S. enterica*	*C. albicans*	*A. brasiliensis*
**EO** ^a^	0.12	0.12	0.12	0.12	4.00	2.00	1.00	1.00	0.50	1.00
**2** ^b^	0.31	0.31	0.31	0.62	20.73	5.18	2.59	2.59	0.31	1.30
**3** ^b^	2.56	2.56	1.28	2.56	2.56	2.56	2.56	2.56	1.28	1.28
**4** ^b^	12.12	12.12	6.06	12.12	24.24	24.24	6.06	12.12	12.12	12.12
**5** ^b^	0.18	0.18	0.06	0.06	12.05	3.01	1.51	3.01	3.01	12.05
**6** ^b^	2.78	0.67	0.33	2.78	1.39	2.78	2.78	2.78	1.39	1.39
**7** ^b^	16.00	16.00	8.00	16.00	16.00	16.00	8.00	16.00	8.00	8.00
**8** ^b^	8.00	8.00	8.00	8.00	8.00	8.00	4.00	8.00	4.00	4.00
**9** ^b^	15.15	15.15	7.58	15.15	15.15	15.15	7.58	15.15	7.58	7.58
**10** ^b^	>15.15	15.15	15.15	15.15	15.15	15.15	7.58	15.15	7.58	3.79
**11** ^b^	15.15	15.15	7.58	>15.15	15.15	15.15	7.58	15.15	7.58	7.58
**CHL** ^c^	10.76	5.37	1.34	2.69	21.51	2.69	43.03	21.51	- ^d^	- ^d^
**STR** ^c^	1.21	0.28	4.83	4.83	9.66	1.21	38.70	9.66	- ^d^	- ^d^
**NYS** ^c^	- ^d^	- ^d^	- ^d^	- ^d^	- ^d^	- ^d^	- ^d^	- ^d^	2.53	0.32

^a^ mg/mL; ^b^ mmol/L; ^c^ µmol/L; ^d^/= not tested; CHL (chloramphenicol), STR (streptomycin), and NYS (nystatin) served as positive controls.

**Table 5:** Antimicrobial activity of *P. dysenterica* essential oil and pure synthesized compounds against human isolates of *Salmonella* spp.

Sample	*Salmonella* spp. Isolates from Stool								
	S1	S2	S3	S4	S5	S6	S7	S8	ATCC
**EO** ^a^	0.50	0.50	2.00	0.50	0.50	0.50	0.50	0.12	1.00
**2** ^b^	2.59	2.59	2.59	1.30	2.59	0.62	1.30	2.59	2.59
**3** ^b^	1.28	2.56	2.56	2.56	2.56	2.56	2.56	2.56	2.56
**4** ^b^	12.12	12.12	3.03	12.12	12.12	6.06	12.12	12.12	12.12
**5** ^b^	0.72	3.01	1.51	1.51	1.51	0.72	3.01	1.51	3.01
**6** ^b^	1.39	2.78	5.56	2.78	2.78	2.78	2.78	2.78	2.78
**7** ^b^	16.00	16.00	16.00	8.00	8.00	16.00	16.00	16.00	16.00
**8** ^b^	8.00	8.00	8.00	2.00	2.00	8.00	8.00	8.00	8.00
**9** ^b^	15.15	15.15	15.15	15.15	15.15	15.15	15.15	7.58	15.15
**10** ^b^	15.15	15.15	15.15	15.15	15.15	15.15	15.15	7.58	15.15
**11** ^b^	15.15	7.58	7.58	7.58	7.58	3.79	15.15	15.15	15.15

^a^ mg/mL; ^b^ mmol/L.

**Table 6:** Macrophage viability estimated using an MTT assay following incubation with different concentrations of **EO** and selected pure compounds.

Sample		Concentration (mol/L)				
		10^−4^	10^−5^	10^−6^	10^−7^	10^−8^
**EO** ^a^	Mean	40.1 *	88.1 **	95.8	98.6	99.1
	SD	0.7	6.5	10.9	5.8	2.5
**2**	Mean	46.7 *	106.1	105.6	107.1	108.6
	SD	3.3	13.4	8.7	14.5	0.4
**3**	Mean	99.4	107.4	108.2	111.2	106.4
	SD	1.1	8.0	14.0	7.3	9.1
**4**	Mean	91.4	110.2	104.5	95.0	107.9
	SD	18.9	5.4	13.4	13.1	4.0
**5**	Mean	46.2 *	114.9	108.1	109.4	108.4
	SD	2.9	8.7	1.5	15.6	7.3
**6**	Mean	70.4 *	96.1	105.6	108.4	107.1
	SD	5.1	4.0	8.7	7.2	5.1
**7**	Mean	97.1	111.5	100.4	99.9	104.8
	SD	4.7	14.9	8.4	5.8	12.4
**9**	Mean	79.6 *	106.6	115.4	113.9	96.6
	SD	1.5	5.1	13.7	14.2	12.7
**10**	Mean	75.8 *	106.6	96.1	103.3	112.6
	SD	9.1	5.1	10.2	2.2	8.7
**CP**	Mean			54.3 *		
	SD			8.2		
**RPMI**	Mean	100				
	SD	5.3				

** *p* < 0.05; * *p* < 0.001 vs. RPMI; ^a^ concentrations of the essential oil were 100, 10, 1, 0.1, and 0.01 mg/L.

## Data Availability

Not applicable.

## Supplementary Materials

The following supporting information can be downloaded at: www.mdpi.com/article/10.3390/plants11233340/s1, Figures S1–S34.
