# Supplementary material for: Pulicaria dysenterica (L.) Bernh.—Rightfully Earned Name? Identification and Biological Activity of New 3-Methoxycuminyl Esters from P. dysenterica Essential Oil
Source: Plants (Basel). 2022 Dec 1;11(23):3340. doi: 10.3390/plants11233340 (PMC9739903; doi:10.3390/plants11233340)
Supplement: Supplementary file 1 [file plants-11-03340-s001.zip › plants-2048338-supplementary.pdf]

Supplementary material

for

Pulicaria dysenterica (L.) Bernh.—Rightfully Earned Name? Identification and Biological Activity of New 3-Methoxycuminyl Esters from *P. dysenterica* Essential Oil

Niko S. Radulović [1,*], Marko Z. Mladenović [1], Dušan R. Vukićević [2], Nikola M. Stojanović [3], Pavle J. Randjelović [3], Zorica Z. Stojanović-Radić [4] and Fabio Boylan [5]

[1] Department of Chemistry, Faculty of Sciences and Mathematics, University of Nis, Višegradska 33, 18000 Nis, Serbia
[2] Faculty of Medicine, University of Kragujevac, Svetozara Markovića 69, 34000 Kragujevac, Serbia
[3] Department of Physiology, Faculty of Medicine, University of Nis, Zorana Đinđica 81, 18000 Nis, Serbia
[4] Department of Biology and Ecology, Faculty of Sciences and Mathematics, University of Nis, Visegradska 33, 18000 Nis, Serbia
[5] School of Pharmacy and Pharmaceutical Sciences, Panoz Institute, Trinity College, Westland Row, 2 Dublin, Ireland
* Correspondence: nikoradulovic@yahoo.com; Tel.: +381-18-533-015; Fax: +381-18-533-014

Content:

Figure S1. Mass spectrum of 3-methoxycuminyl isobutanoate (**7**).
Figure S2. ^{1}H NMR spectrum of 3-methoxycuminyl isobutanoate (**7**).
Figure S3. ^{13}C NMR spectrum of 3-methoxycuminyl isobutanoate (**7**).
Figure S4. ^{13}C NMR spectrum (without heteronuclear decoupling) of 3-methoxycuminyl isobutanoate (**7**).
Figure S5. Distortionless enhancement by polarization transfer spectrum (DEPT90) of 3-methoxycuminyl isobutanoate (**7**).
Figure S6. Distortionless enhancement by polarization transfer spectrum (DEPT135) of 3-methoxycuminyl isobutanoate (**7**).
Figure S7. Gradient ^{1}H–^{1}H COSY spectrum of 3-methoxycuminyl isobutanoate (**7**).
Figure S8. Gradient HSQC spectrum of 3-methoxycuminyl isobutanoate (**7**).
Figure S9. Gradient HMBC spectrum of 3-methoxycuminyl isobutanoate (**7**).
Figure S10. Mass spectrum of 3-methoxycuminyl butanoate (**8**).
Figure S11. ^{1}H NMR spectrum of 3-methoxycuminyl butanoate (**8**).
Figure S12. ^{13}C NMR spectrum of 3-methoxycuminyl butanoate (**8**).
Figure S13. Mass spectrum of 3-methoxycuminyl 2-methylbutanoate (**9**).
Figure S14. ^{1}H NMR spectrum of 3-methoxycuminyl 2-methylbutanoate (**9**).
Figure S15. ^{13}C NMR spectrum of 3-methoxycuminyl 2-methylbutanoate (**9**).

Figure S16. ^{13}C NMR spectrum (without heteronuclear decoupling) of 3-methoxycuminyl 2-methylbutanoate (**9**).
Figure S17. Distortionless enhancement by polarization transfer spectrum (DEPT90) of 3-methoxycuminyl 2-methylbutanoate (**9**).
Figure S18. Distortionless enhancement by polarization transfer spectrum (DEPT135) of 3-methoxycuminyl 2-methylbutanoate (**9**).
Figure S19. Gradient ^{1}H–^{1}H COSY spectrum of 3-methoxycuminyl 2-methylbutanoate (**9**).
Figure S20. Gradient HSQC spectrum of 3-methoxycuminyl 2-methylbutanoate (**9**).
Figure S21. Gradient HMBC spectrum of 3-methoxycuminyl 2-methylbutanoate (**9**).
Figure S22. Mass spectrum of 3-methoxycuminyl 3-methylbutanoate (**10**).
Figure S23. ^{1}H NMR spectrum of 3-methoxycuminyl 3-methylbutanoate (**10**).
Figure S24. ^{13}C NMR spectrum of 3-methoxycuminyl 3-methylbutanoate (**10**).
Figure S25. ^{13}C NMR spectrum (without heteronuclear decoupling) of 3-methoxycuminyl 3-methylbutanoate (**10**).
Figure S26. Distortionless enhancement by polarization transfer spectrum (DEPT90) of 3-methoxycuminyl 3-methylbutanoate (**10**).
Figure S27. Distortionless enhancement by polarization transfer spectrum (DEPT135) of 3-methoxycuminyl 3-methylbutanoate (**10**).
Figure S28. Gradient ^{1}H–^{1}H COSY spectrum of 3-methoxycuminyl 3-methylbutanoate (**10**).
Figure S29. Gradient HSQC spectrum of 3-methoxycuminyl 3-methylbutanoate (**10**).
Figure S30. Gradient HMBC spectrum of 3-methoxycuminyl 3-methylbutanoate (**10**).
Figure S31. Mass spectrum of 3-methoxycuminyl pentanoate (**11**).
Figure S32. ^{1}H NMR spectrum of 3-methoxycuminyl pentanoate (**11**).
Figure S33. ^{13}C NMR spectrum of 3-methoxycuminyl pentanoate (**11**).
Figure S34. Spin simulation of the ^{1}H NMR signals for the protons at position 13 and 14 (lower) and comparison with the experimental ones (upper) for 3-methoxycuminyl 2-methylbutanoate (**9**).

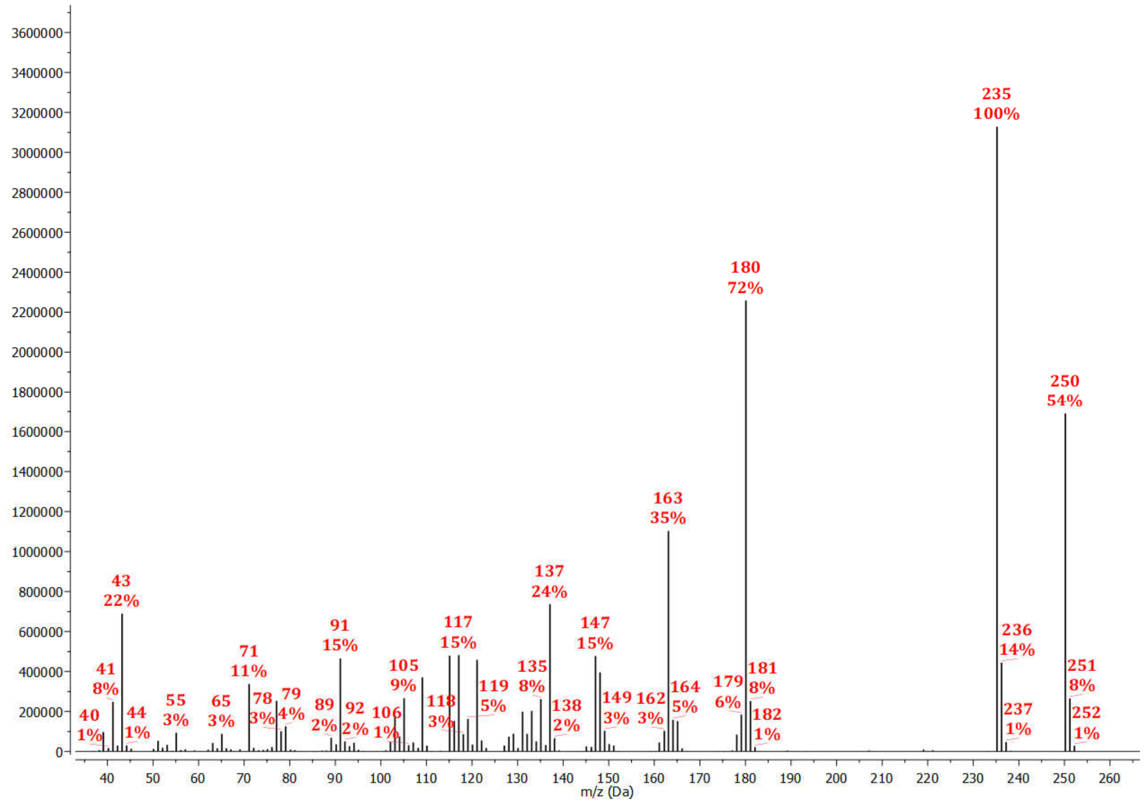

Figure S1. Mass spectrum of 3-methoxycuminyl isobutanoate (**7**).

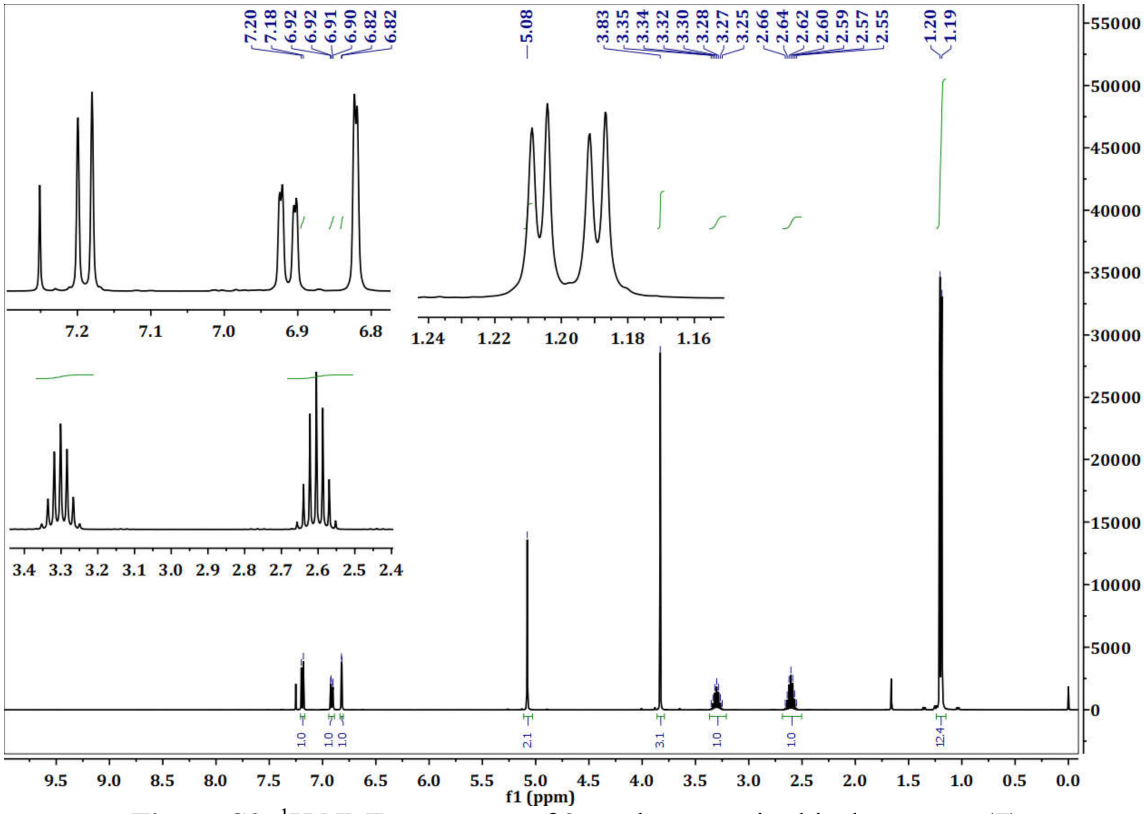

Figure S2. ^{1}H NMR spectrum of 3-methoxycuminyl isobutanoate (**7**).

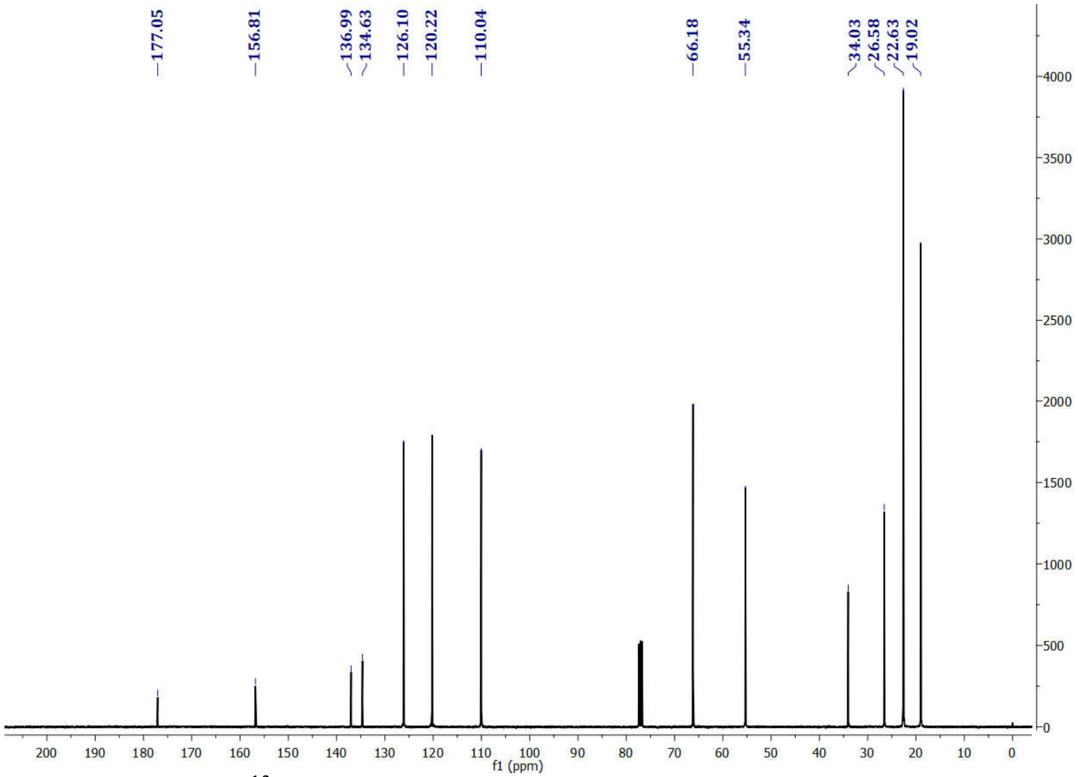

Figure S3. ^{13}C NMR spectrum of 3-methoxycuminyl isobutanoate (**7**).

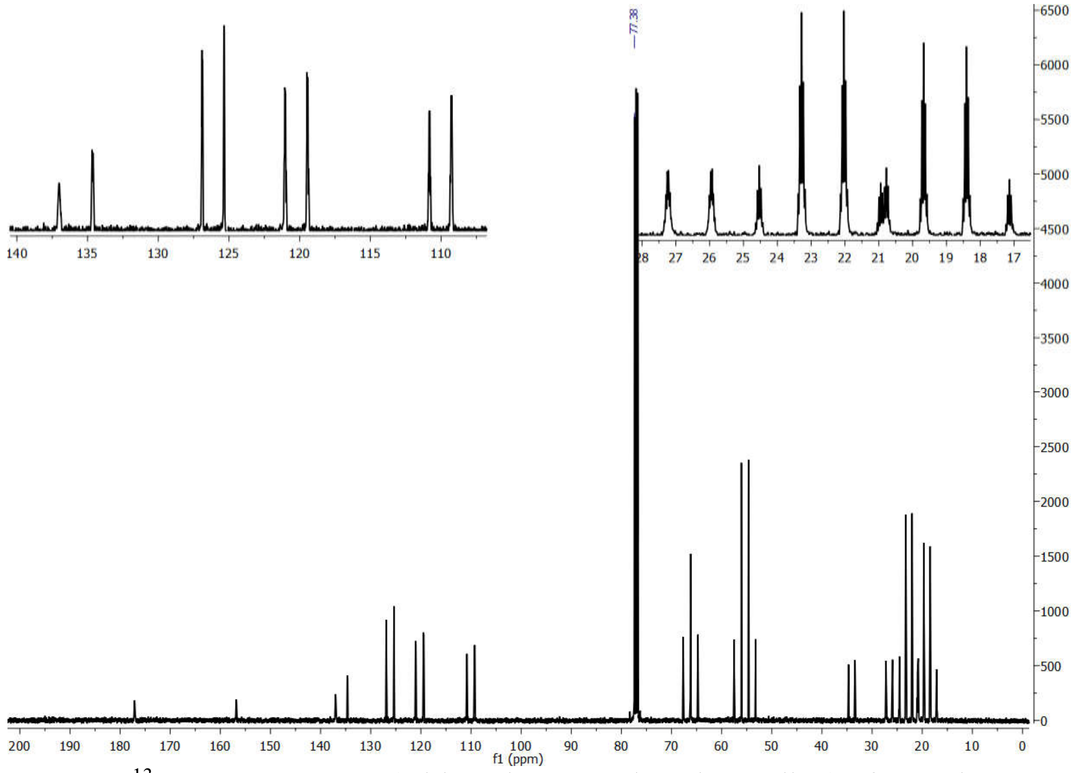

Figure S4. ^{13}C NMR spectrum (without heteronuclear decoupling) of 3-methoxycuminyl isobutanoate (**7**).

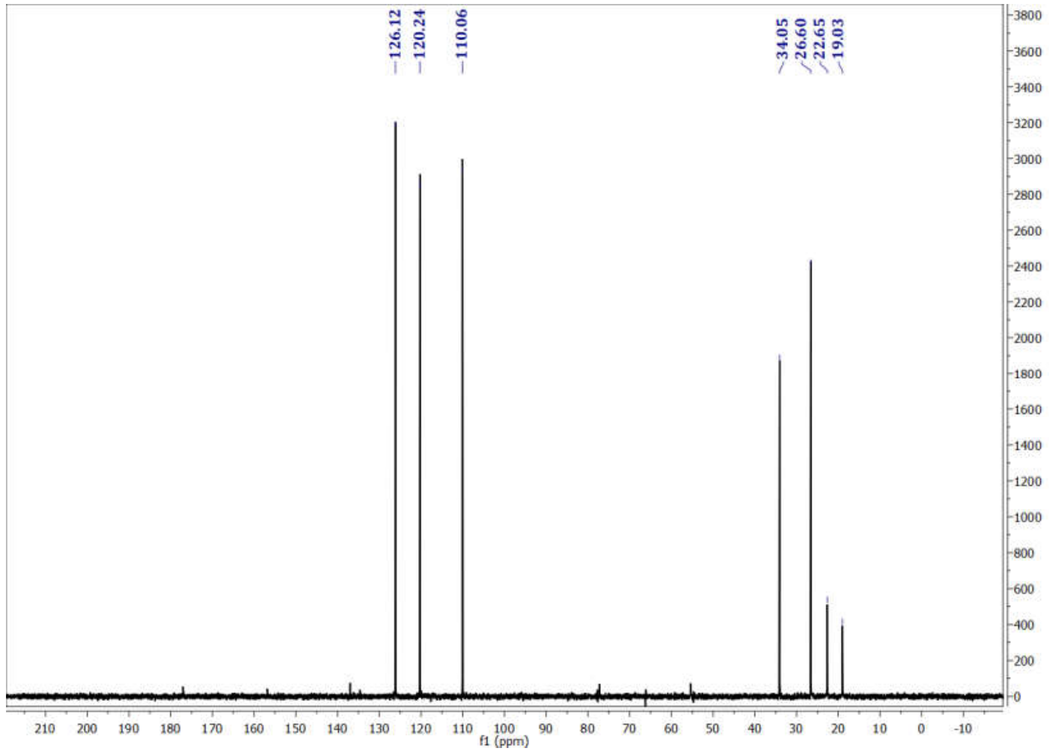

Figure S5. Distortionless enhancement by polarization transfer spectrum (DEPT90) of 3-methoxycuminyl isobutanoate (**7**).

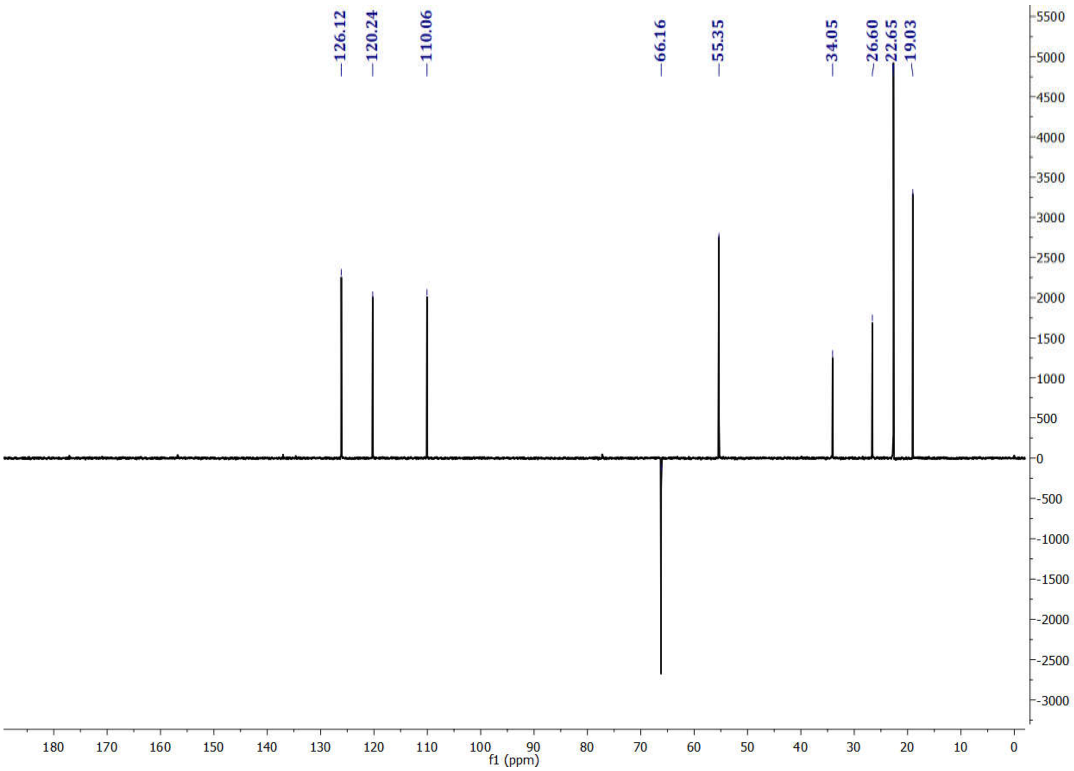

Figure S6. Distortionless enhancement by polarization transfer spectrum (DEPT135) of 3-methoxycuminyl isobutanoate (**7**).

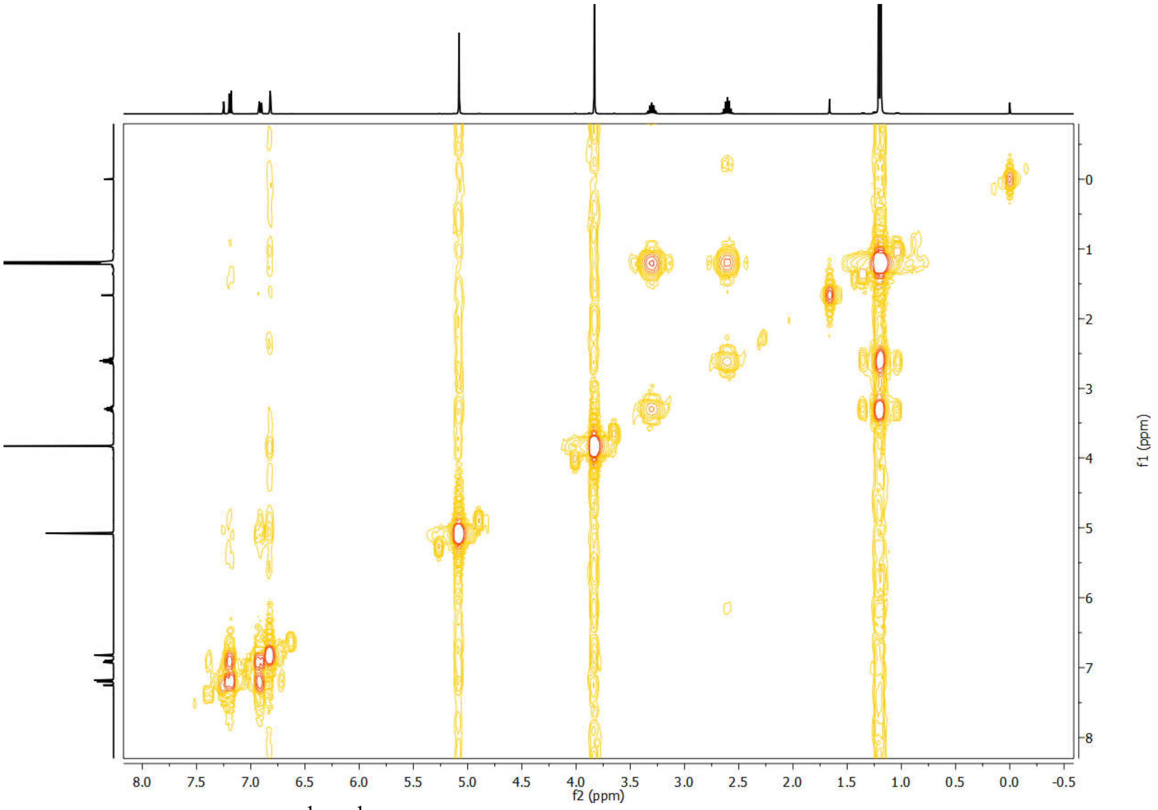

Figure S7. Gradient ^{1}H–^{1}H COSY spectrum of 3-methoxycuminyl isobutanoate (**7**).

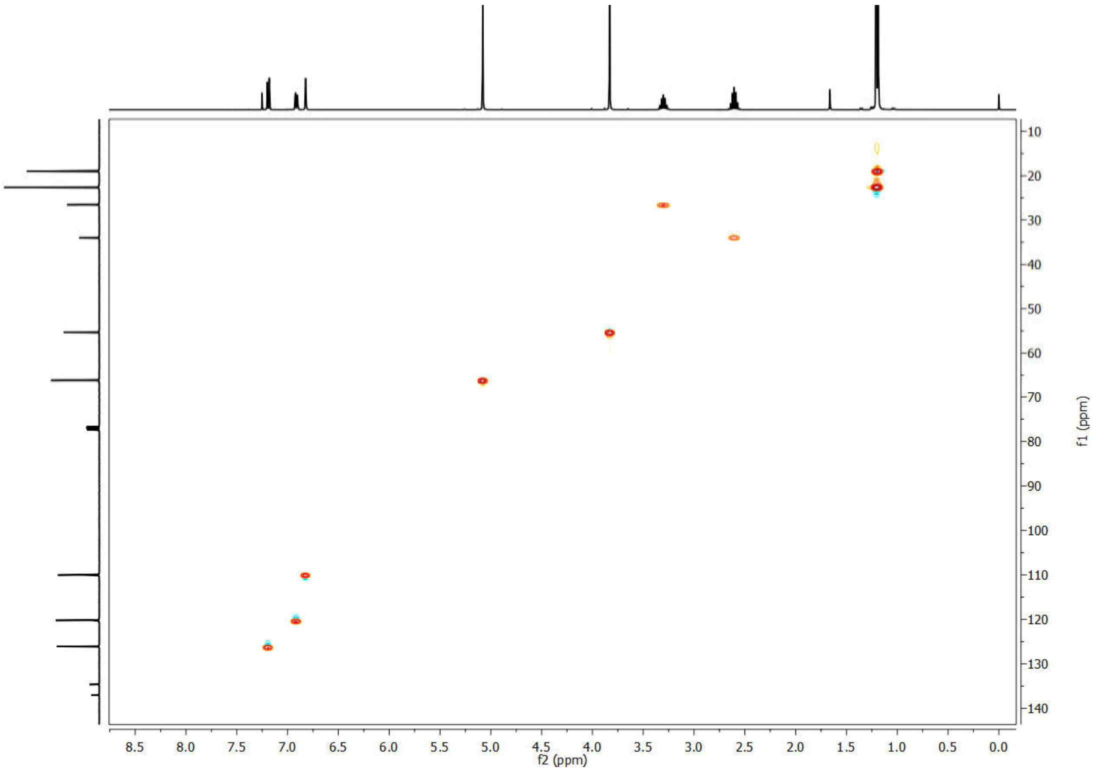

Figure S8. Gradient HSQC spectrum of 3-methoxycuminyl isobutanoate (**7**).

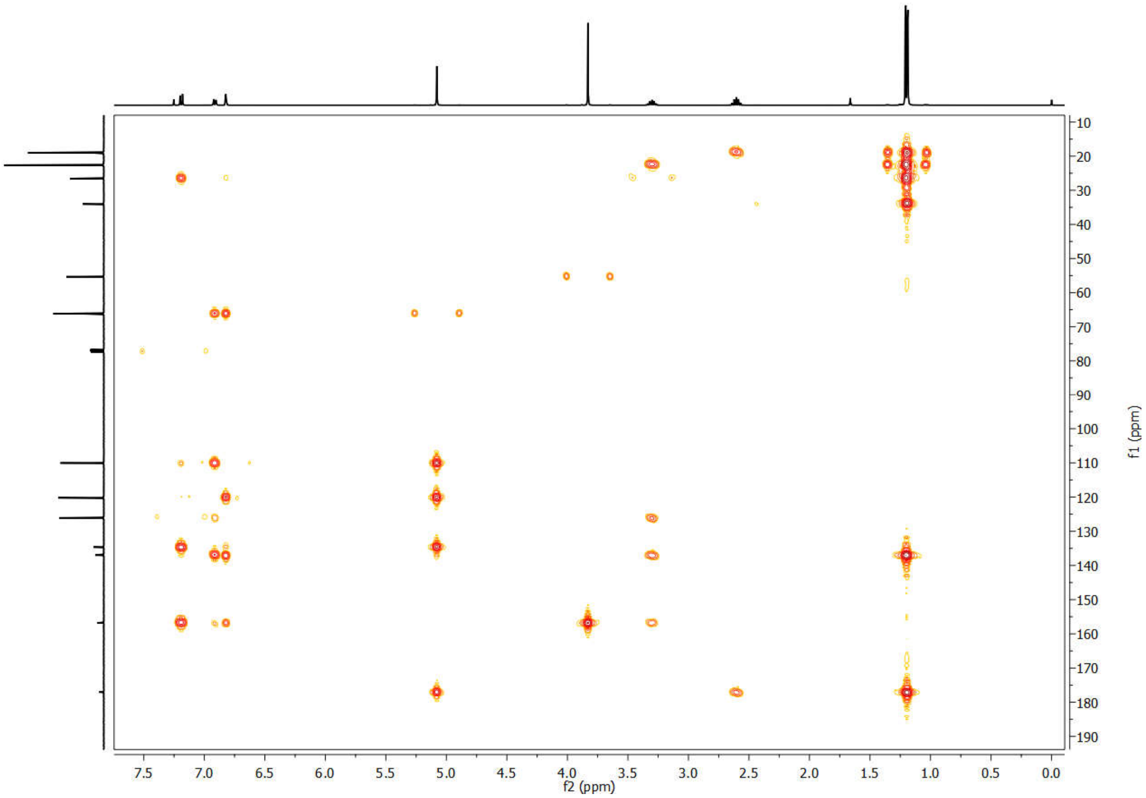

Figure S9. Gradient HMBC spectrum of 3-methoxycuminyl isobutanoate (**7**).

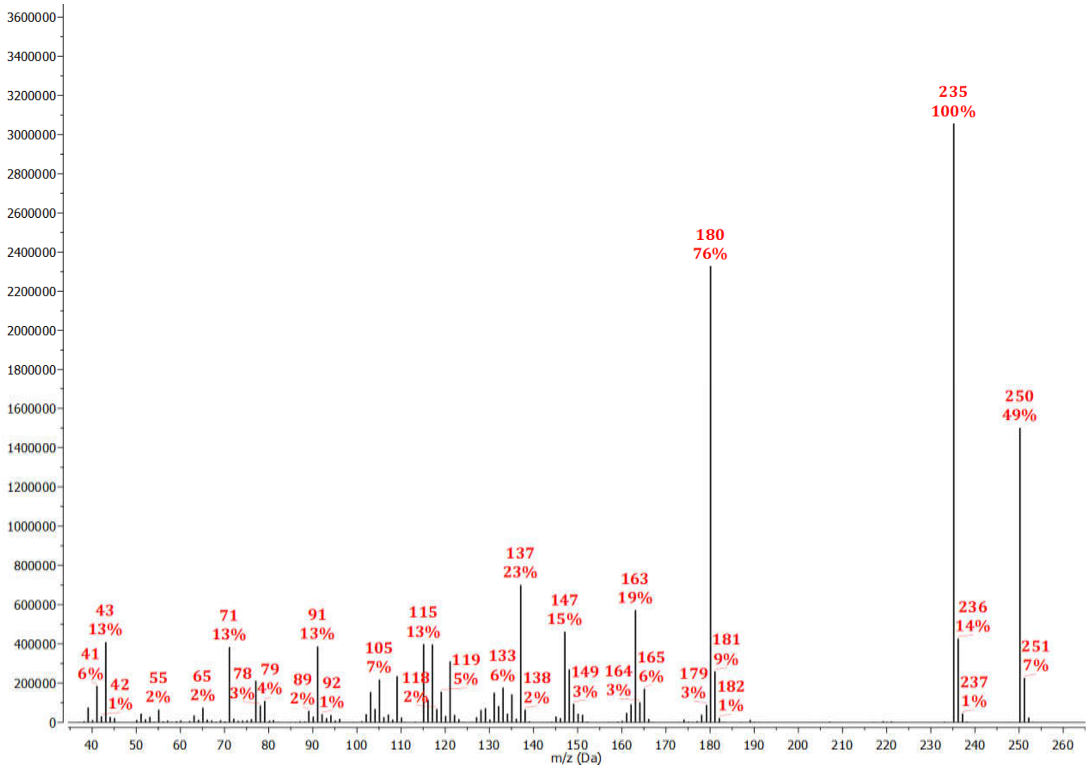

Figure S10. Mass spectrum of 3-methoxycuminyl butanoate (**8**).

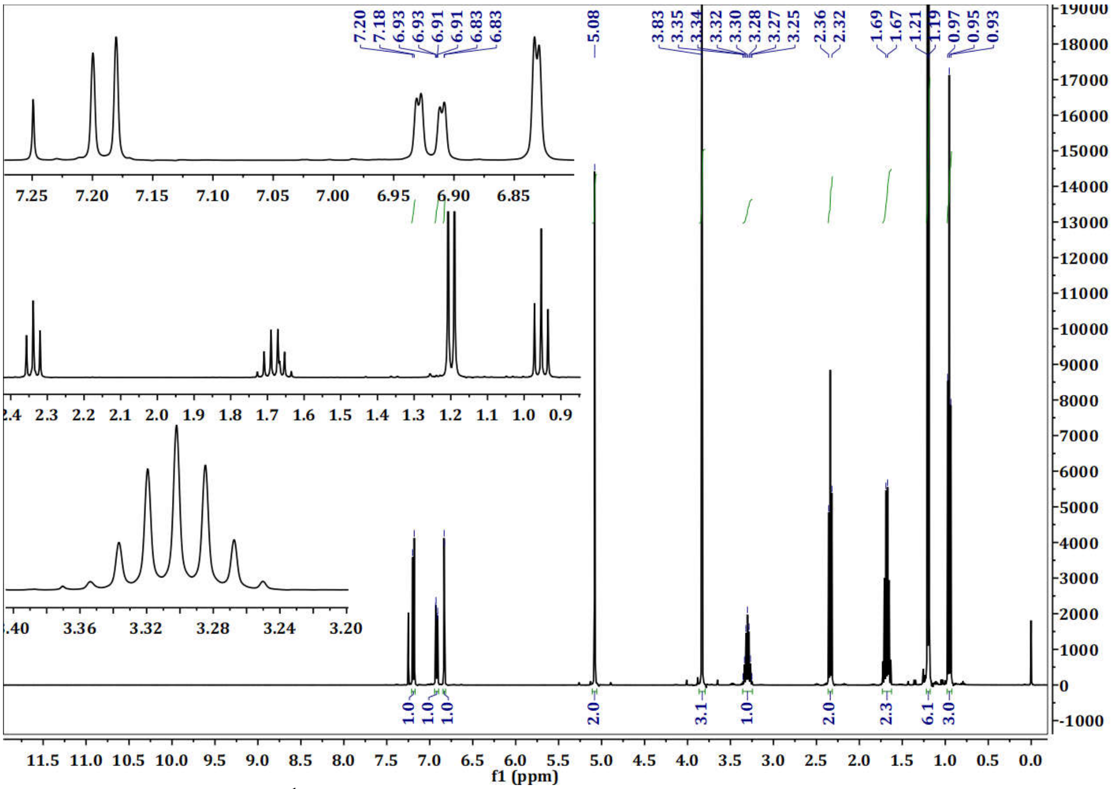

Figure S11. ^{1}H NMR spectrum of 3-methoxycuminyl butanoate (**8**).

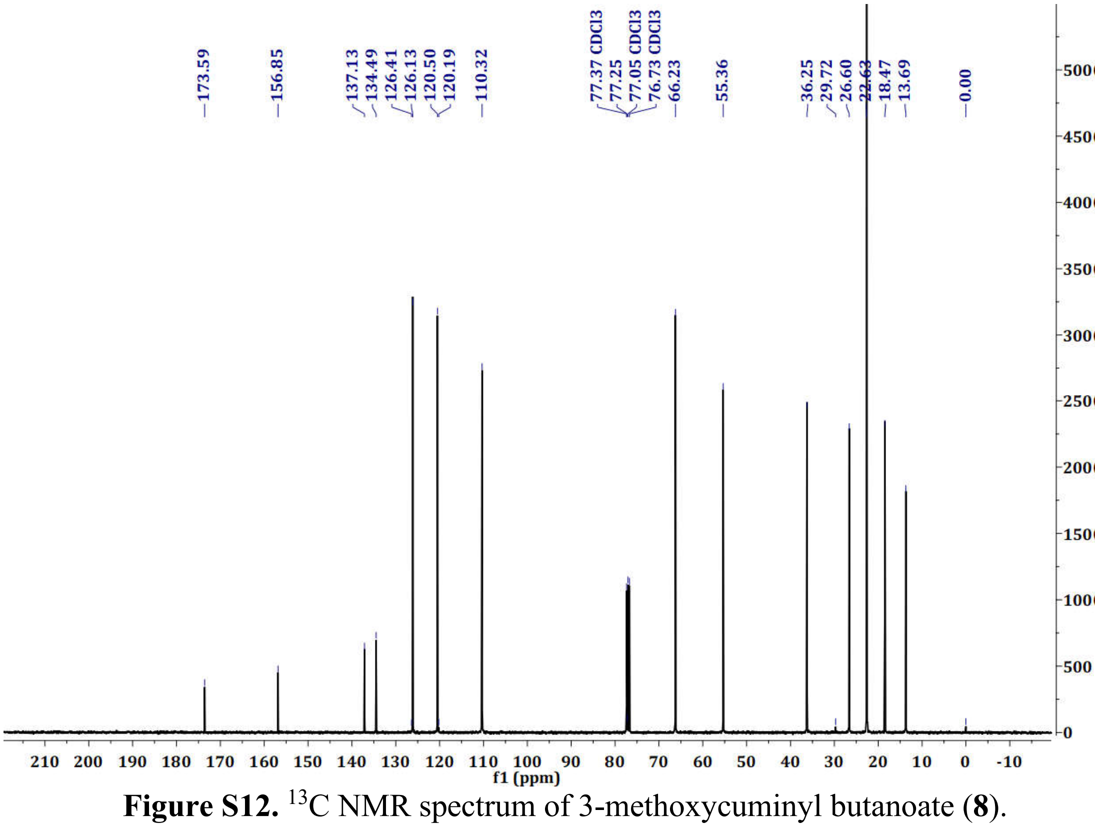

Figure S12. ^{13}C NMR spectrum of 3-methoxycuminyl butanoate (**8**).

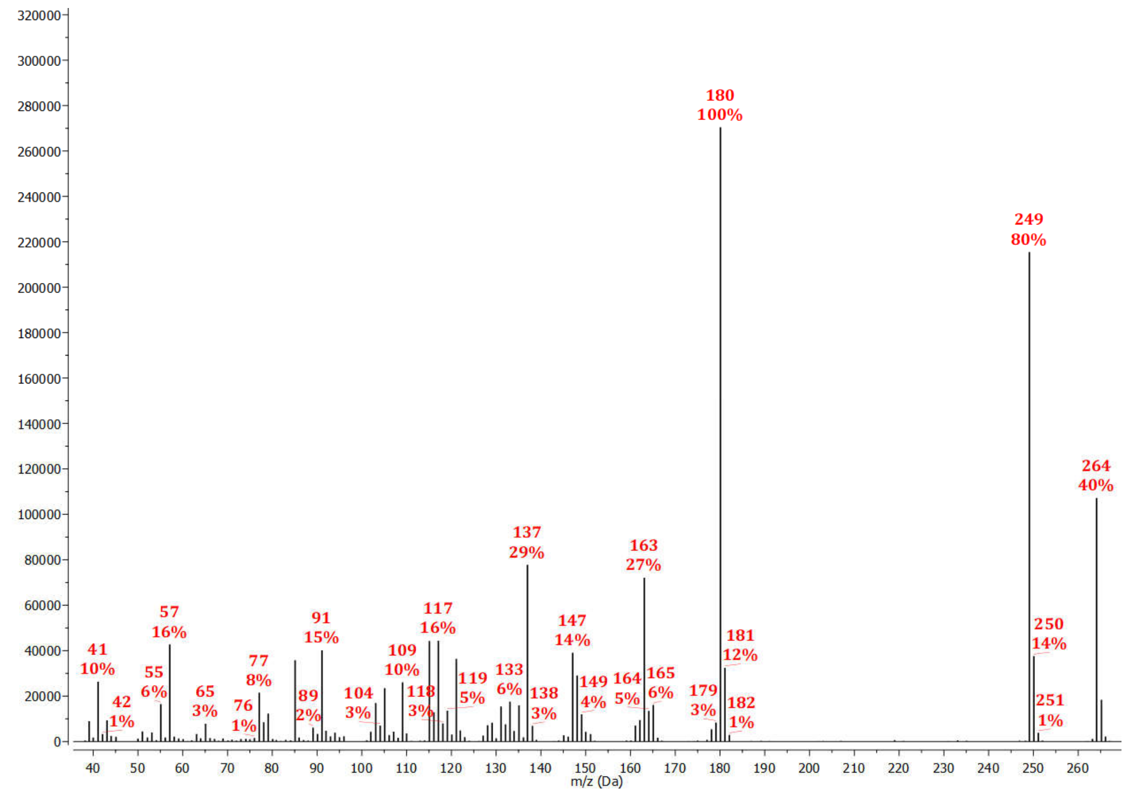

Figure S13. Mass spectrum of 3-methoxycuminyl 2-methylbutanoate (**9**).

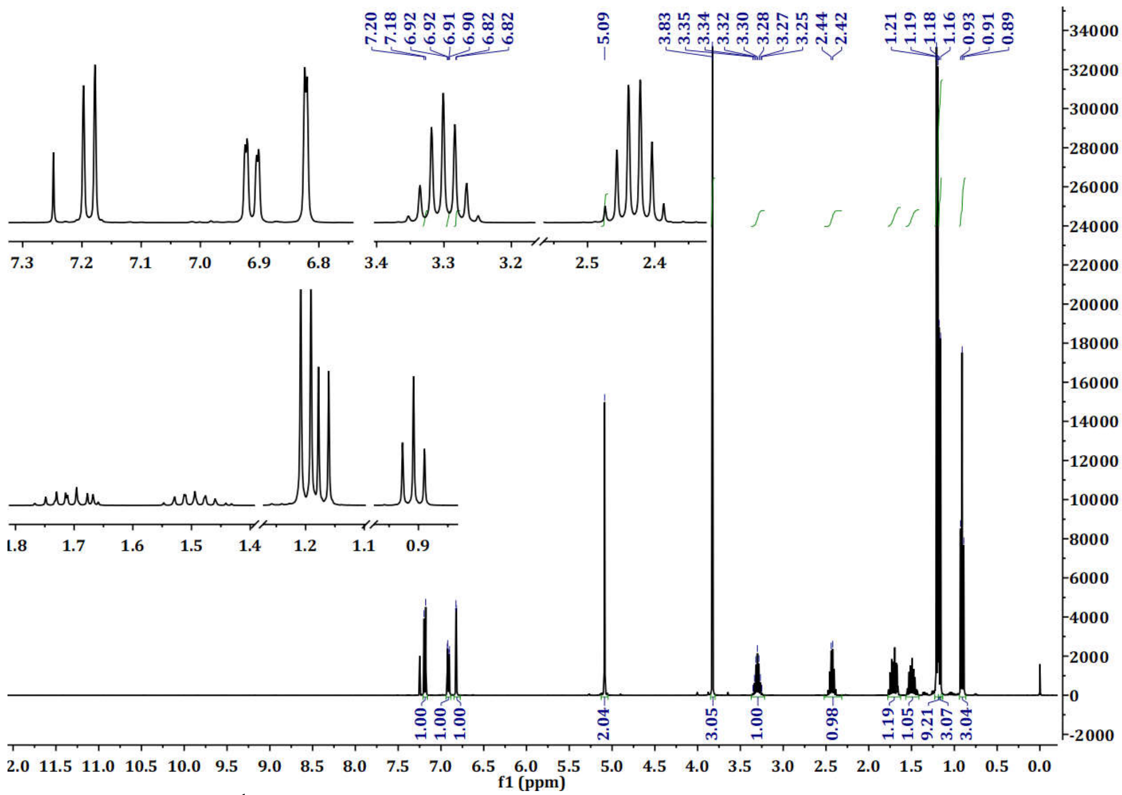

Figure S14. ^{1}H NMR spectrum of 3-methoxycuminyl 2-methylbutanoate (**9**).

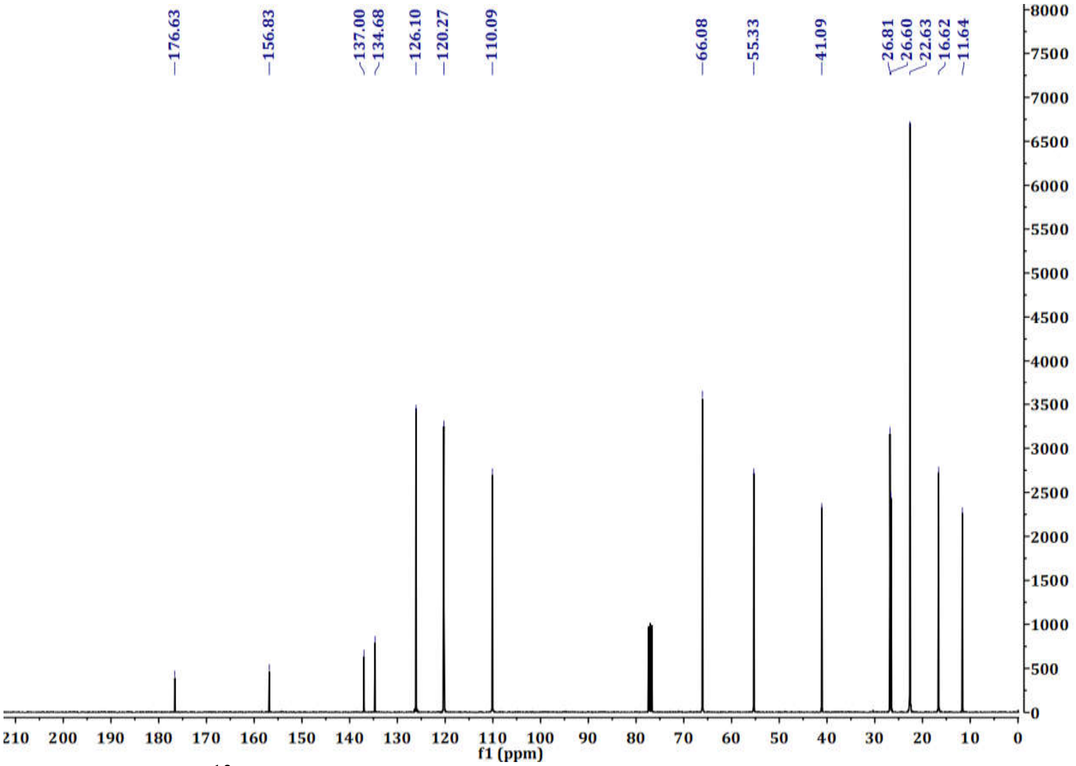

Figure S15. ^{13}C NMR spectrum of 3-methoxycuminyl 2-methylbutanoate (**9**).

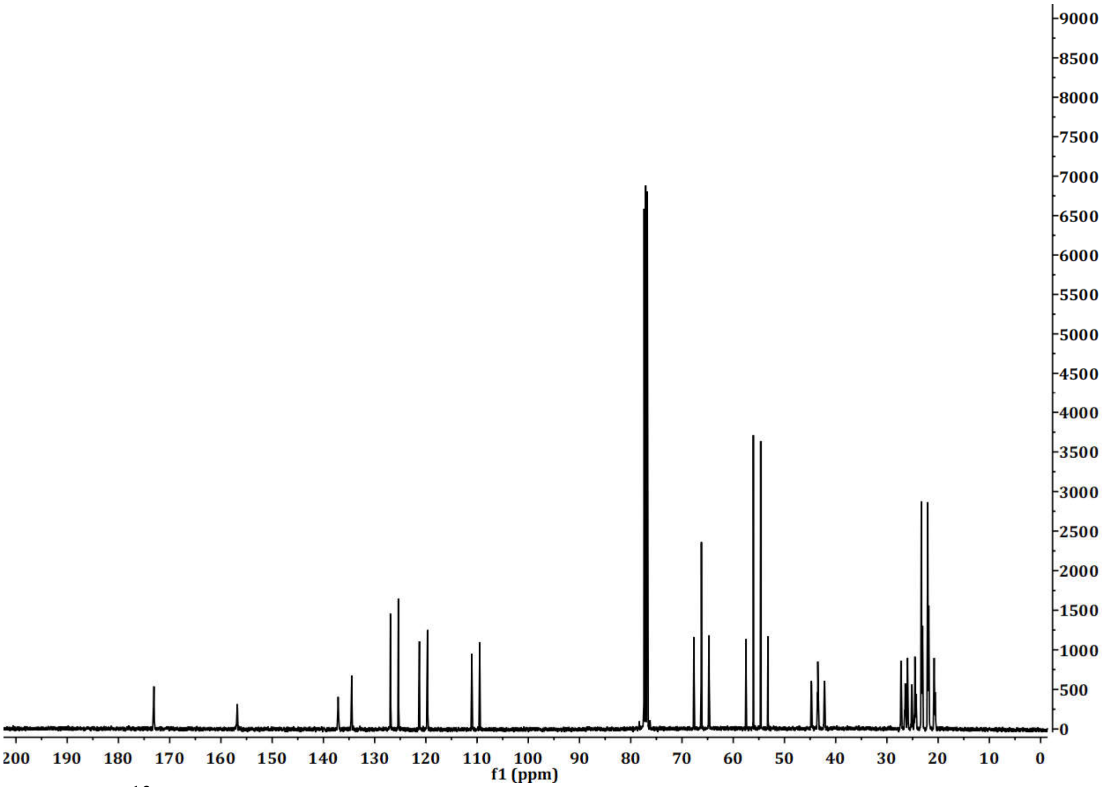

Figure S16. ^{13}C NMR spectrum (without heteronuclear decoupling) of 3-methoxycuminyl 2-methylbutanoate (**9**).

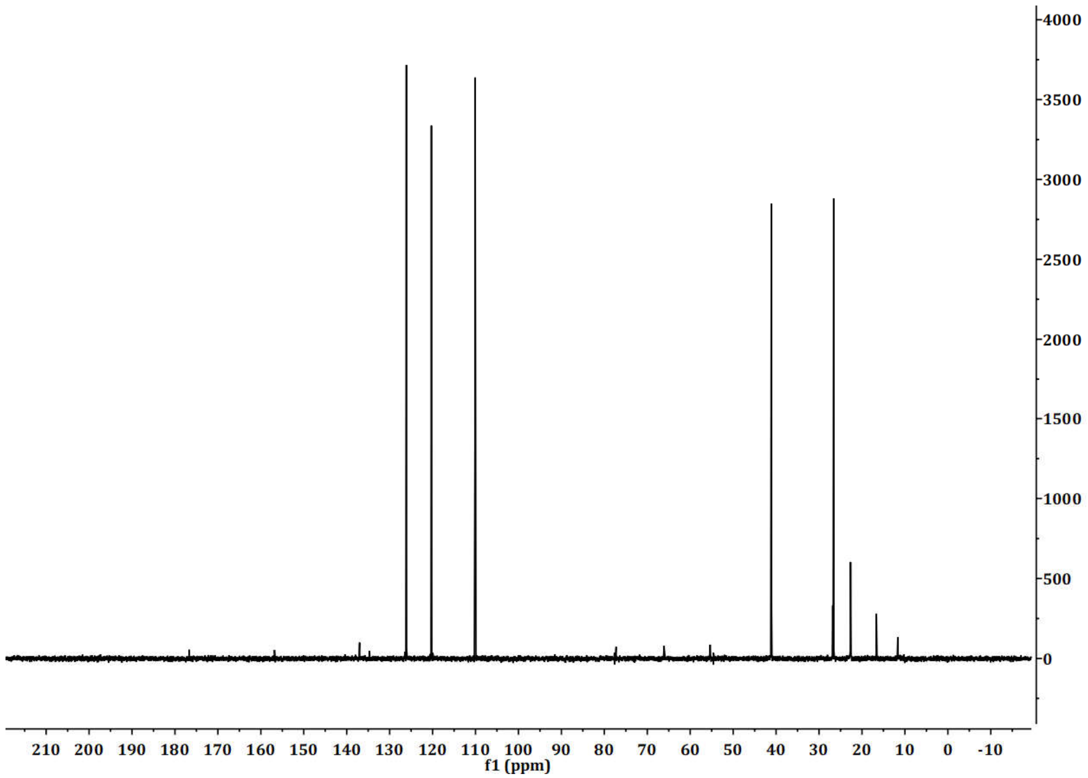

Figure S17. Distortionless enhancement by polarization transfer spectrum (DEPT90) of 3-methoxycuminyl 2-methylbutanoate (**9**).

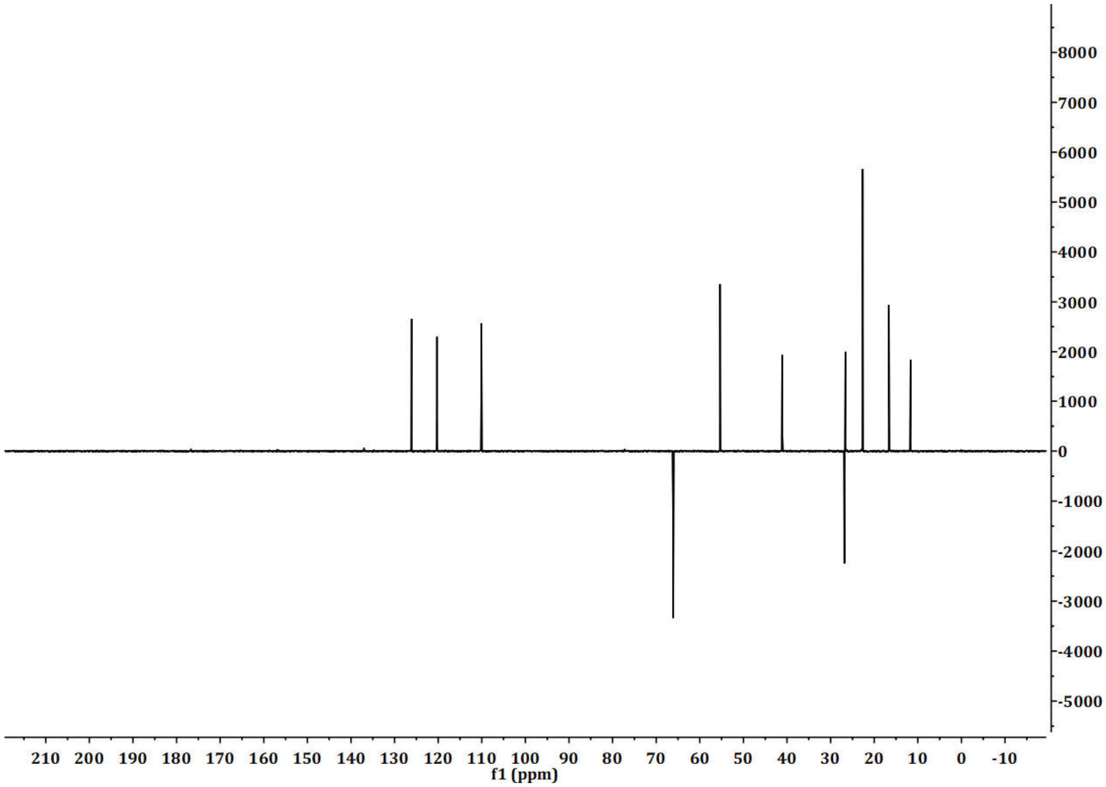

Figure S18. Distortionless enhancement by polarization transfer spectrum (DEPT135) of 3-methoxycuminyl 2-methylbutanoate (**9**).

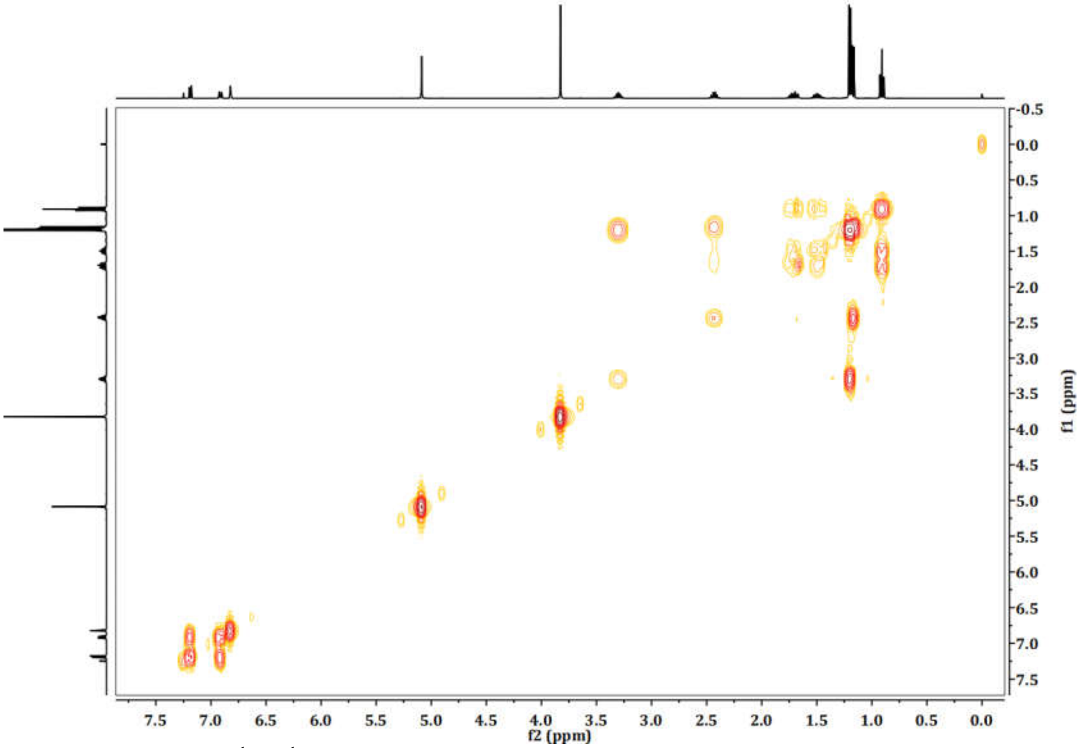

Figure S19. Gradient ^{1}H–^{1}H COSY spectrum of 3-methoxycuminyl 2-methylbutanoate (**9**).

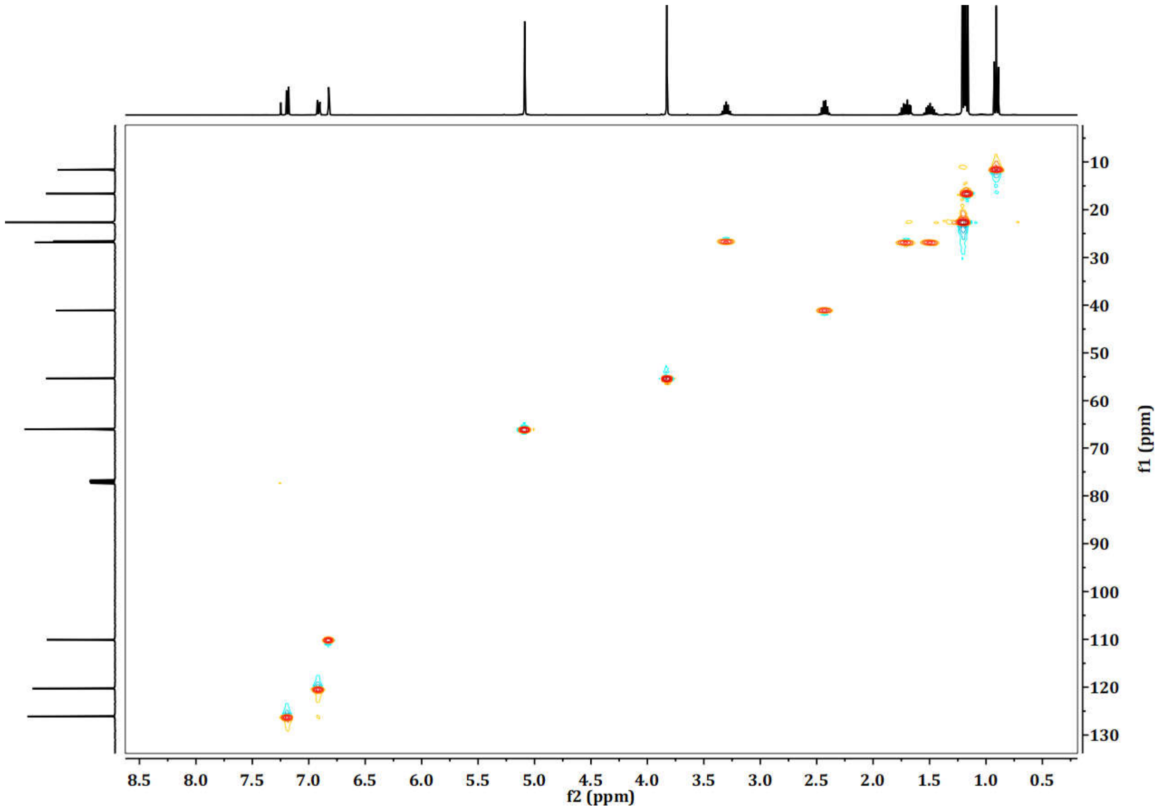

Figure S20. Gradient HSQC spectrum of 3-methoxycuminyl 2-methylbutanoate (**9**).

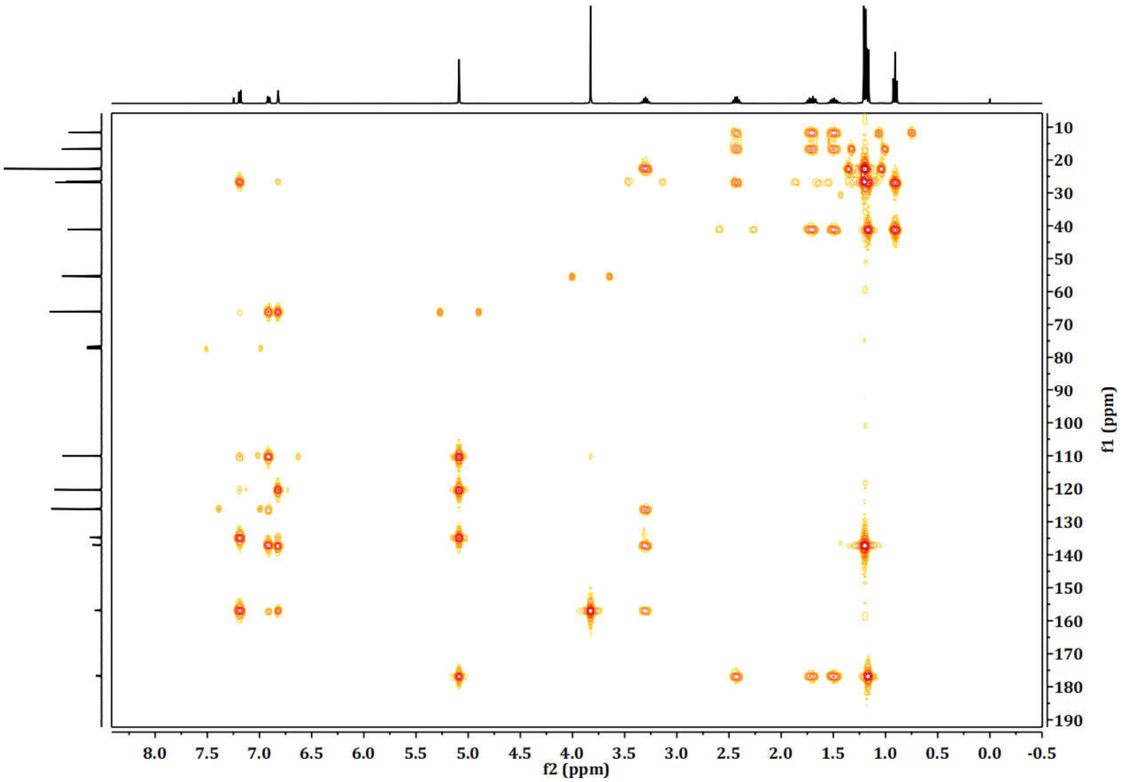

Figure S21. Gradient HMBC spectrum of 3-methoxycuminyl 2-methylbutanoate (**9**).

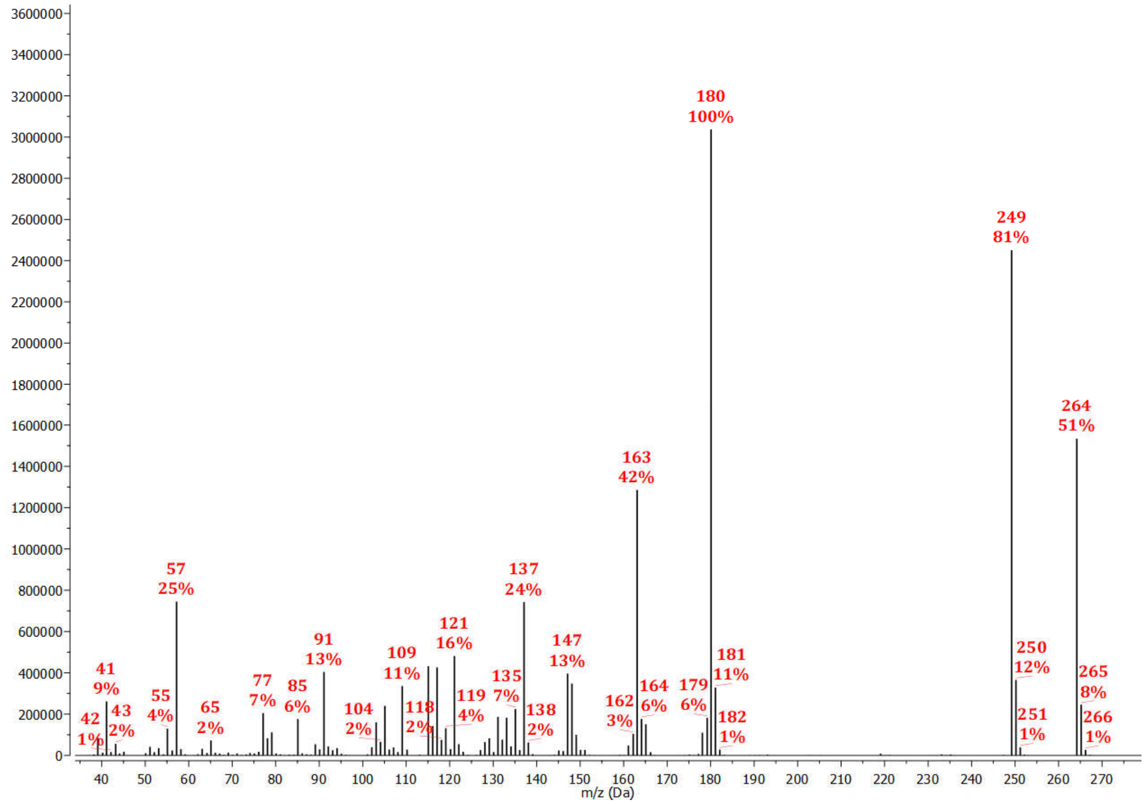

Figure S22. Mass spectrum of 3-methoxycuminyl 3-methylbutanoate (**10**).

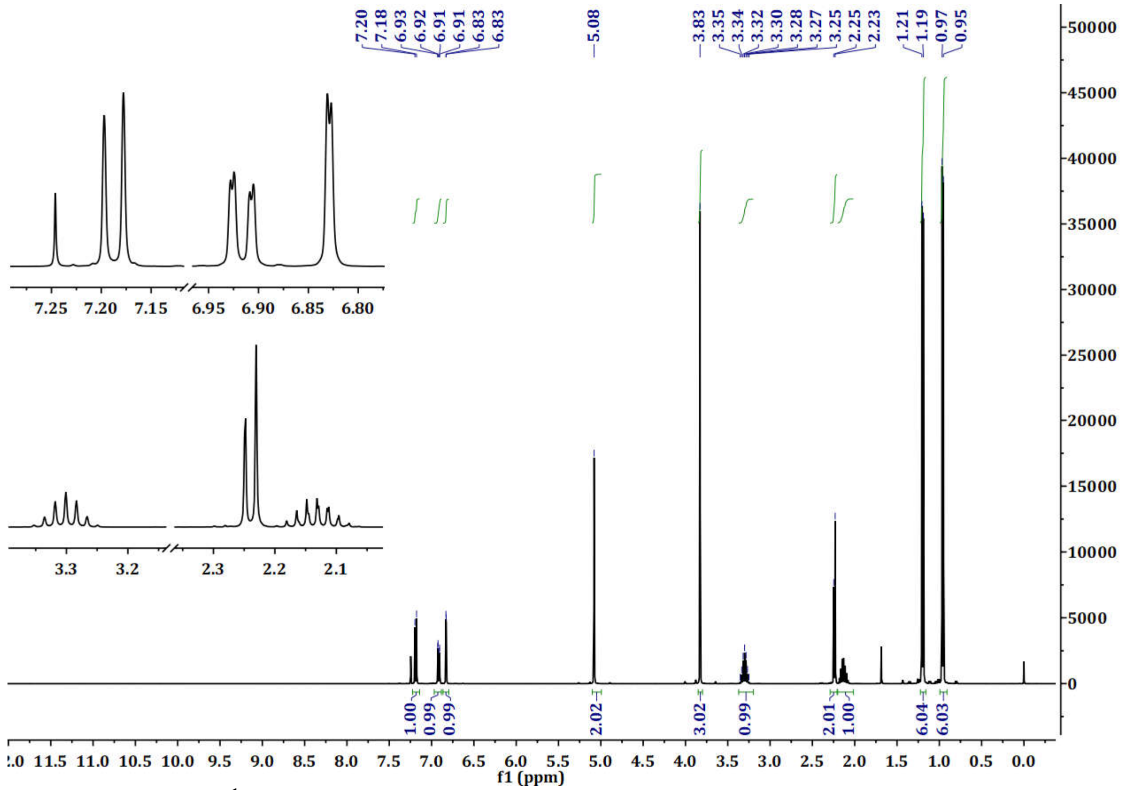

Figure S23. ^{1}H NMR spectrum of 3-methoxycuminyl 3-methylbutanoate (**10**).

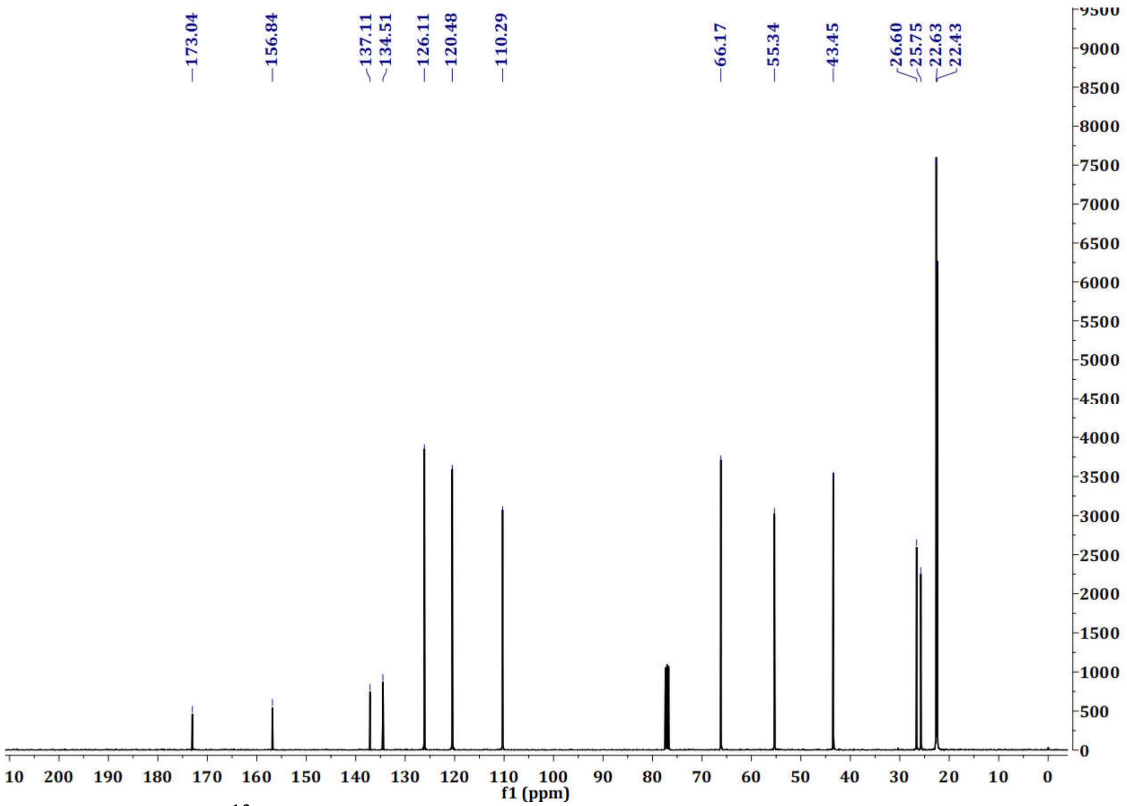

Figure S24. ^{13}C NMR spectrum of 3-methoxycuminyl 3-methylbutanoate (**10**).

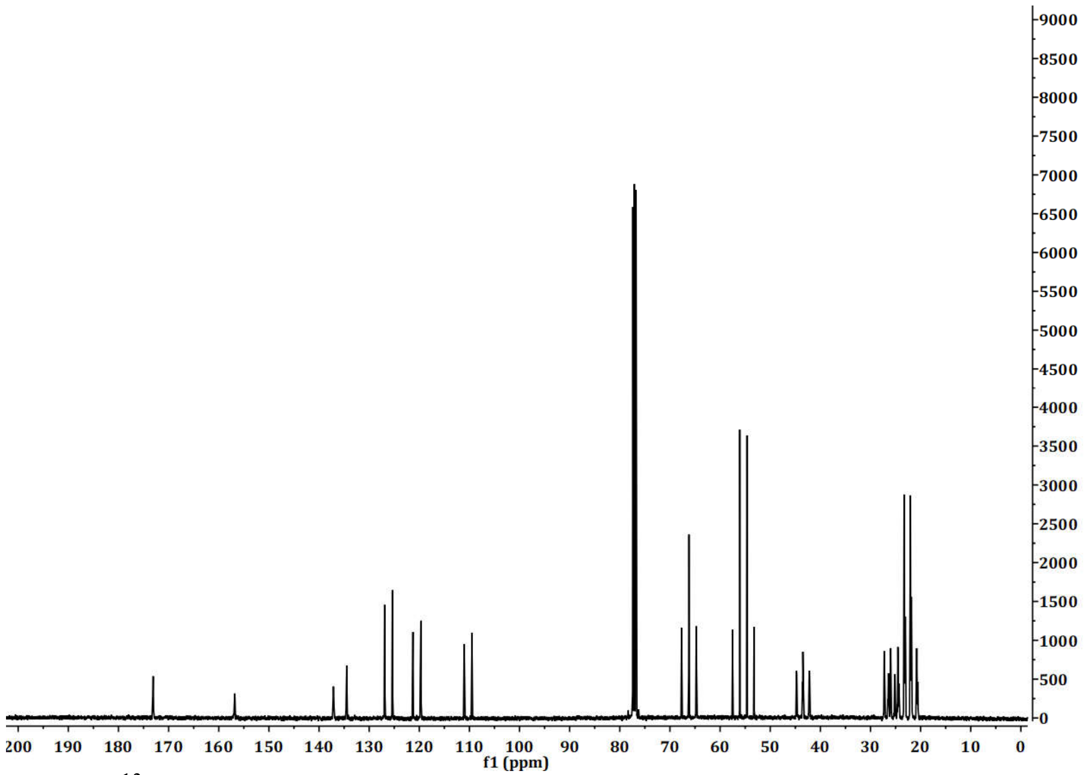

Figure S25. ^{13}C NMR spectrum (without heteronuclear decoupling) of 3-methoxycuminyl 3-methylbutanoate (**10**).

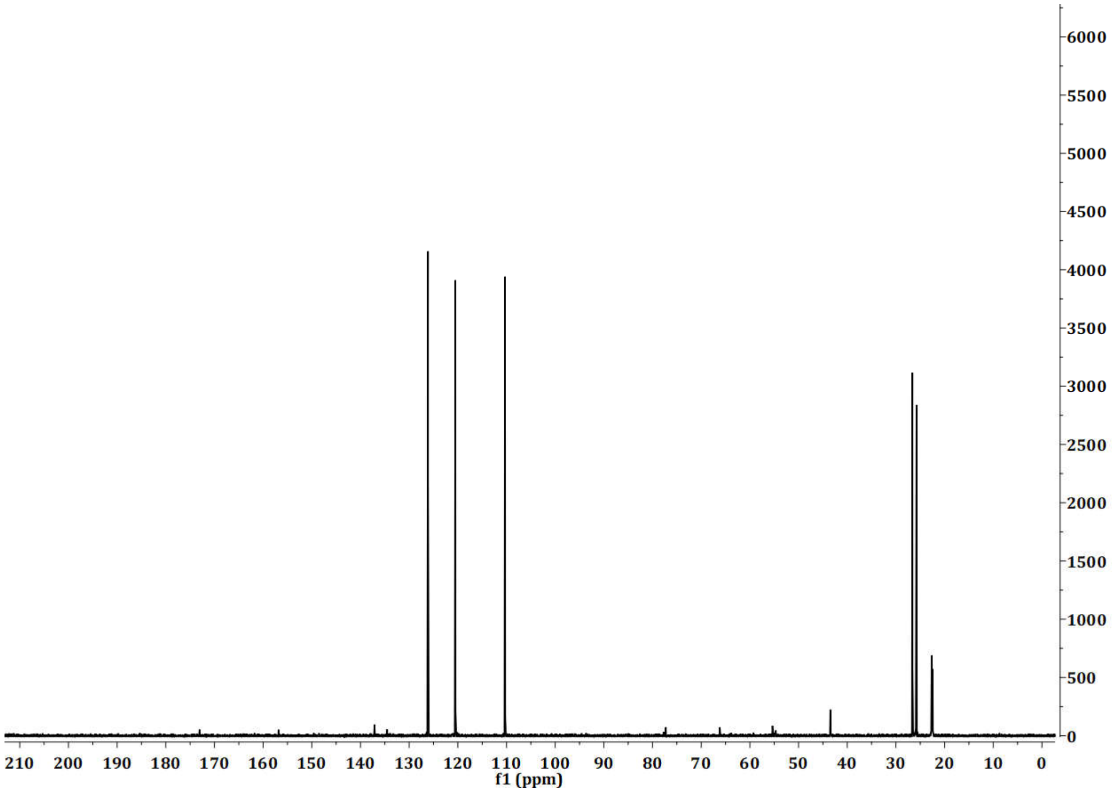

Figure S26. Distortionless enhancement by polarization transfer spectrum (DEPT90) of 3-methoxycuminyl 3-methylbutanoate (**10**).

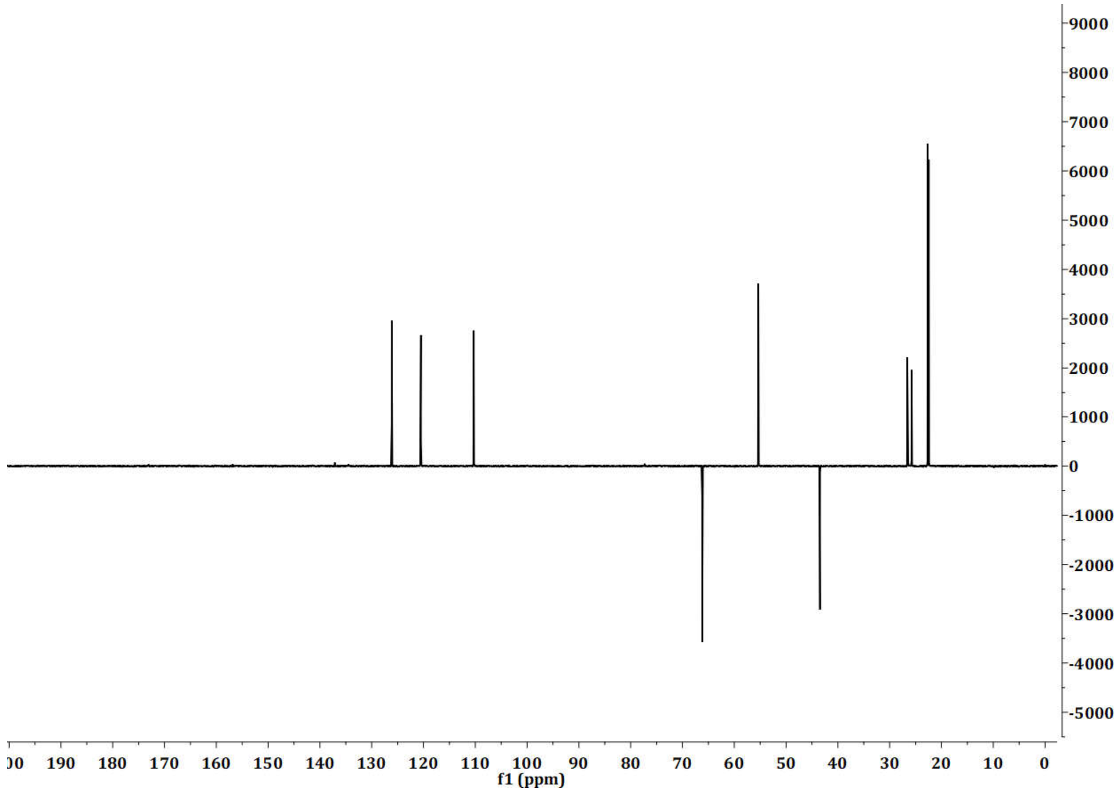

Figure S27. Distortionless enhancement by polarization transfer spectrum (DEPT135) of 3-methoxycuminyl 3-methylbutanoate (**10**).

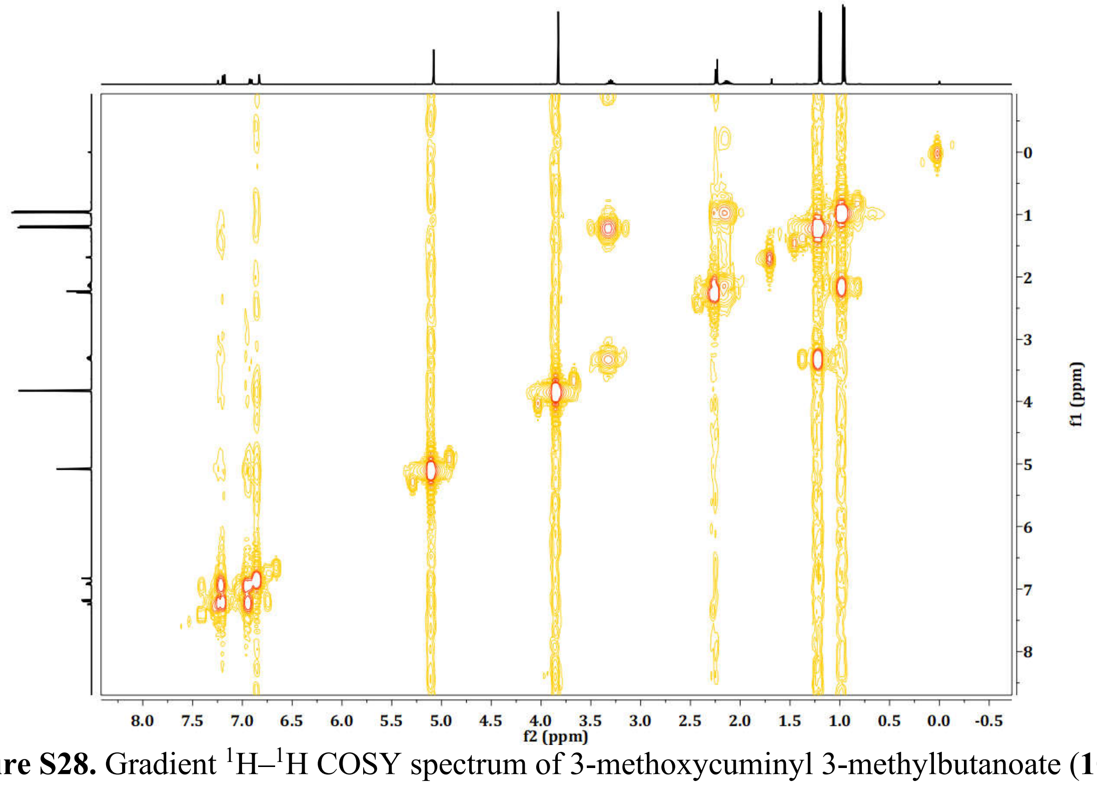

Figure S28. Gradient ^1H–^1H COSY spectrum of 3-methoxycuminyl 3-methylbutanoate (**10**).

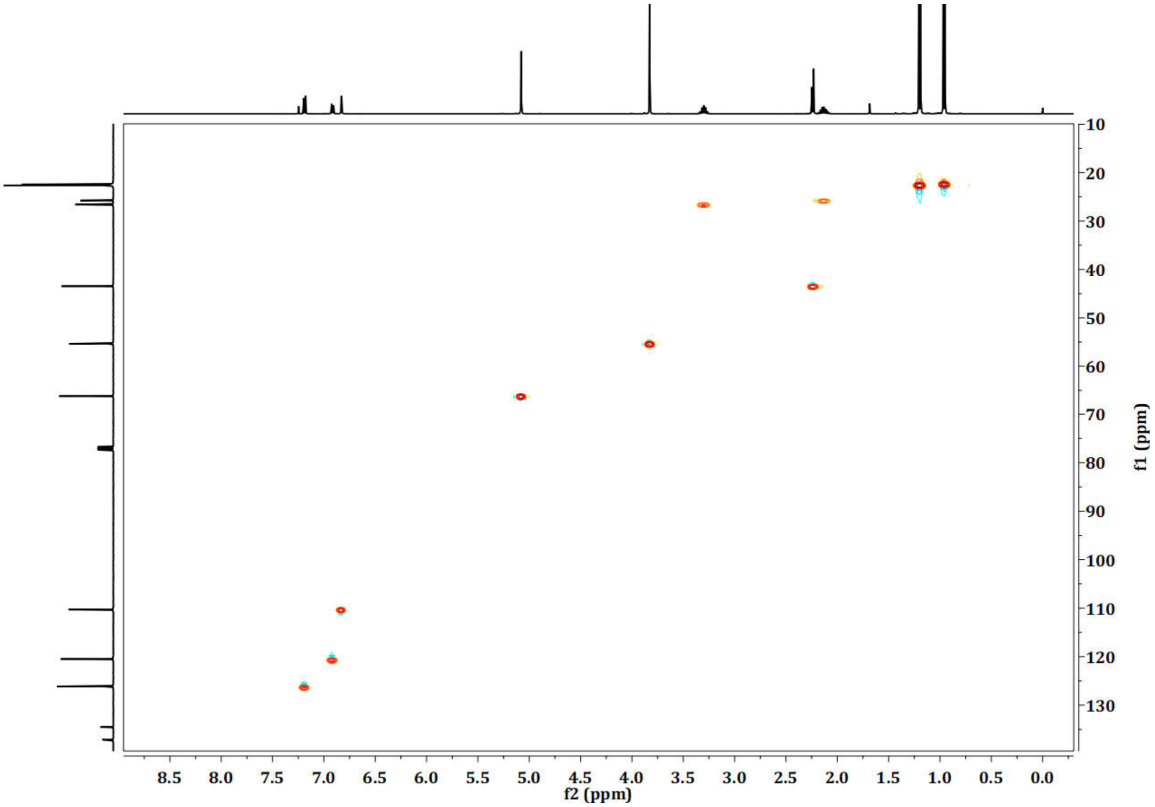

Figure S29. Gradient HSQC spectrum of 3-methoxycuminyl 3-methylbutanoate (**10**).

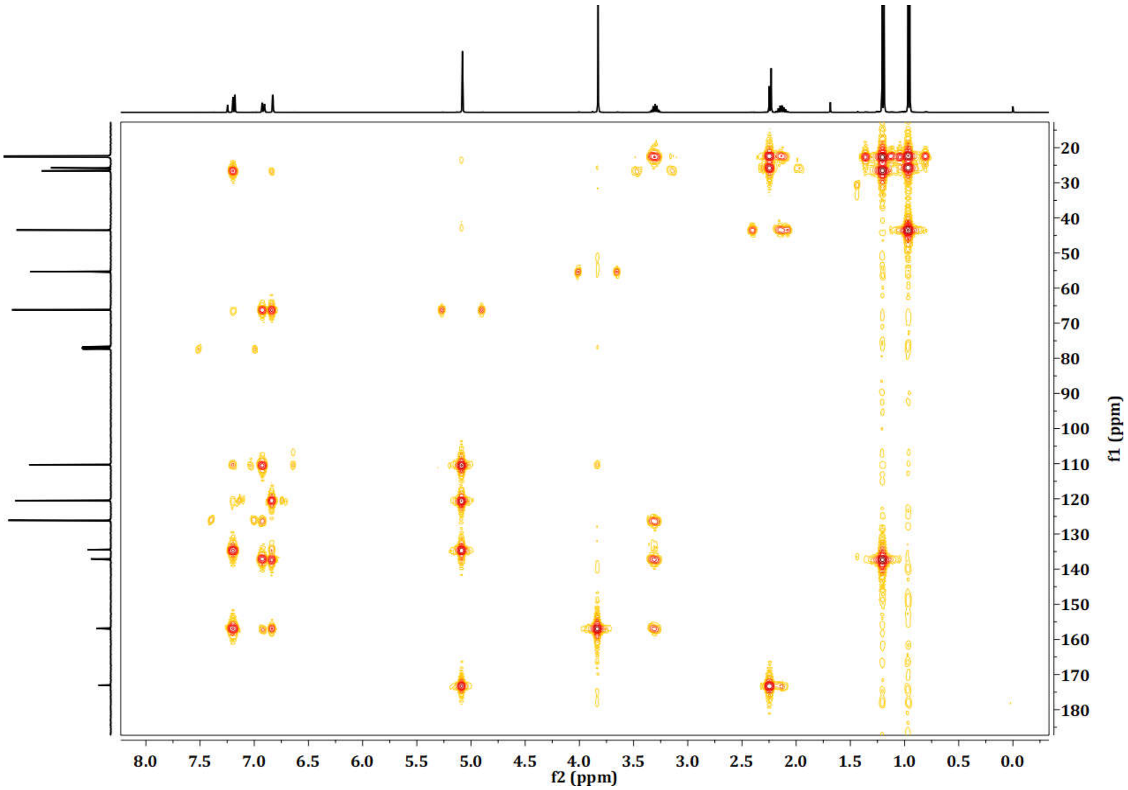

Figure S30. Gradient HMBC spectrum of 3-methoxycuminyl 3-methylbutanoate (**10**).

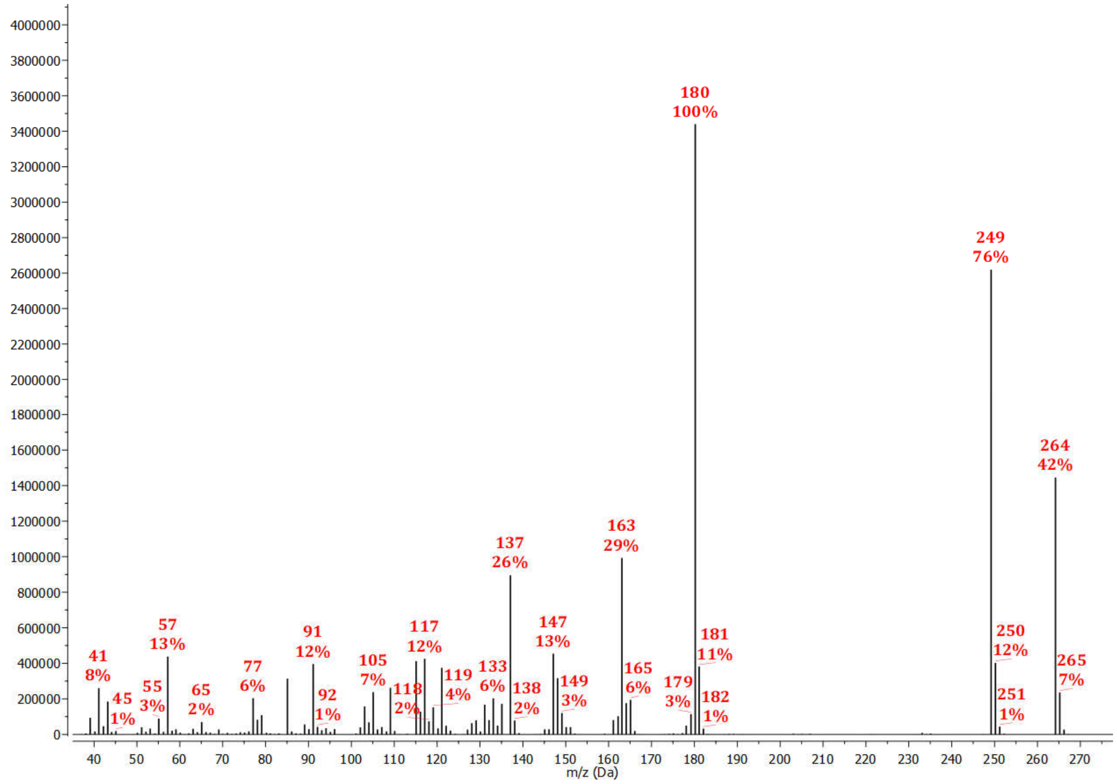

Figure S31. Mass spectrum of 3-methoxycuminyl pentanoate (**11**).

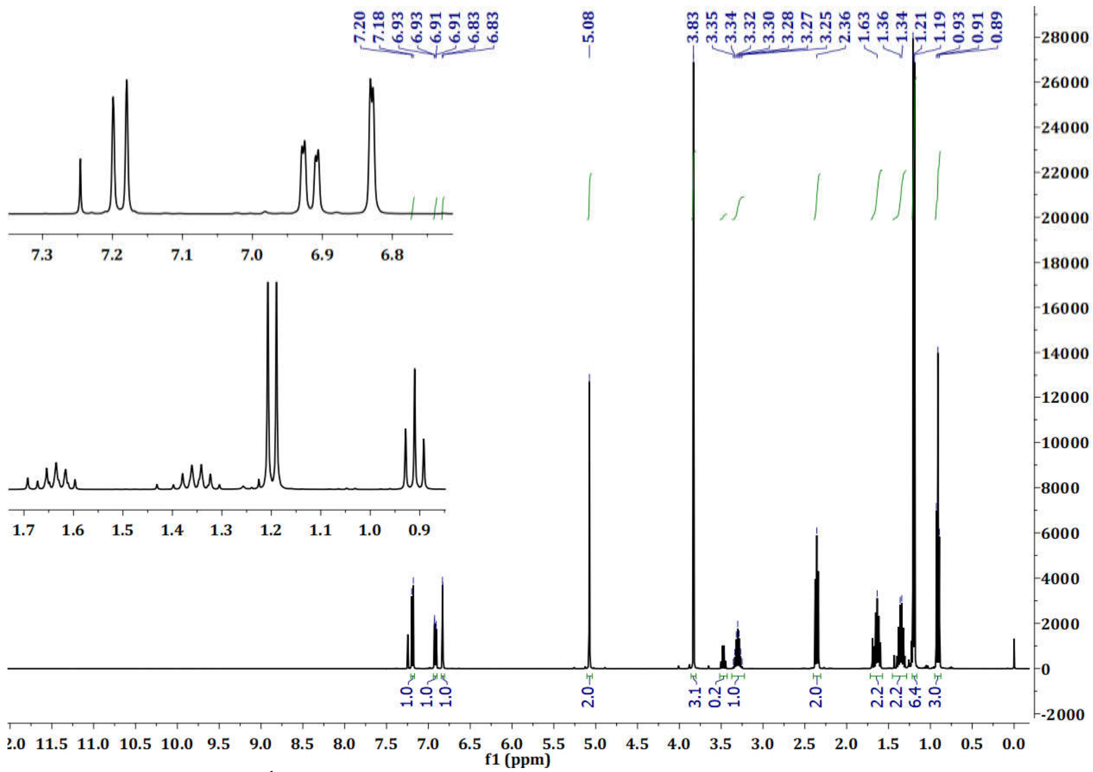

Figure S32. ^{1}H NMR spectrum of 3-methoxycuminyl pentanoate (**11**).

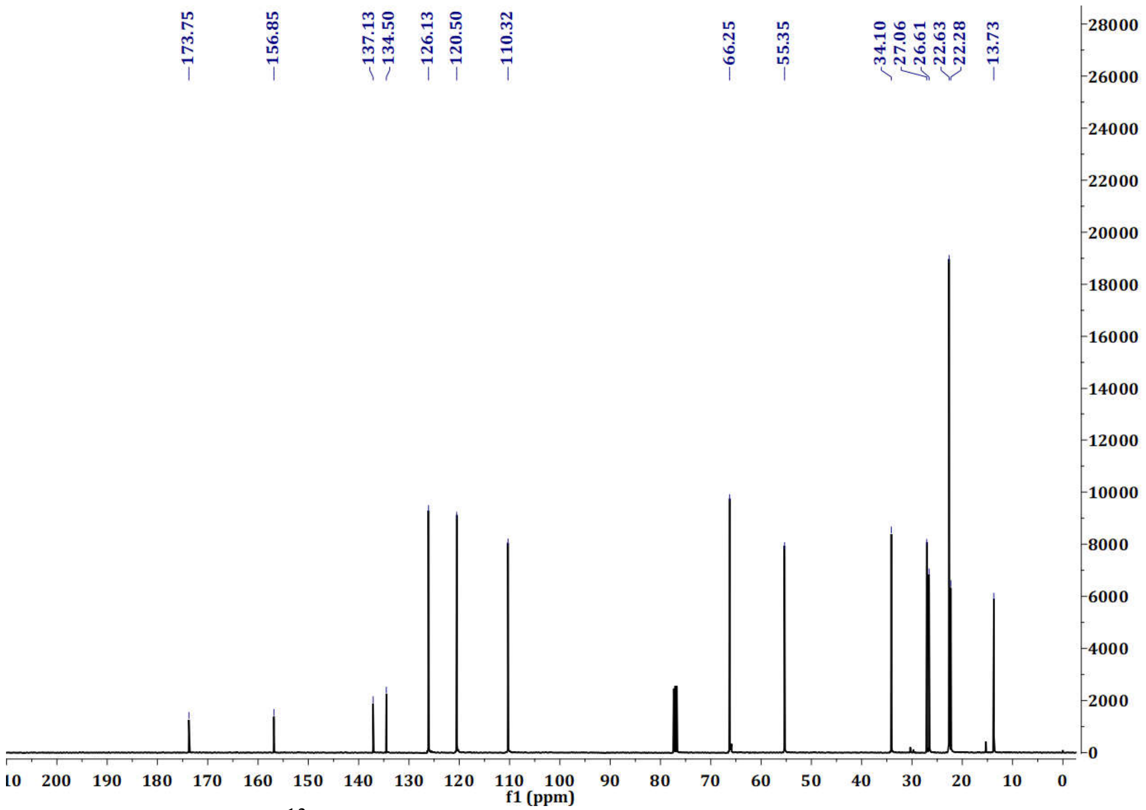

Figure S33. ^{13}C NMR spectrum of 3-methoxycuminyl pentanoate (**11**).

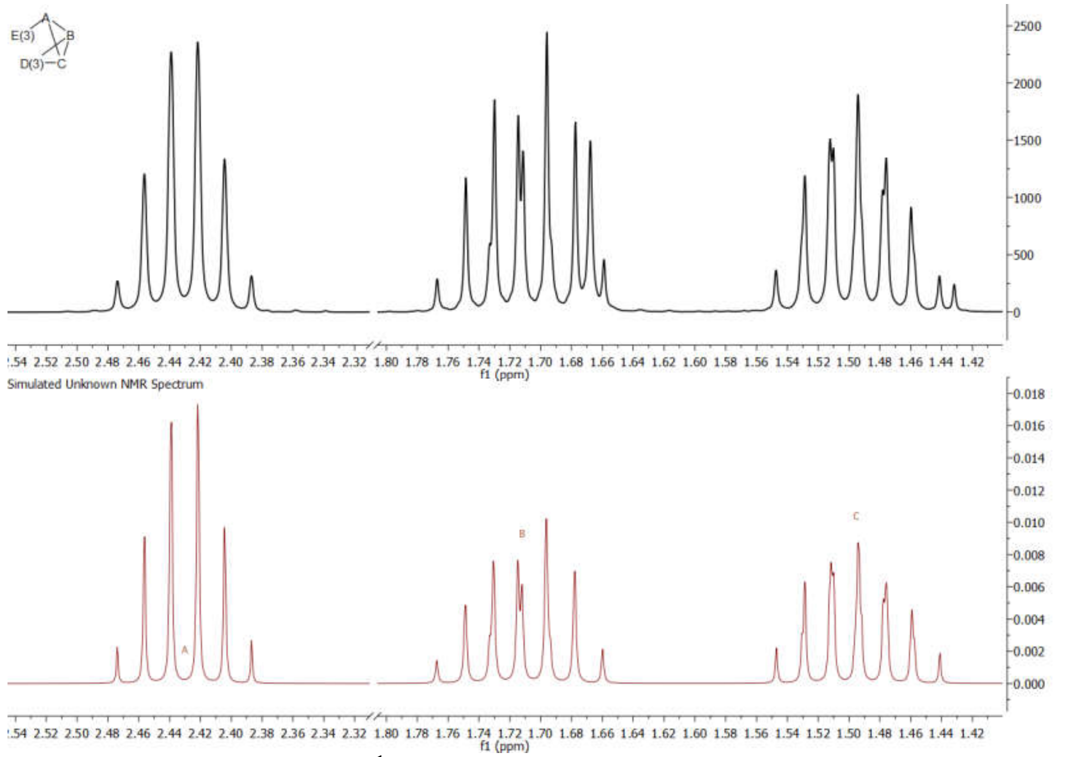

Figure S34. Spin simulation of the ^{1}H NMR signals for the protons at position 13 and 14 (lower) and comparison with the experimental ones (upper) for 3-methoxycuminyl 2-methylbutanoate (**9**).
